# Heliosterpenoids A and B, two Novel Jatrophane-Derived Diterpenoids with a 5/6/4/6 Ring System from *Euphorbia helioscopia*

**DOI:** 10.1038/s41598-017-04399-w

**Published:** 2017-07-07

**Authors:** Zhen-Peng Mai, Gang Ni, Yan-Fei Liu, Li Li, Guo-Ru Shi, Xin Wang, Jia-Yuan Li, De-Quan Yu

**Affiliations:** State Key Laboratory of Bioactive Substance and Function of Natural Medicines, Institute of Materia Medica, Chinese Academy of Medical Sciences and Peking Union Medical College, Beijing, 100050 P. R. China

## Abstract

Heliosterpenoids A and B (**1** and **2**), two unprecedented jatrophane-derived diterpenoid esters with a novel 5/6/4/6-fused tetracyclic ring skeleton, were isolated from the whole plants of *Euphorbia helioscopia*. The structures of **1** and **2** were elucidated by extensive spectroscopic methods and electronic circular dichroism (ECD) analyses. The plausible biogenetic pathways of **1** and **2** were postulated. **1** and **2** were found to be potent inhibitors of P-glycoprotein (ABCB1) and **1** also exhibited cytotoxicity against MDA-MB-231 cell lines.

## Introduction

**Figure 1:**
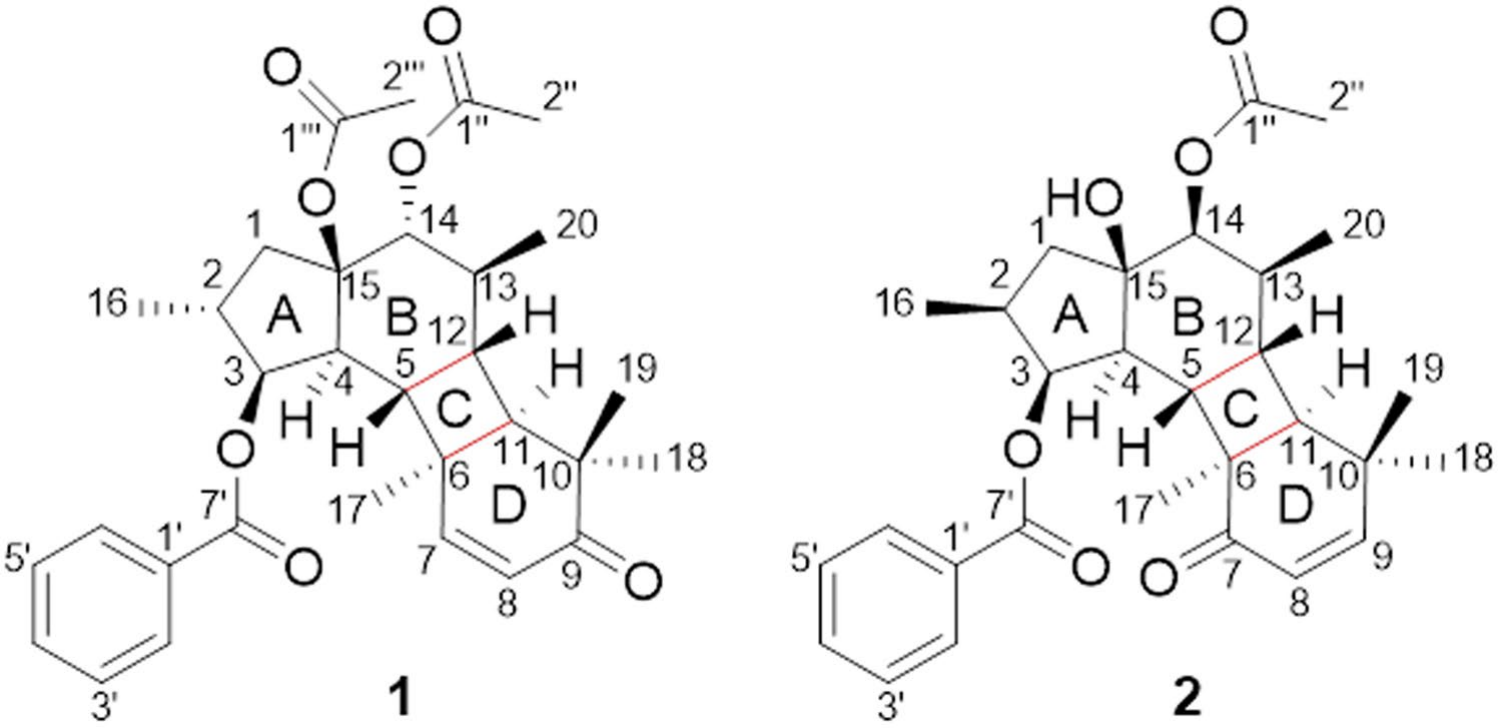
Structures of 1 and 2.

Macrocyclic diterpenes in the widespread genus *Euphorbia* are characteristic constituents and famous for diverse skeletons, such as jatrophanes, lathyranes, ingenanes, tiglianes, paralianes and segetanes^1^. Jatrophane diterpenes are the major components of *Euphorbia* diterpenoids and also considered to be very important biogenetic precursor of many macrocyclic diterpenes because pegetanes, paralianes, and peluanes can be derived from them by intramolecular cyclization^2–4^. The general structure of the jatrophane skeleton is characterized by a highly oxygenated trans-bicyclo[10.3.0]pentadecane framework wherein there were five methyls respectively located at C-2, C-6, C-10 × 2, C-13. And the methyl group at C-2 can be present in either an *R* or *S* configuration as well as the methyl group at C-13^5, 6^. Meanwhile, jatrophanes exert a large number of biological activities, such as modulation of P-glycoprotein (ABCB1), cytotoxic, antimalarial and antibacterial activities^7–10^. *Euphorbia helioscopia* L. belongs to the genus *Euphorbia* (Euphorbiaceae) and has been used as Chinese folk medicine for the treatment of malaria, bacillary dysentery, osteomyelitis and tumors^11^. Our efforts to identify structurally interesting biologically active compounds from the poisonous traditional Chinese medicine resulted in the isolation of heliosterpenoids A and B (**1** and **2**) (Fig. 1), along with two known jatrophane diterpenes, euphornin C and euphornin H (Supporting Information). Heliosterpenoids A and B (**1** and **2**) both possess an unusual cyclojatrophane (featured a 5/6/4/6-fused tetracyclic ring system) diterpene skeleton. Here, the isolation, structure elucidation, plausible biosynthetic pathway, as well as biological evaluation of compounds **1** and **2** were discussed in detail.

## Results and Discussion

Heliosterpenoid A (**1**), was isolated as a white, amorphous powder. Its molecular formula C_31_H_38_O_7_, corresponding to 13 degrees of unsaturation, was deduced from the (+)-HRESIMS of the quasimolecular ion peak at *m/z* 545.2518 [M + Na]^+^ (calcd for C_31_H_38_O_7_Na, 545.2510). The IR spectrum suggested the presence of ester carbonyl (1743 cm^−1^), carbonyl (1715 cm^−1^), *α*,*β*-unsaturated keton (1675 cm^−1^) as well as a characteristic band for the aromatic ring (1602 and 1454 cm^−1^) functionalities. Based on those known jatrophane-skeleton diterpenoids with acyloxy groups from *E. helioscopia* ^5, 9, 12–14^, ^1^H and ^13^C NMR resonances of **1** showed a set of typical signals for polyesterified jatrophane-type diterpene nature as well (Table 1). The ^1^H NMR spectrum of **1** in CDCl_3_ implied a benzoyl group [*δ*_H_ 8.05 (2 H, dd, *J* = 7.8, 1.2 Hz, H-2′ and H-6′), 7.60 (1 H, t, *J* = 7.8 Hz, H-4′), and 7.47 (2 H, t, *J* = 7.8 Hz, H-3′ and H-5′)], a pair of *cis* olefinic protons [*δ*_H_ 6.45 (1 H, dd, *J* = 10.2, 2.4 Hz, H-7), and 5.86 (1 H, d, *J* = 10.2 Hz, H-8)] and two oxygenated methine protons [*δ*_H_ 5.62 (1 H, d, *J* = 1.8 Hz, H-14), and 5.26 (1 H, dd, *J* = 7.2, 2.4 Hz, H-3)]. In addition, five tertiary methyl groups [*δ*_H_ 2.14 (H_3_-2″′), 2.01 (H_3_-2″), 1.37 (H_3_-17), 1.07 (H_3_-18), and 0.98 (H_3_-19)], two secondary methyl group [*δ*_H_ 1.26 (d, *J* = 7.2 Hz, H_3_-16), and 0.96 (d, *J* = 7.2 Hz, H_3_-20)] were also displayed in the ^1^H NMR spectrum of **1**. Analyses of the ^13^C NMR and DEPT spectra of **1**, indicated the existence of 31 carbons. In combination with the aforementioned proton signals, the following typical carbon resonances [*δ*_C_ 170.1, and 22.5; 169.3, and 21.6; and 166.1, 130.4, 129.6 × 2, 128.7 × 2, and 133.3] suggested that **1** was a jatrophane triester bearing two acetoxy and a benzoyloxy groups. Besides the above 11 carbon resonances, the remaining 20 carbon

**Table 1:** ^1^H NMR (600 MHz) and ^13^C NMR (150 MHz) Data of Heliosterpenoid A and B (*δ* in ppm, and *J* in Hz).

No	1^*a*^		2^*a*^		2^*b*^	
	*δ*_H_	*δ*_C_	*δ*_H_	*δ*_C_	*δ*_H_	*δ*_C_
1	a 2.90 (dd, 15.0, 8.4) b 1.41 (dd, 15.0, 9.6)	38.3	a 2.23 (dd, 15.0, 10.8) b 1.65 (dd, 15.0, 5.4)	45.0	a 2.13 (m) b 1.61 (dd, 15.0, 5.4)	45.8
2	2.35 (m)	39.4	2.55 (m)	37.0	2.67 (m)	37.4
3	5.26 (dd, 7.2, 2.4)	81.6	5.83 (dd, 5.4, 4.2)	79.2	5.81 (t, 5.4)	78.6
4	2.64 (dd, 12.0, 7.2)	42.0	2.10 (dd, 12.0, 5.4)	49.7	2.40 (dd, 12.0, 5.4)	49.8
5	2.39 (dd, 12.0, 8.4)	35.7	2.63 (dd, 12.0, 8.4)	37.0	2.72 (dd, 12.0, 8.4)	38.0
6	—	39.0	—	46.0	—	46.6
7	6.45 (dd, 10.2, 2.4)	153.2	—	203.9	—	203.9
8	5.86 (d, 10.2)	125.3	5.86 (d, 10.2)	125.9	5.76 (d, 10.2)	126.3
9	—	204.6	6.43 (dd, 10.2, 1.8)	156.5	6.54 (dd, 10.2, 1.8)	156.9
10	—	43.8	—	34.6	—	35.2
11	2.16 (m)	54.9	2.31 (dd, 11.4, 1.8)	53.4	2.51 (dd, 11.4, 1.8)	53.6
12	1.90 (m)	36.2	2.34 (dd, 11.4, 7.8)	41.3	2.30 (dd, 11.4, 7.8)	42.2
13	1.87 (m)	36.6	2.19 (m)	34.1	2.22 (m)	34.8
14	5.62 (d, 1.8)	72.2	4.98 (d, 7.2)	78.2	4.97 (d, 7.2)	78.9
15	—	89.4	—	82.3	—	82.1
16	1.26 (d, 7.2)	20.1	1.10 (d, 7.2)	16.3	1.04 (d, 7.2)	16.7
17	1.37 s	22.0	1.29 (s)	21.0	1.27 (s)	20.9
18	1.07 s	27.9	1.15 (s)	30.7	1.16 (s)	30.8
19	0.98 s	21.7	1.02 (s)	25.7	1.05 (s)	25.8
20	0.96 (d, 7.2)	21.3	1.05 (d, 7.2)	17.5	1.06 (d, 7.2)	17.9
7′	—	166.1	—	165.5	—	166.5
1′	—	130.4	—	129.9	—	131.7
2′, 6′	8.05 (dd, 7.8, 1.2)	129.6	8.01 (dd, 7.8, 1.2)	129.7	8.12 (dd, 7.8, 1.2)	130.5
3′, 5′	7.47 (t, 7.8)	128.7	7.45 (t, 7.8)	128.8	7.50 (t, 7.8)	129.3
4′	7.60 (t, 7.8)	133.3	7.57 (t, 7.8)	133.3	7.62 (t, 7.8)	133.6
1″	—	169.3	—	171.0	—	170.7
2″	2.14 (s)	21.6	2.16 (s)	21.2	2.07 (s)	21.0
1″′	—	170.1	—	—	—	—
2″′	2.01 (s)	22.5	—	—	—	—
15-OH	—	—	—	—	3.23 (s)	—

^*a*^Data were recorded in CDCl_3_. ^*b*^Data were recorded in acetone-*d*_6_

resonances were attributed to five methyls (two secondary and three tertiary methyls by ^1^H NMR), one methylenes, ten methines (two olefinic methine and two oxygenated methines), and four quaternary carbons (two tetrasubstituted carbons, one oxygenated carbon, and one carbonyl carbon). Moreover, the above functionalities accounted for 9 degrees of unsaturation, and the remaining 4 degrees of unsaturation required the tetracyclic ring system of **1**, implying that heliosterpenoid A (**1**) likely possessed a new backbone. The 2D NMR experiments were thus performed to elucidate the diterpene skeleton and the positions of three acyloxy groups.

**Figure 2:**
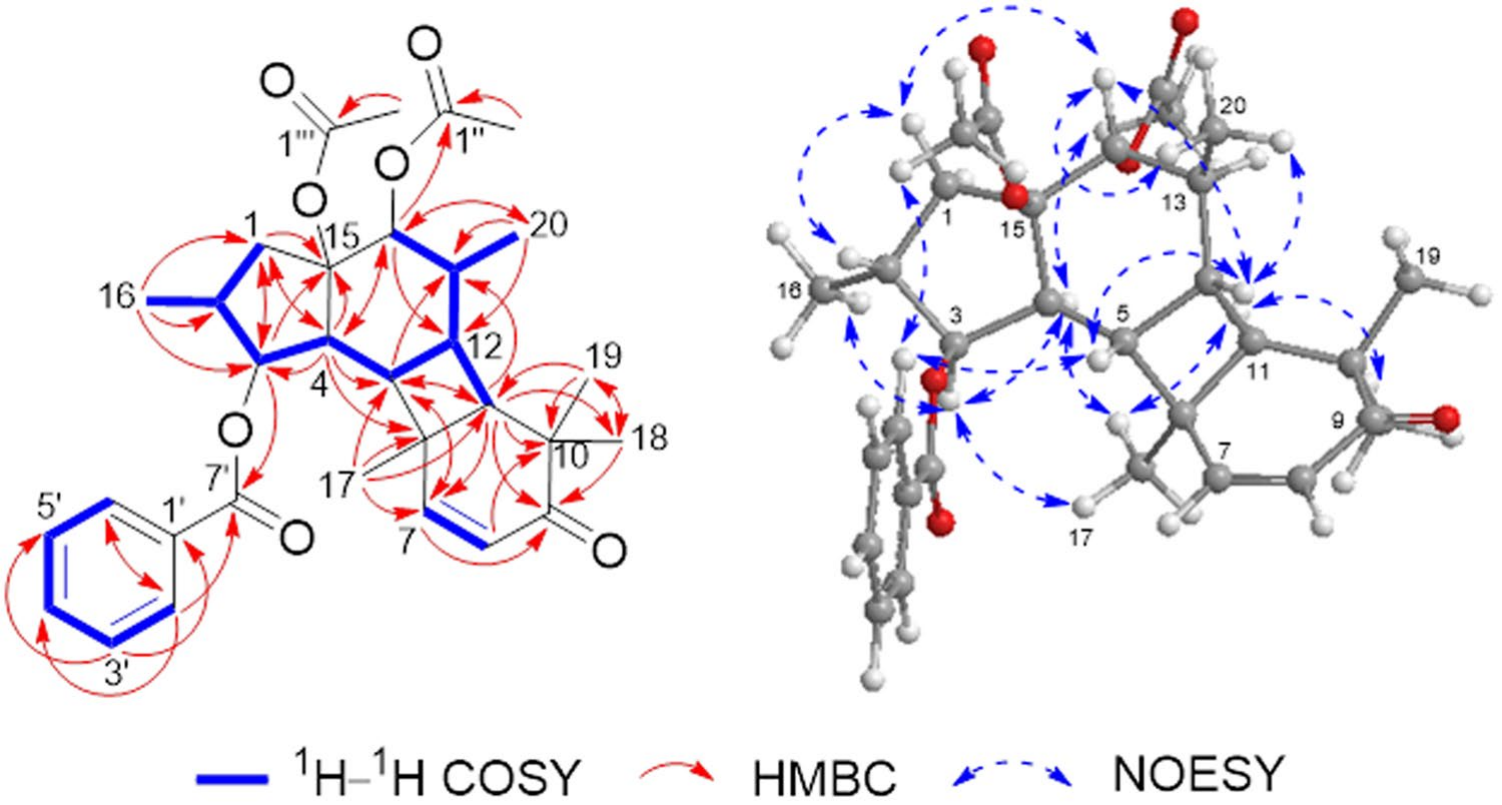
Key ^1^H–^1^H COSY, HMBC and NOESY correlations for 1.

HSQC data allowed the assignment of all the protons to their bonding carbons. Originally, detailed analyses of the HMBC and ^1^H-^1^H COSY spectra also led to the assignment of the general structure of the jatrophane skeleton. In the HMBC spectrum of **1** (Fig. 2), the correlations from H-1 to C-3, C-4, and C-15; from H_3_-16 to C-1, C-2, and C-3; from H-3 to C-1, C-15, and C-7′; from H-4 to C-1, C-3, C-5, C-6, C-14, and C-15 constructed a five-membered carbon ring that was fused to the macro-ring *via* C-4 and C-15 units, which was substituted with a methyl group (CH_3_-16) at C-2. By the corresponding HMBC correlations, the three oxygenated carbons *δ*_C_ 81.6, 72.2, and 89.4 were also assigned to C-3, C-14, and C-15, respectively. In addition, the key HMBC correlations from H-14 to C-1″, C-4, C-12, and C-20; from H_3_-20 to C-12, C-13, and C-14; from H-11 to C-9, C-10, C-13, and C-18; from H_3_-18 and H_3_-19 to C-9, C-10, and C-11; from H-7 to C-5 and C-9; from H_3_-17 to C-5, C-6, and C-7; from H-5 to C-3 and C-7, demonstrated the moiety of 12-membered ring, which was substituted with four methyl groups at C-6, C-10 × 2, and C-13, a double bond at C-7 and C-8 and a keto-carbonyl at C-9. Notably, the distinctive HBMC correlations from H_3_-17 to C-11; from H-11 to C-5, and C-7; from H-5 to C-11 and C-13, coupled with the remaining 3 degrees of unsaturation, revealed that C-5 and C-6 connect to C-12 and C-11, respectively. That means a 12-membered macro-ring was divided into three rings (B, C, and D) as shown in Fig. 1. ^1^H-^1^H COSY correlations of H-1/H-2(CH_3_-16)/H-3/H-4/H-5/H-12/H-11 and H-12/H-13(CH_3_-20)/H-14 proved the key fragment (Fig. 2, blue thick lines). After defining the diterpene skeleton, diagnostic correlations from H-3 to C-7′; from H-14 to C-1″ indicated the presence of the benzoyloxy group at C-3 and one acetoxy group at C-14. At last, the remaining one acetoxy group could only be attached at C-15. Therefore, the planar structure of **1** was established as depicted in Fig. 1.

The relative configuration of **1**, as shown in Fig. 2, was deduced from the interpretation of the NOESY spectrum, together with comparison with the large variety of known jatrophane diterpenes, in which all the five membered ring is *trans*-fused with the 12-membered macro-ring forming the skeleton, and H-4 is *α*-oriented and C-15 substituted group is *β*-oriented based on a biogenetic point of view^5, 14–17^. Besides, the NOE difference experiments of **1** were also performed to give further data for the relative configuration determination (Supporting Information). As judged from its NOESY spectrum, the cross-peaks observed between H-3/H_3_-16, H-3/H-4, H-4/H_3_-2″, H-4/H_3_-17, H_3_-17/H-11, and H-11/H_3_-18 indicated that H-3, H-4, H-11, H_3_-16, H_3_-17, H_3_-18 and OAc-14 all possessed the same *α*-orientations. The NOE correlations from H-2′/H_3_-2″′, H-2′/H-5, H-5/H-12, H-12/H-14, H-14/H_3_-20, H-14/H-1*β* and H-1*β*/H-2 demonstrated that H-2, H-5, H-12, H-14, H_3_-20, OAc-15 and OBz-3 have the same *β*-orientations (Fig. 2). Accordingly, the relative configuration of **1** was assigned, in which the A/B *trans*-fused-ring and B/C/D *cis*-fused-ring systems were constructed.

**Figure 3:**
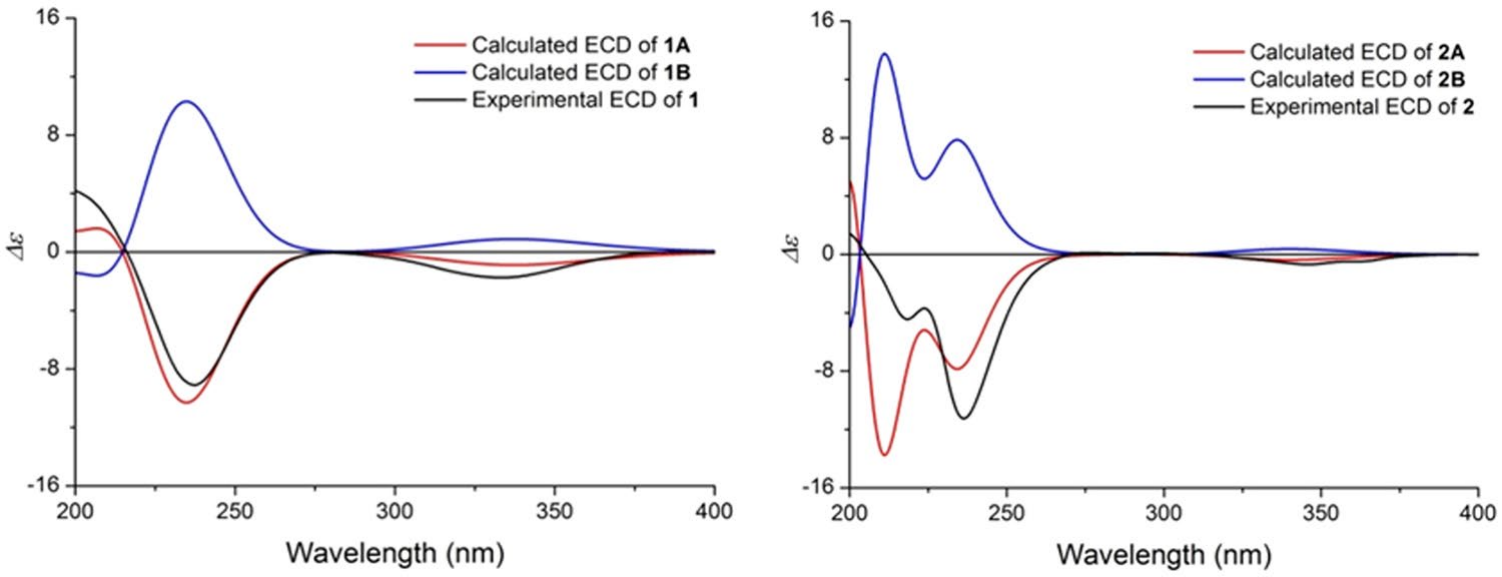
Experimental ECD spectrum of 1 and 2 and calculated ECD spectra of 1A, 1B, 2A and 2B in MeOH.

After initially unsuccessful repeated attempts to obtain single crystals of **1**, the absolute configuration of **1** was confirmed by using the classical octant rule of *α*,*β*-unsaturated ketones for electronic circular dichroism (ECD) data^18^. The ECD spectrum of **1** showed the negative Cotton effect at 333.5 nm, which resulted from the n → π* (R-bond) transition of the C = O group of *α*,*β*-unsaturated ketones in D ring. In the light of the optimized conformers (Supporting Information) and the value of C-9 at *δ*_C_ 204.6 in **1**, the *α*,*β*-unsaturated ketone is non-planar and there is a torsional angle between the two parts of the chromophore. Consequently, the positon of the double bonds determines the Cotton effect on the basis of the rule for the n → π* transition. So, the negative Cotton effect at 333.5 nm in the ECD spectrum of **1** suggested that the double bonds (C-7 and C-8) fall in the negative sectors, predicting the absolute configuration of **1** as shown in Fig. 1. The absolute configuration was also supported by comparison of the experimental ECD spectrum of **1** and calculated ECD spectra of **1 A** and **1B** predicted from time-dependent density functional theory calculations at the B3LYP/6-31 G(d) level. **1 A** and **1B** are an enantiomeric pair of **1**. And the experimental ECD spectrum of **1** indicated excellent agreement with the calculated ECD spectrum of **1 A** (Fig. 3). Therefore, the absolute configuration of **1** was determined as 2*R*, 3*S*, 4*S*, 5*S*, 6*S*, 7*Z*, 11*R*, 12*S*, 13*S*, 14*R*, 15*R*.

**Figure 4:**
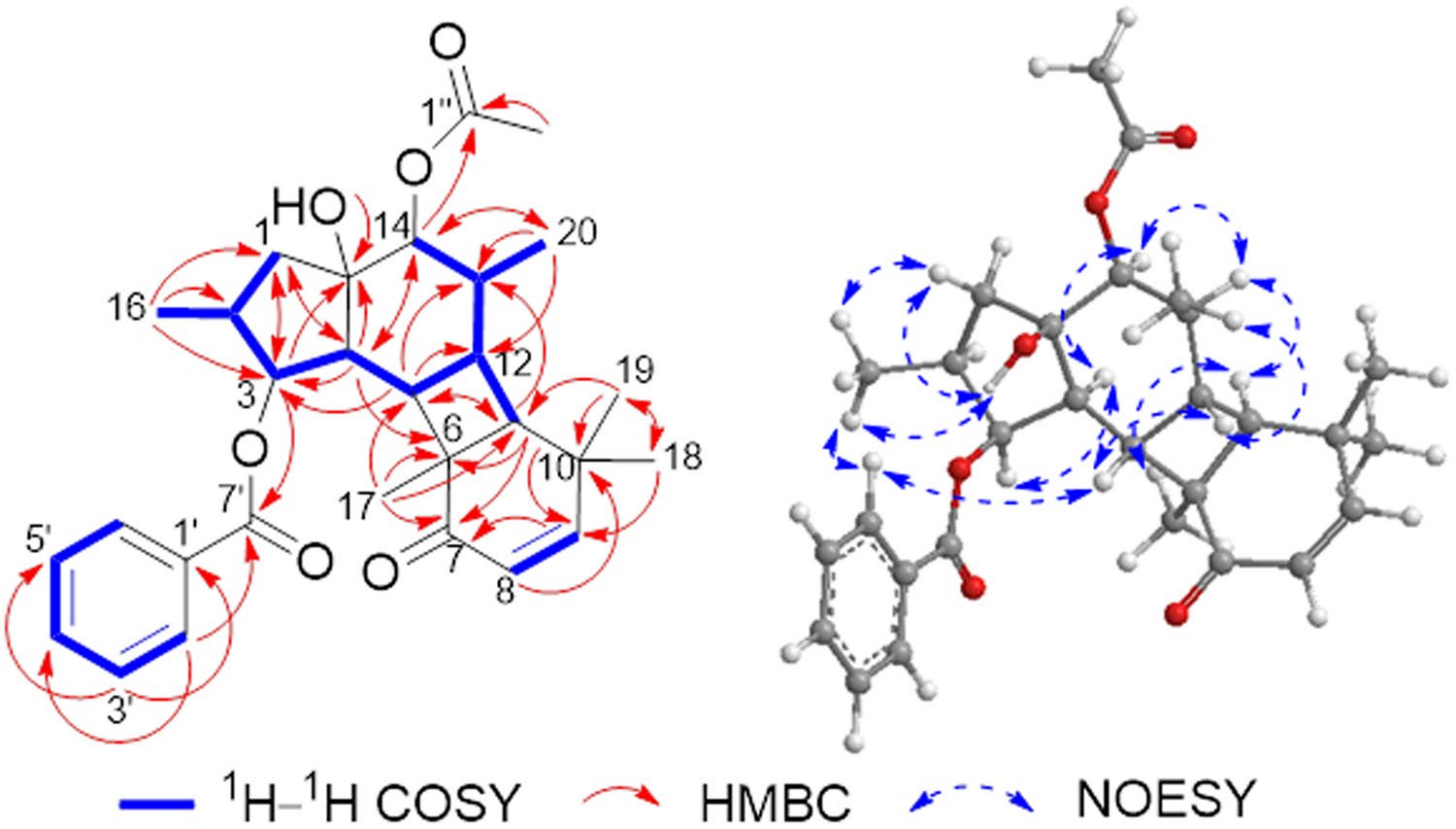
Key ^1^H–^1^H COSY, HMBC and NOESY correlations for 2.

Heliosterpenoid B (**2**) has a molecular formula of C_29_H_36_O_6_ as established by the HRESIMS ([M + Na]^+^ *m/z* 503.2423, calcd 503.2404), indicating 12 degrees of unsaturation. Its IR spectrum resembled those of **1**, except for an absorption band assignable to hydroxyl (3428 cm^−1^). The analyses of its ^1^H, ^13^C, HSQC, and ^1^H-^1^H COSY (Fig. 4, blue thick lines) NMR data suggested that **2** had the same carbon skeleton with **1**, except for the absence of an acetoxy group, which is replaced by a hydroxyl group. In the HMBC spectrum of **2** (Fig. 4), correlations from H_3_-17 to C-7; from H-8 to C-6 and C-10; from H-9 to C-7 and C-11; from H_3_-18 and H_3_-19 to C-9 revealed the presence of a double bond at C-8 and C-9 and a keto-carbonyl at C-7, which were different from **1**. HMBC correlations from OH-15 to C-14 indicated the presence of the hydroxyl group at C-15. The benzoyloxy group at C-3 and the acetoxy group at C-14 of **2** are identical to those of **1**. Notably, the major differences in the relative configuration of **2** in actone-*d*_6_ were that the NOESY correlations of H-2′/H_3_-16, H_3_-16/H-1*β*, H-1*β*/OH-15 as well as H-4/H-14 placed H_3_-16 and OH-15 on the same *β*-orientations and H-14 on the *α*-orientation (Fig. 4), whereas this configuration was very common in jatrophanes isolated from *Euphorbia helioscopia* ^5^. The ECD spectrum of **2** showed the negative Cotton effect at 347.5 nm. The absolute configuration of **2** was also confirmed by using the classical octant rule of *α*,*β*-unsaturated ketones for electronic circular dichroism (ECD) data and comparing of the experimental ECD spectrum of **2** and calculated ECD spectra of **2A** and **2B**. The experimental ECD spectrum of **2** indicated excellent agreement with the calculated ECD spectrum of **2A** (Fig. 3). Thus, the absolute configuration of **2** was determined as 2*S*, 3*S*, 4*S*, 5*S*, 6*S*, 8*Z*, 11*S*, 12*S*, 13*S*, 14*S*, 15*R*.

**Figure 5:**
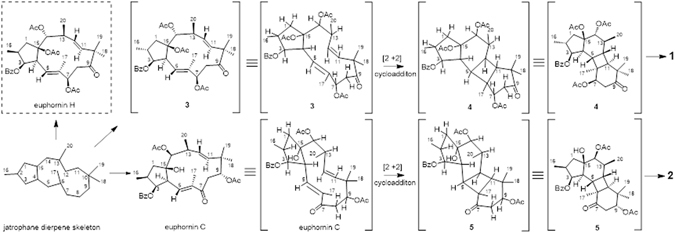
Plausible Biosynthetic Pathways of Heliosterpenoids A and B (1 and 2).

So far there have been some natural products that were isolated and originated by the [2 + 2] cycloaddition^19–22^, such as plumisclerin A and methyl sarcotroates A and B. Compounds **1** and **2** are two unprecedented jatrophane-derived diterpenoid esters, which have been identified as the first representative of diterpenoids featuring a novel 5/6/4/6-fused tetracyclic ring skeleton. The plausible biosynthetic pathways converting bicyclic jatrophanes to tertracyclic compound **1** and **2** were proposed in Fig. 5 ^19, 20^. **1** could be derived from the hypothetical precursor **3**, the 2-*epi*-isomer of euphornin H, which was also isolated from *E. helioscopia* ^5^. For compound **1**, an intramolecular [2 + 2] cycloaddition between 5,12- and 6,11- positions of **3** generated the intermediate **4**, which would then undergo a deacetylation reaction to afford **1**. Similarly, euphornin C, the hypothetical precursor of compound **2**, was converted into the cycloadduct (**5**), followed by a similar deacetylation reaction to produce **2**. Obviously, the configurations of the precursors are important and reasonable to propose the same absolute of configuration for the corresponding chirality centers of **1** and **2** based on the biogenetic consideration, especially the configuration of the cyclobutane ring formed.

**Figure 6:**
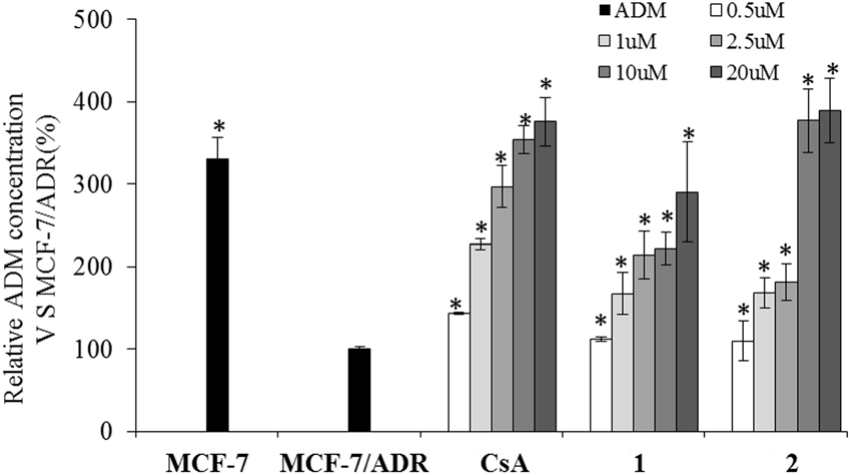
Concentration-dependent inhibition of Pgp-mediated ADM efflux by compounds 1 and 2 in MCF-7/ADR. Cyclosporin A (CsA) was used as a positive control. Compounds were tested at 0.5, 1.0, 2.5, 10 and 20 *μ*M, respectively. (*P < 0.001, significant differences of ADM concentration intracellular between treatment groups and DMSO vehicle control groups).

The P-glycoprotein (P-gp) inhibitory effects of compound **1** and **2** were tested using an adriamycin (ADM)-resistant human breast adenocarcinoma cell line (MCF-7/ADR). Cyclosporin A (CsA) was used as a positive control (IC_50_ = 0.49 *μ*M)^8^. **1** and **2** exhibited similar inhibitory activity compared to CsA, with IC_50_ values of 1.28 *μ*M and 1.02 *μ*M respectively (Fig. 6). Moreover, the cytotoxicity of compound **1** and **2** were also evaluated against five human cancer lines (MDA-MB-231, A549, Hela, U118MFG and RKO) and with adriamycin as the positive control (IC_50_ = 0.31 *μ*M) by MTT assay^9^. **1** exhibited modest cytotoxicity against MDA-MB-231 cell lines with IC_50_ value of 24.7 *μ*M. As the minor components, **1** and **2** provided a new structural template for potential inhibitors of P-glycoprotein (ABCB1) discovery.

## Materials and Methods

### General Experimental Procedures

Optical rotations were measured on a JASCO P-2000 automatic digital polarimeter. UV and CD spectra were recorded on a JASCO-815 CD spectrometer. IR spectra were measured on a Nicolet 5700 FT-IR microscope instrument (FT-IR microscope transmission). NMR spectra were obtained at 600 MHz for ^1^H and 150 MHz for ^13^C, respectively, on a Bruker AVIIIHD 600 NMR spectrometer. ESIMS and HRESIMS data were measured using AB SCIEX QTOF MS (QSTAR Elite) and Agilent 6520 Accurate-Mass Q-TOF LC/MS spectrometers, respectively. Analytical HPLC was conducted on an Agilent 1260 infinity system equipped with a DAD-UV detector. Preparative HPLC was performed on a CXTH system (Beijing Chuangxintongheng instrument Co. Ltd., P.R. China), equipped with a UV3000 detector using a YMC-Pack ODS-A column (250 × 20 mm, 5 *μ*m, Kyoto, Japan). Preparative TLC were performed on precoated TLC plates (400-500 *μ*m thickness, HSGF254, Yantai Jiangyou Silica Gel Development Co. Ltd., Yantai, P.R. China). Polyamide (30–60 mesh, Changzhou Changfeng Chemical Factory, China), Silica gel (100–200, 200–300 mesh, Qingdao Marin Chemical Inc. Qingdao, P.R. China) and Sephadex LH-20 (GE), and ODS (50 *μ*m, YMC, Japan) were used for column chromatography.

### Plant Material

The whole plants of *Euphorbia helioscopia* L. were purchased from Anguo Materia Medica Market in Hebei Province, China, in September 2015 and were identified by Professor Lin Ma, Institute of Materia Medica, Chinese Academy of Medical Scienes and Peking Union Medical College. A voucher specimen (ID-S-2624) has been deposited at the Herbarium of the Department of Medicinal Plants, the Institute of Materia Medica, Chinese Academy of Medical Sciences, Beijing.

### Extraction and Isolation

The dried and powdered whole plants of *Euphorbia helioscopia* (55 kg) were exhaustively extracted with 80% EtOH (3 × 140 L) under reflux. The EtOH extract was evaporated *in vacuo*, and the crude extract (8.5 kg) was diluted with H_2_O and partitioned successively with petroleum ether, EtOAc and *n*-BuOH (three times with 20 L each). To remove most of the chlorophylls and flavones, the EtOAc extract fraction (1.2 kg) was treated with polyamide in water, 50% EtOH and 95% EtOH, respectively. After removing the solvent, the 50% EtOH eluate (350 g) was chromatographed on a silica gel column and eluted a petroleum ether-acetone gradient system (30:1 to 1:1) to afford 18 fractions (A1-A18) on the basis of TLC analysis. A9 (4.7 g) was applied to RP-18 CC and eluted with MeOH-H_2_O (50:50 to 100:0) to obtain 27 fractions (A9-1 – A9-27). A9-23 (201.1 mg) was passed over Sephadex LH-20 (100% MeOH) to produce six subfractions (A9-23-1 – A9-23-6), and then A9-23-5 was separated by preparative HPLC (70% MeCN in H_2_O, 10 ml/min, wavelength at 230 nm) to yield A9-23-5-a. A9-23-5-a, an inseparable mixture was further purified by silica gel preparative TLC using petroleum ether-acetone (5:1) as the mobile phase to yield **1** (R_f_ = 0.5). Finally, **1** was subjected to Sephadex LH-20 (100% MeOH) again, to make **1** (1.0 mg) in a pure state. A11 (14.1 g) was fractionated over a column of MCI-gel eluted with a mixture of MeOH/H_2_O (7:3 to 9:1) to yield fractions m1-m4. Fraction m2 was separated on RP-18 CC (MeOH/H_2_O, 70 to 100%) to afford 12 fractions (A11-m2-1 – A11-m2-12). A11-m2-3 was purified by preparative HPLC to yield **2** (1.5 mg).

**Heliosterpenoid A (1)**. whiter amorphous powder; ${[{\rm{\alpha }}]}_{{\rm{D}}}^{20}$  + 82.1 (*c* 0.10, MeOH); IR (Nujol) *ν*_max_ 2963.1, 2928.9, 2875.1, 1743.0, 1715.3, 1675.2, 1453.7, 1373.0, 1277.5, 1233.2, 1115.4, 1027.1, 713.9 cm^−1^; CD (0.48 × 10^−3^ M, MeOH) λ_max_ (Δε) 237.5 (−9.10), 333.5 (−1.75) nm; ^1^H NMR (CDCl_3_, 600 MHz) and ^13^C NMR (CDCl_3_, 150 MHz) data, see Table 1; ( + )-ESIMS *m*/*z* 523.3 [M + H]^+^; ( + )-HRESIMS *m*/*z* 545.2518 ([M + Na]^+^, calcd for C_31_H_38_O_7_Na, 545.2510).

**Heliosterpenoid B (2)**. whiter amorphous powder; ${[{\rm{\alpha }}]}_{{\rm{D}}}^{20}$ + 41.3 (*c* 0.10, MeOH); IR (Nujol) *ν*_max_ 3427.8, 2965.8, 2931.5, 2875.2, 1717.4, 1660.6, 1453.0, 1375.4, 1249.0, 1123.0, 1044.8, 714.2 cm^−1^; CD (0.35 × 10^−3^ M, MeOH) λ_max_ (Δε) 236 (−7.66), 347.5 (−0.44) nm; ^1^H NMR (CDCl_3_, 600 MHz) and ^13^C NMR (CDCl_3_, 150 MHz) data, see Table 1; ( + )-ESIMS *m*/*z* 503.2 [M + Na]^+^; ( + )-HRESIMS *m*/*z* 503.2423 ([M + Na]^+^, calcd for C_29_H_36_O_6_Na, 503.2404).

## Electronic supplementary material

Supplementary Information
