# Supplementary material for: Heliosterpenoids A and B, two Novel Jatrophane-Derived Diterpenoids with a 5/6/4/6 Ring System from Euphorbia helioscopia
Source: Sci Rep. 2017 Jul 7;7:4922. doi: 10.1038/s41598-017-04399-w (PMC5501787; doi:10.1038/s41598-017-04399-w)
Supplement: Supplementary file 1 — Supplementary Information [file 41598_2017_4399_MOESM1_ESM.pdf]

Heliosterpenoids A and B, two Novel Jatrophane-Derived Diterpenoids with a 5/6/4/6 Ring System from *Euphorbia helioscopia*

Zhen-Peng Mai, Gang Ni, Yan-Fei Liu, Li Li, Guo-Ru Shi, Xin Wang, Jia-Yuan Li & De-Quan Yu*

State Key Laboratory of Bioactive Substance and Function of Natural Medicines, Institute of Materia Medica, Chinese Academy of Medical Sciences and Peking Union Medical College, Beijing 100050, P. R. China.

Correspondence author: dqyu@imm.ac.cn

Supporting information

Table of Contents

Experimental Section......S5
Spectral information of heliosterpenoid A (1)......S7
Figure S1. The optimized conformer of heliosterpenoid A (**1**) S7
Figure S2. Experimental ECD spectrum of **1** and calculated ECD spectra of **1A** and **1B** in MeOH S7
Figure S3. Experimental ECD spectrum of heliosterpenoid A (**1**) S8
Figure S4. UV spectrum of heliosterpenoid A (**1**)......S9
Figure S5. IR spectrum of heliosterpenoid A (**1**) S10
Figure S6. (+)-ESIMS data of heliosterpenoid A (**1**) S11
Figure S7. (+)-HRESIMS data of heliosterpenoid A (**1**)......S12
Figure S8. ^{1}H NMR spectrum of heliosterpenoid A (**1**) in $CDCl_3$ (600 MHz)......S13
Figure S9. ^{13}C NMR spectrum of heliosterpenoid A (**1**) in $CDCl_3$ (150 MHz)......S14
Figure S10. DEPT spectrum of heliosterpenoid A (**1**) in $CDCl_3$ (150 MHz) S15
Figure S11. HSQC spectrum of heliosterpenoid A (**1**) in $CDCl_3$ (^{1}H: 600 MHz, ^{13}C: 150 MHz)......S16
Figure S12. HMBC spectrum of heliosterpenoid A (**1**) in $CDCl_3$ (^{1}H: 600 MHz, ^{13}C: 150 MHz) S17
Figure S13. ^{1}H-^{1}H COSY spectrum of heliosterpenoid A (**1**) in $CDCl_3$ (600 MHz)......S18
Figure S14. NOESY spectrum of heliosterpenoid A (**1**) in $CDCl_3$ (600 MHz)......S19
Figure S15. NOE difference spectrum 1 of heliosterpenoid A (**1**) in $CDCl_3$ (600 MHz)......S20
Figure S16. NOE difference spectrum 2 of heliosterpenoid A (**1**) in $CDCl_3$ (600 MHz)......S21
Spectral information of heliosterpenoid B (2)......S22
Figure S17. The optimized conformer of heliosterpenoid B (**2**) S22
Figure S18. Experimental ECD spectrum of **2** and calculated ECD spectra of **2A** and **2B** in MeOH......S23
Figure S19. Experimental ECD spectrum of heliosterpenoid B (**2**)......S24
Figure S20. UV spectrum of heliosterpenoid B (**2**) S25

Figure S21. IR spectrum of heliosterpenoid B (**2**) S26
Figure S22. (+)-ESIMS data of heliosterpenoid B (**2**) S27
Figure S23. (+)-HRESIMS data of heliosterpenoid B (**2**)........ S28
Figure S24. ^{1}H NMR spectrum of heliosterpenoid B (**2**) in $CDCl_3$ (600 MHz) S29
Figure S25. ^{13}C NMR spectrum of heliosterpenoid B (**2**) in $CDCl_3$ (150 MHz) S30
Figure S26. DEPT spectrum of heliosterpenoid B (**2**) in $CDCl_3$ (150 MHz)........ S31
Figure S27. HSQC spectrum of heliosterpenoid B (**2**) in $CDCl_3$ (^{1}H: 600 MHz, ^{13}C: 150 MHz) S32
Figure S28. HMBC spectrum of heliosterpenoid B (**2**) in $CDCl_3$ (^{1}H: 600 MHz, ^{13}C: 150 MHz) S33
Figure S29. ^{1}H-^{1}H COSY spectrum of heliosterpenoid B (**2**) in $CDCl_3$ (600 MHz)........ S34
Figure S30. NOESY spectrum of heliosterpenoid B (**2**) in $CDCl_3$ (600 MHz)........ S35
Figure S31. ^{1}H NMR spectrum of heliosterpenoid B (**2**) in acetone-d_6 (600 MHz) S36
Figure S32. ^{13}C NMR spectrum of heliosterpenoid B (**2**) in acetone-d_6 (150 MHz) S37
Figure S33. HSQC spectrum of heliosterpenoid B (**2**) in acetone-d_6 (^{1}H: 600 MHz, ^{13}C: 150 MHz)........ S38
Figure S34. HMBC spectrum of heliosterpenoid B (**2**) in acetone-d_6 (^{1}H: 600 MHz, ^{13}C: 150 MHz)........ S39
Figure S35. ^{1}H-^{1}H COSY spectrum of heliosterpenoid B (**2**) in acetone-d_6 (600 MHz) S40
Figure S36. NOESY spectrum of heliosterpenoid B (**2**) in acetone-d_6 (600 MHz) S41
Figure S37. NOE difference spectrum 1 of heliosterpenoid B (**2**) in acetone-d_6 (600 MHz)........ S42
Figure S38. NOE difference spectrum 2 of heliosterpenoid B (**2**) in acetone-d_6 (600 MHz) S43
Spectral inforamtion of euphornin C........ S44
Figure S39. ^{1}H NMR spectrum of euphornin C in $CDCl_3$ (500 MHz)........ S44
Figure S40. ^{13}C NMR spectrum of euphornin C in $CDCl_3$ (500 MHz)........ S45
Figure S41. HSQC spectrum of euphornin C in $CDCl_3$ (500 MHz)........ S46
Figure S42. HMBC spectrum of euphornin C in $CDCl_3$ (500 MHz)........ S47
Figure S43. ^{1}H-^{1}H COSY spectrum of euphornin C in $CDCl_3$ (500 MHz)........ S48
Figure S44. NOESY spectrum of euphornin C in $CDCl_3$ (500 MHz)........ S49
Spectral information of euphornin H........ S50

Figure S45. ^{1}H NMR spectrum of euphornin H in $CDCl_3$ (500 MHz)……………………………………………………………………………..S50
Figure S46. ^{13}C NMR spectrum of euphornin H in $CDCl_3$ (500 MHz)…………………………………………………………………………..….S51
Figure S47. HSQC spectrum of euphornin H in $CDCl_3$ (500 MHz)…………………………………………………………………………………...….S52
Figure S48. HMBC spectrum of euphornin H in $CDCl_3$ (500 MHz)…………………………………………………………………………………..…S53
Figure S49. ^{1}H-^{1}H COSY spectrum of euphornin H in $CDCl_3$ (500 MHz)…………………………………………………………..……...…S54
Figure S50. NOESY spectrum of euphornin H in $CDCl_3$ (500 MHz)……………………………………………………………………………….…S55
LC-MS extracted ion chromatograms (EIC) of heliosterpenoids A and B (1 and 2) in crude extract of *Euphorbia helioscopia*……………………………….S56
Figure S51. TIC and DAD spectra of compound **1** in LC-MS……………………………………………………………………………………..S56
Figure S52. TIC and DAD spectra of compound **2** in LC-MS…………………………………………………..…………………………………S57
Figure S53. TIC and DAD spectra of the crude extract in LC-MS………………………………………………………...…………………………S58
Figure S54. EIC spectra of compounds **1** and **2** in the crude extract by LC-MS……………………………………………………………..………S59
Figure S55 EIC spectra of compounds **1** (*m/z* 545.2483) and **2** (*m/z* 481.2605) in the crude extract by LC-MS………………………………………S60

Experimental Section

Biological Assay

Intracellular Adriamycin Accumulation Assay. Human breast adenocarcinoma cells (MCF-7) and Adriamycin-resistant MCF-7 (MCF-7/ADR) cells were seeded in a 24-well plate at a density of 1×10^5 cells/well and incubated for 48 h at 37 ℃ in a 95% relative humidity atmosphere containing 5% CO_2. After preincubation with fresh medium containing either the commonly used P-gp inhibitor Cyclosporin A (CsA) (0.5-20 μM) or compound **1** and **2** (0.5-20 μM) for 10 min, 10 μM adriamycin was added to the medium. The plates were incubated for 1h at 37 ℃ with gentle shaking. The reaction was terminated by removal of the medium. Cells were then washed three times with 1 mL of ice-cold PBS. The cell monolayers were subsequently lysed with 0.3 mL of 0.1% Triton X-100, and the concentration of adriamycin in the cell lysate was determined by LC-MS/MS. Protein concentrations served as the loading control and were measured using the bicinchoninic acid procedure with bovine serum albumin as the standard (Solarbio, China). MCF-7 cells were used as a positive control for maximum adriamycin accumulation.

Cytotoxicity Assay.

MDA-MB-231 (Human breast cancer cell line), A549 (Human lung adenocarcinoma), Hela (Human Cervical adenocarcinoma), U118MFG (human glioblastoma) and RKO (Human colorectal adenocarcinoma) cell lines were maintained in RPMI 1640 medium or DMEM medium containing 10% fetal bovine serum (FBS), 100 units/mL penicillin, and 100 μg/mL streptomycin sulfate. All cell cultures were maintained at 37 ℃ in a humidified atmosphere containing 5% CO_2. The cells (5×10^3) were seeded in 96-well plates, allowed to adhere for overnight to obtain 80% confluent monolayer. The cells were changed to fresh medium containing compound **1** and **2**. After incubation for 48h, cell viability was determined by measuring the metabolic conversion of 3-(4,5-dimethylthiazol-2-yl)-2,5-diphenyltetrazolium bromide (MTT) into purple formazan crystals by viable cells. The MTT assay results were read using an MK3 Wellscan plate reader at 570 nm. Compounds were tested at five concentrations (10^{-5}, 10^{-6}, 10^{-7}, 10^{-8}, 10^{-9} M) and were dissolved in 100% DMSO with a final concentration of DMSO of 0.1% (*v*/*v*) in each well. Adriamycin was used as a positive control. Each concentration of the compounds was tested in three parallels. IC_{50} values were calculated using Microsoft Excel software.

Computationla section

Conformational analyses of **1A** and **2A** were performed by using the MMFF94

molecular mechanics force field via the MOE software package. Enantiomer **1A** showed three conformers (Figure S1), and **2A** showed six conformers (Figure S17). The conformers were further optimized at the B3LYP/6-31g(d) level in methanol. The energies, oscillator strengths, and rotational strengths of the first 50 electronic excitations were calculated using the TDDFT methodology at the B3LYP/6-31g (d) level. ECD spectrums of the conformers were simulated using a Gaussian function with a half-bandwidth of 0.35eV. The corresponding theoretical ECD spectrum of the enantiomers **1B** and **2B** were depicted by inverting that of **1A** and **2A**, respectively. All quantum computations were performed using Gaussian 09 program package, on an IBM cluster machine located at the High Performance Computing Center of Peking Union Medical College.

LC-MS analysis of 1 and 2 in the crude extract

The dried and powdered whole plants of *Euphorbia helioscopia* (30 g) were extracted in 80% EtOH by ultrasound-assisted extraction under dark condition (3 × 2 h for each time). The crude extract dissolved in methanol and then filtrated in order to remove undissolved components. After concentrating under vacuum, the residue (3 g) was diluted to 1.0 mL with methanol, which was analyzed using a Agilent 6520 Q-TOF LC-MS (gradient: 0-10 min, 10-60% A; 10-30 min, 60-80% A; 30-40 min, 80-100% A (A: MeCN; B: H_2O)) with the flow rate of 1.0 mL/min. The YMC column used was a 250 × 4.6 mm, i.d., 5 μm, YMC-Pack ODS-A.

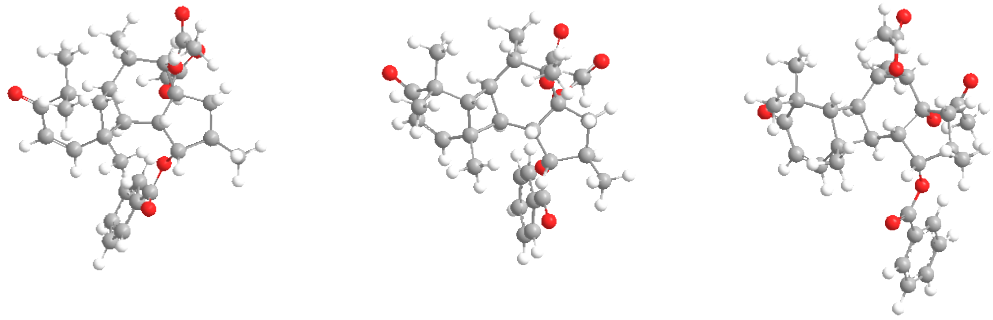

1AC1 (57.5%) **1AC2** (23.2%) **1AC3** (19.3%)

Figure S1. The optimized conformer of **1**.

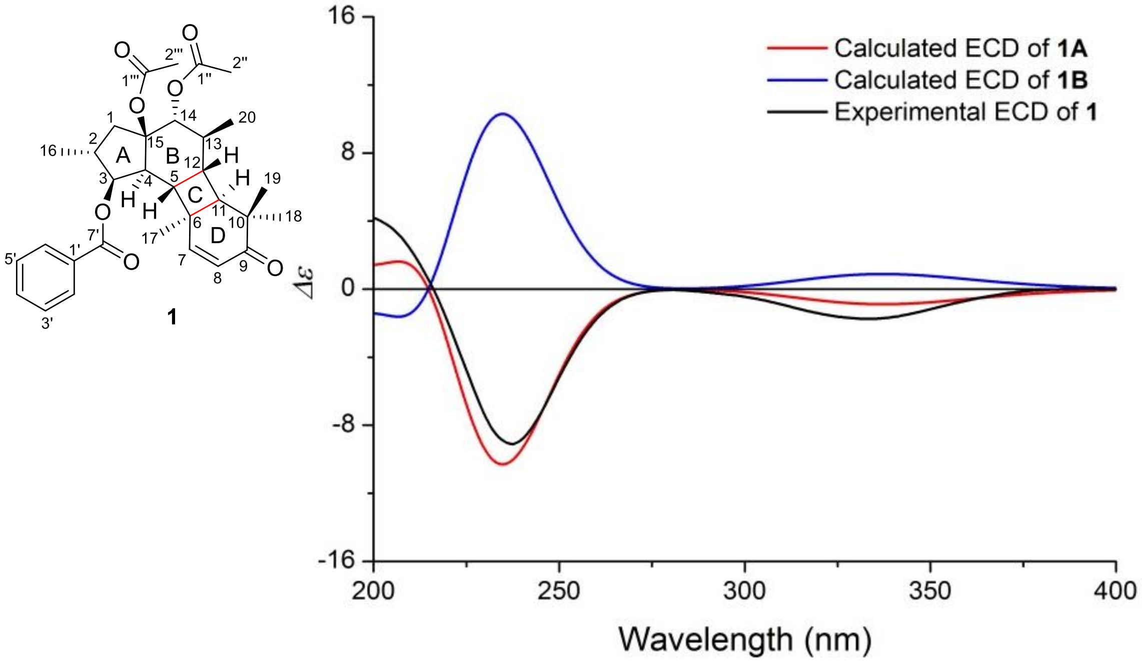

Figure S2. Experimental ECD spectrum of **1** and calculated ECD spectra of **1A** and **1B** in MeOH.

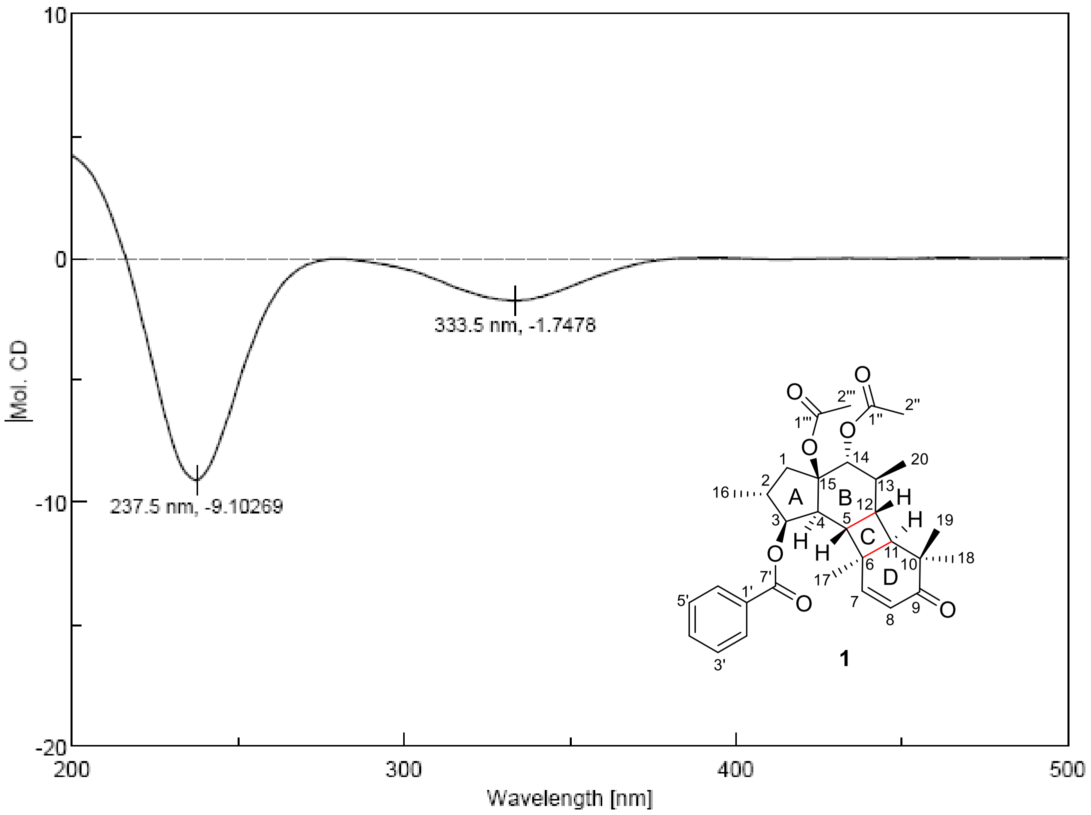

Figure S3. Experimental ECD spectrum of heliosterpenoid A (**1**)

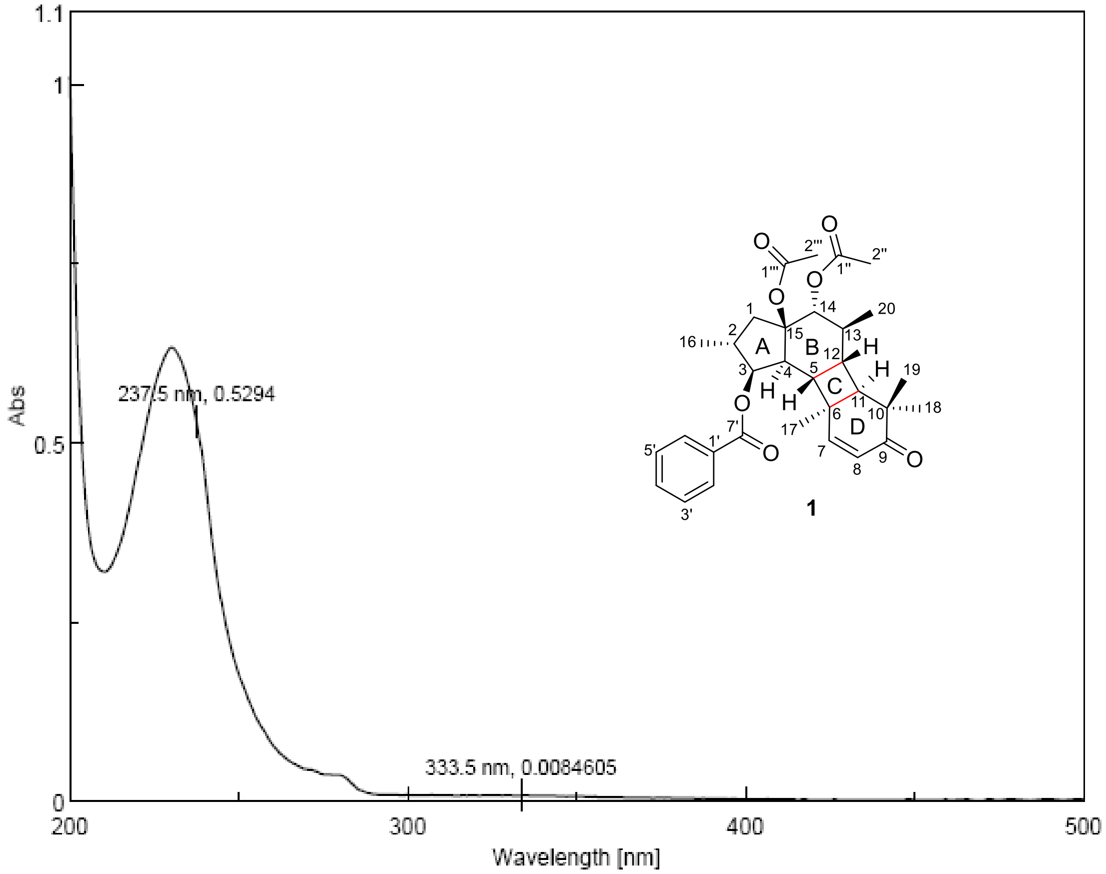

Figure S4. UV spectrum of heliosterpenoid A (**1**)

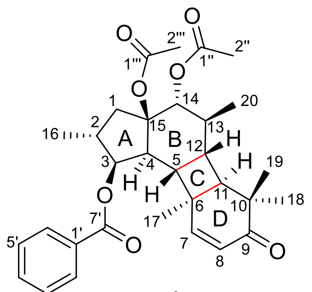

1

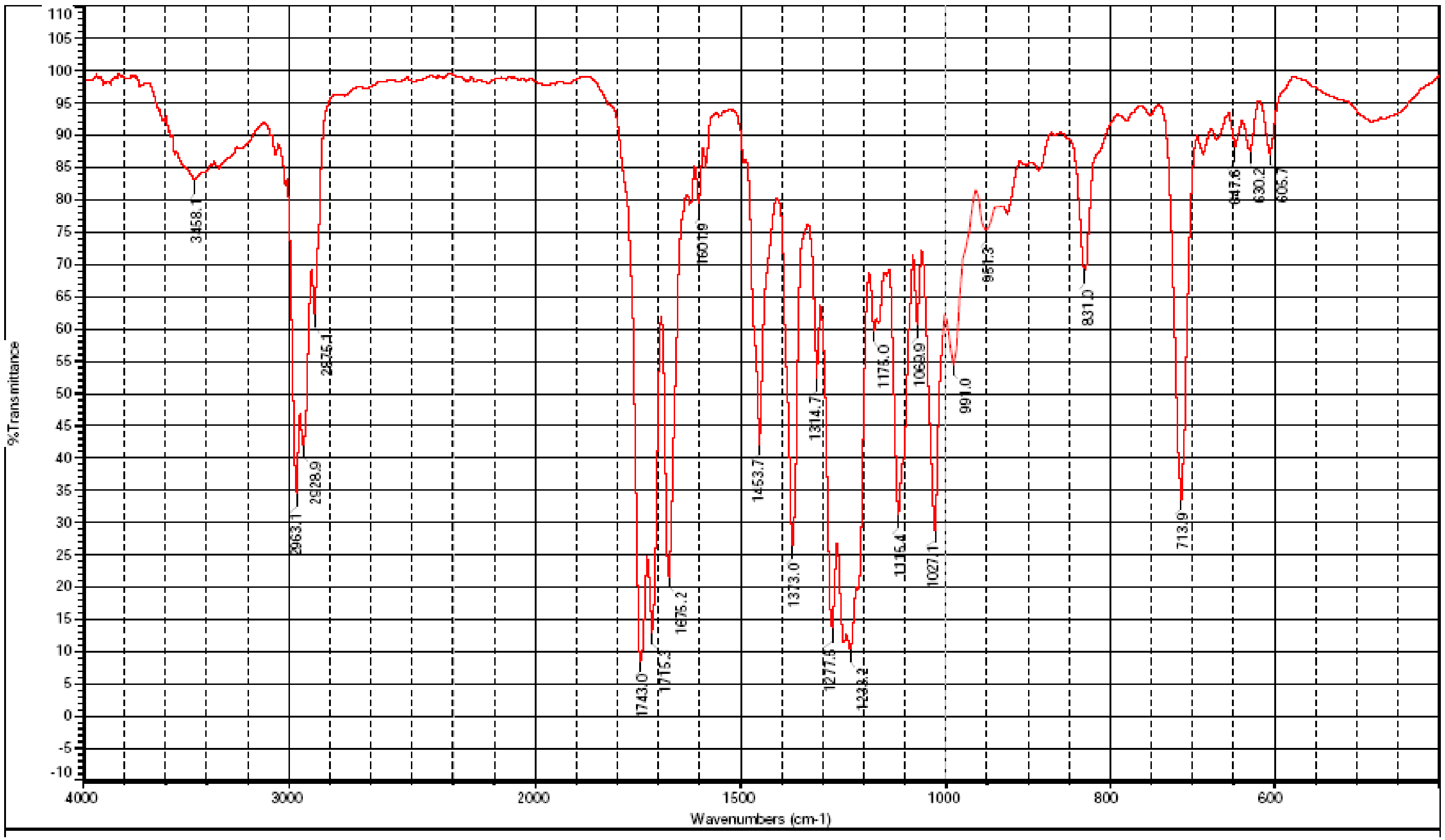

Figure S5. IR spectrum of heliosterpenoid A (**1**)

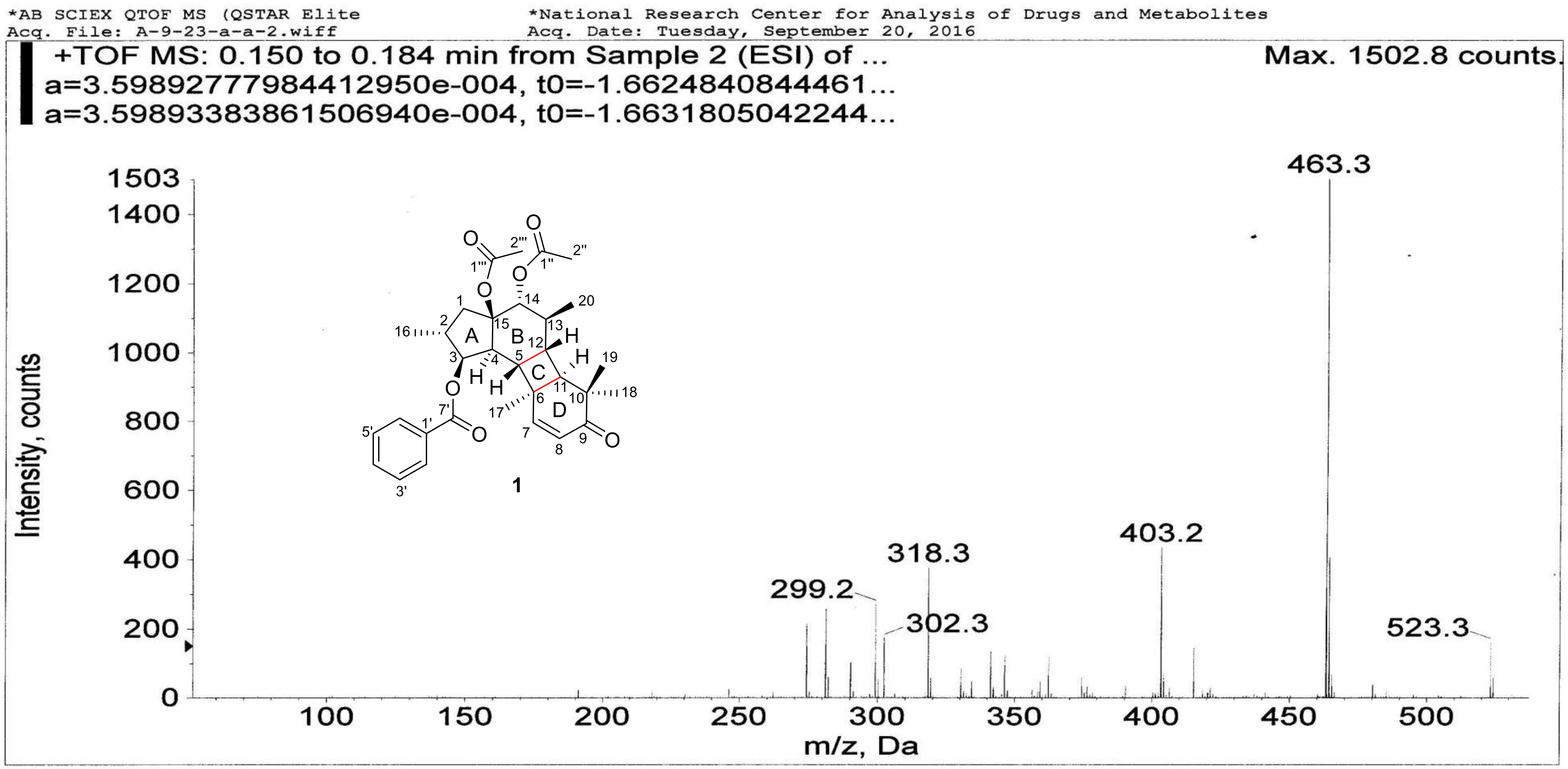

Figure S6. (+)-ESIMS data of heliosterpenoid A (**1**)

MS Formula Results: + Scan (8.359 min) Sub (2016091401.d)

m/z	Ion	Formula	Abundance
545.2518	(M+Na)+	C31 H38 Na O7	426790.9

Best	Formula (M)	Ion Formula	Score	Cross Sco	Mass	Calc Mass	Calc m/z	Diff (ppm)	Abs Diff (ppm)	Mass Match	Abund Match	Spacing Match	DBE
✔	C31 H38 O7	C31 H38 Na O7	99.87		522.2626	522.2618	545.251	-1.57	1.57	99.92	99.83	99.81	13
	C28 H42 O7 S	C28 H42 Na O7 S	99.03		522.2626	522.2651	545.2543	4.88	4.88	99.25	97.99	99.85	8
	C23 H42 N2 O9 S	C23 H42 N2 Na O9 S	98.56		522.2626	522.2611	545.2503	-2.84	2.84	99.74	95.54	99.81	4
	C32 H42 O2 S2	C32 H42 Na O2 S2	97.93		522.2626	522.2626	545.2518	0.08	0.08	100	93	99.7	12
	C19 H42 N2 O14	C19 H42 N2 Na O14	97.51		522.2626	522.2636	545.2528	1.96	1.96	99.88	91.56	99.92	0
	C24 H46 N2 O4 S3	C24 H46 N2 Na O4 S3	95.28		522.2626	522.262	545.2512	-1.19	1.19	99.96	84.09	99.37	3

Figure S7. (+)-HRESIMS data of heliosterpenoid A (**1**)

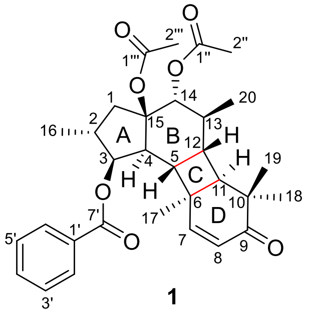

1

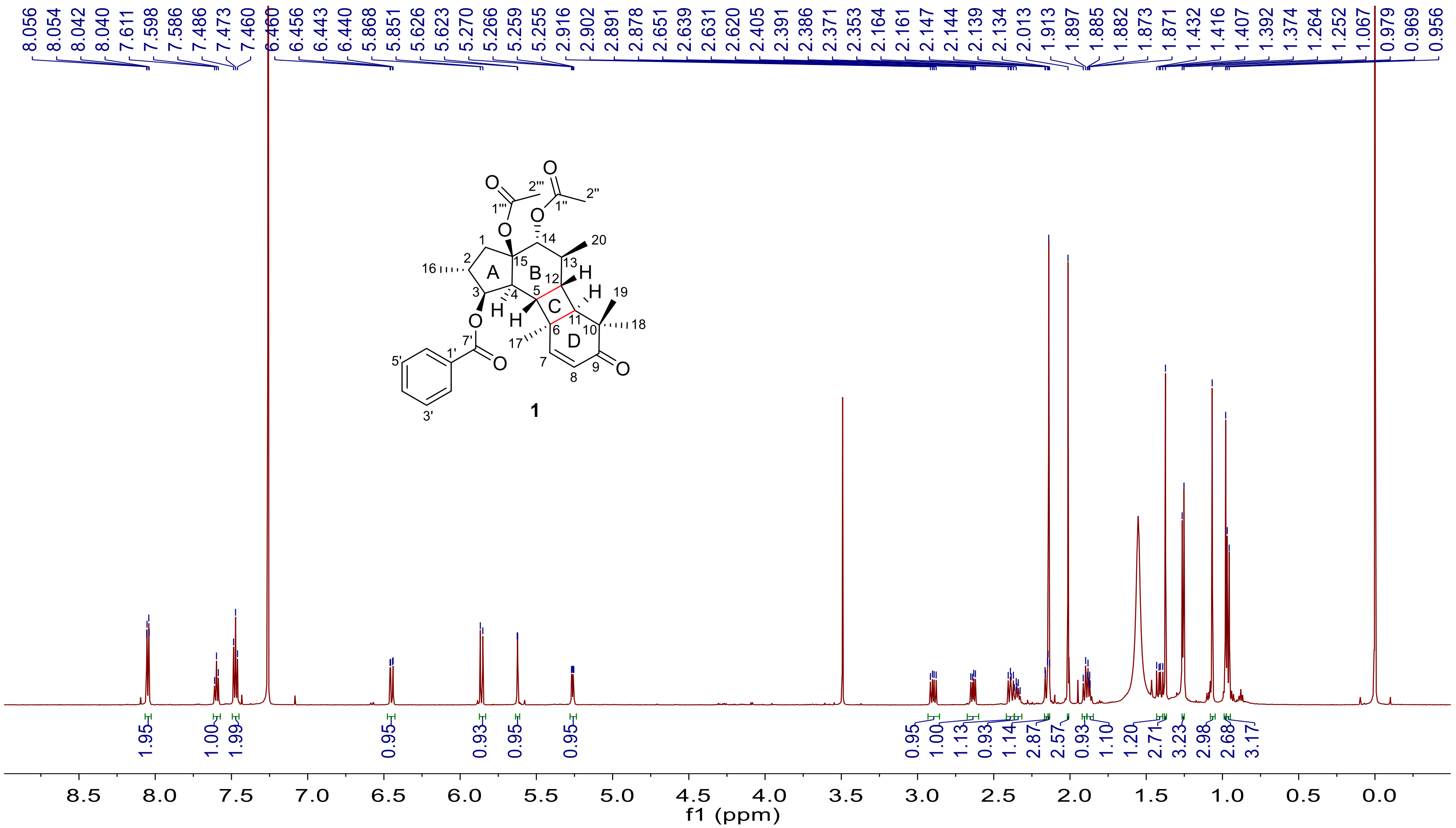

Figure S8. ^{1}H NMR spectrum of heliosterpenoid A (**1**) in CDCl3 (600 MHz)

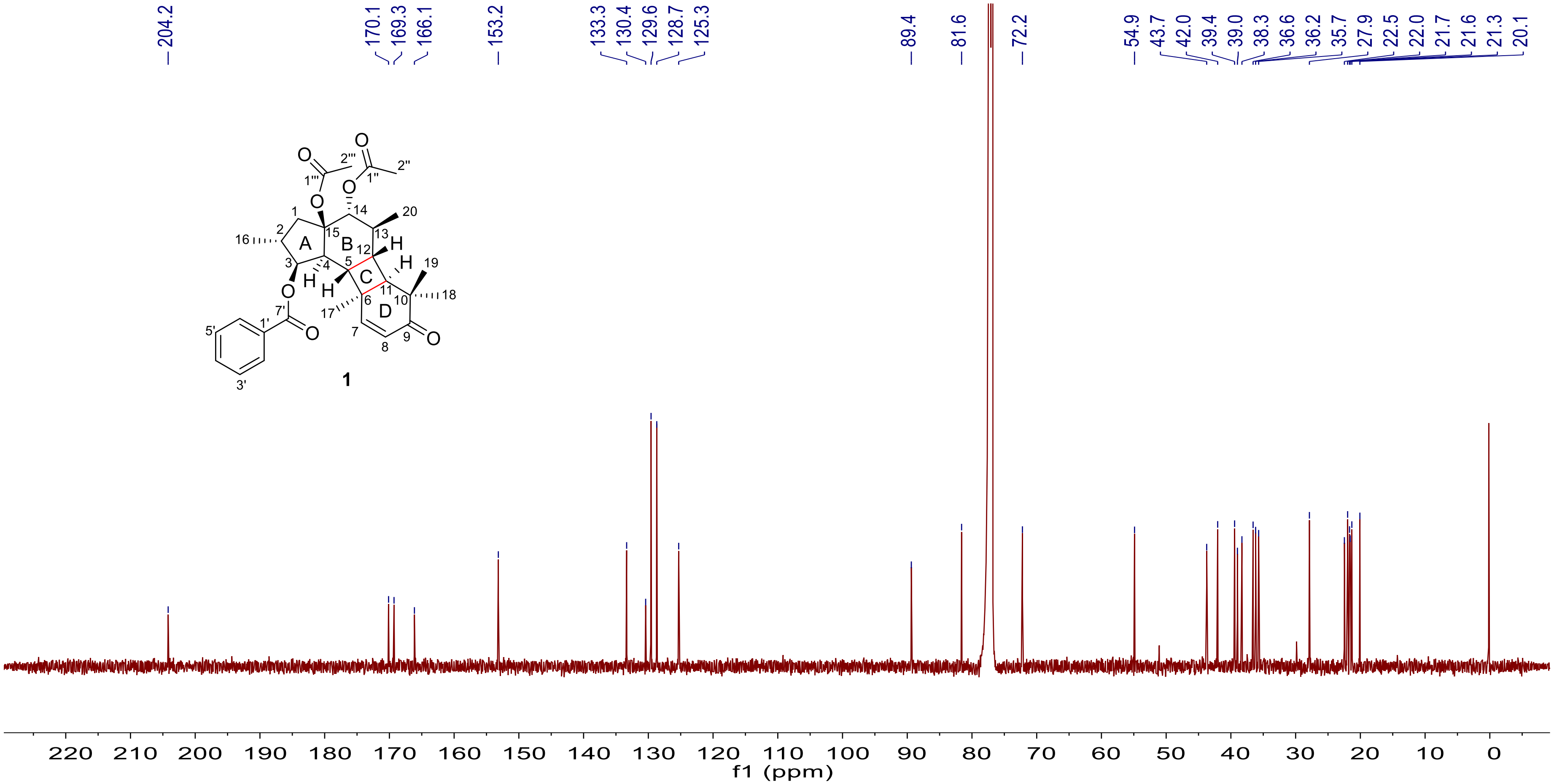

Figure S9. ^{13}C NMR spectrum of heliosterpenoid A (**1**) in $CDCl_3$ (150 MHz)

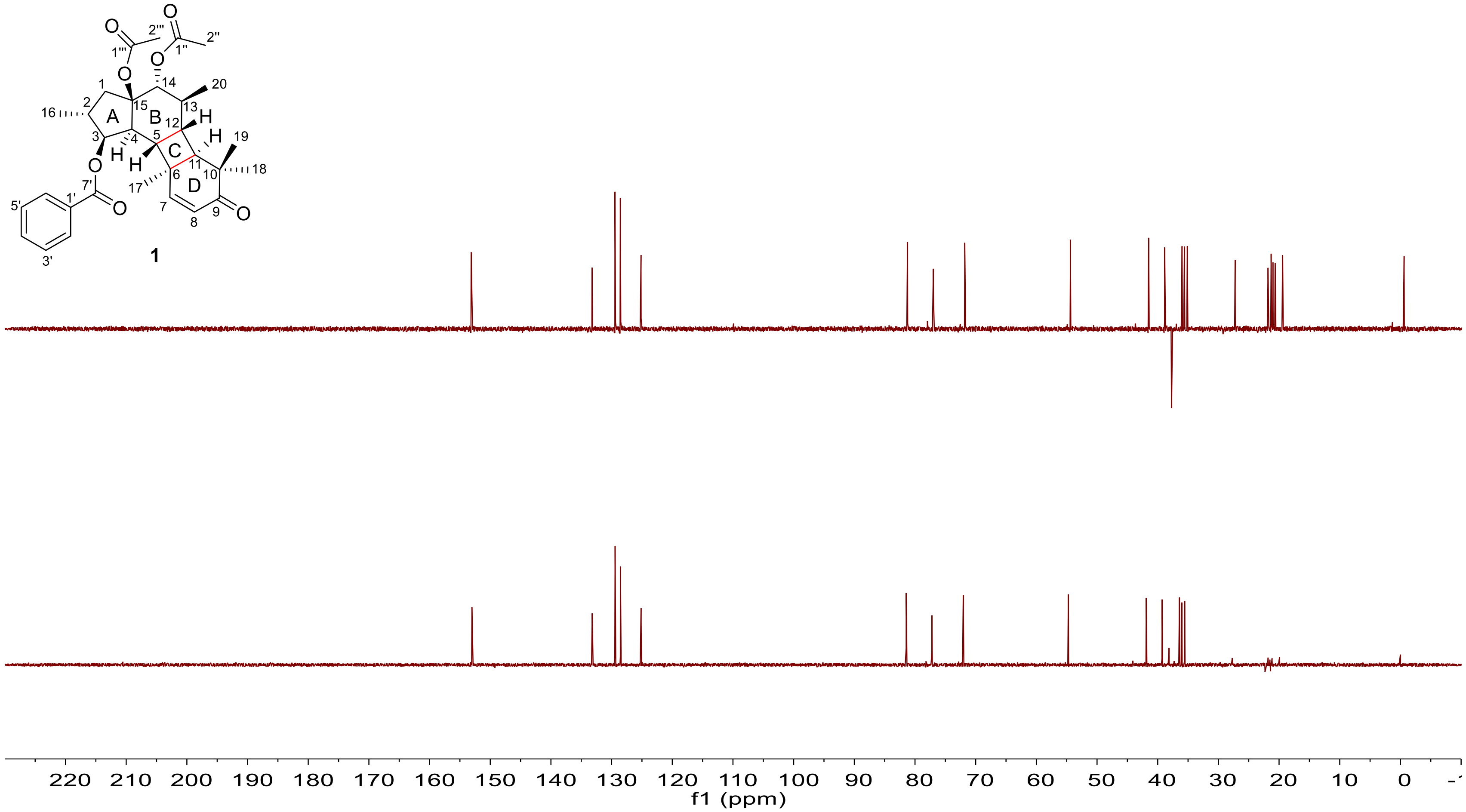

Figure S10. DEPT spectrum of heliosterpenoid A (**1**) in $CDCl_3$ (150 MHz)

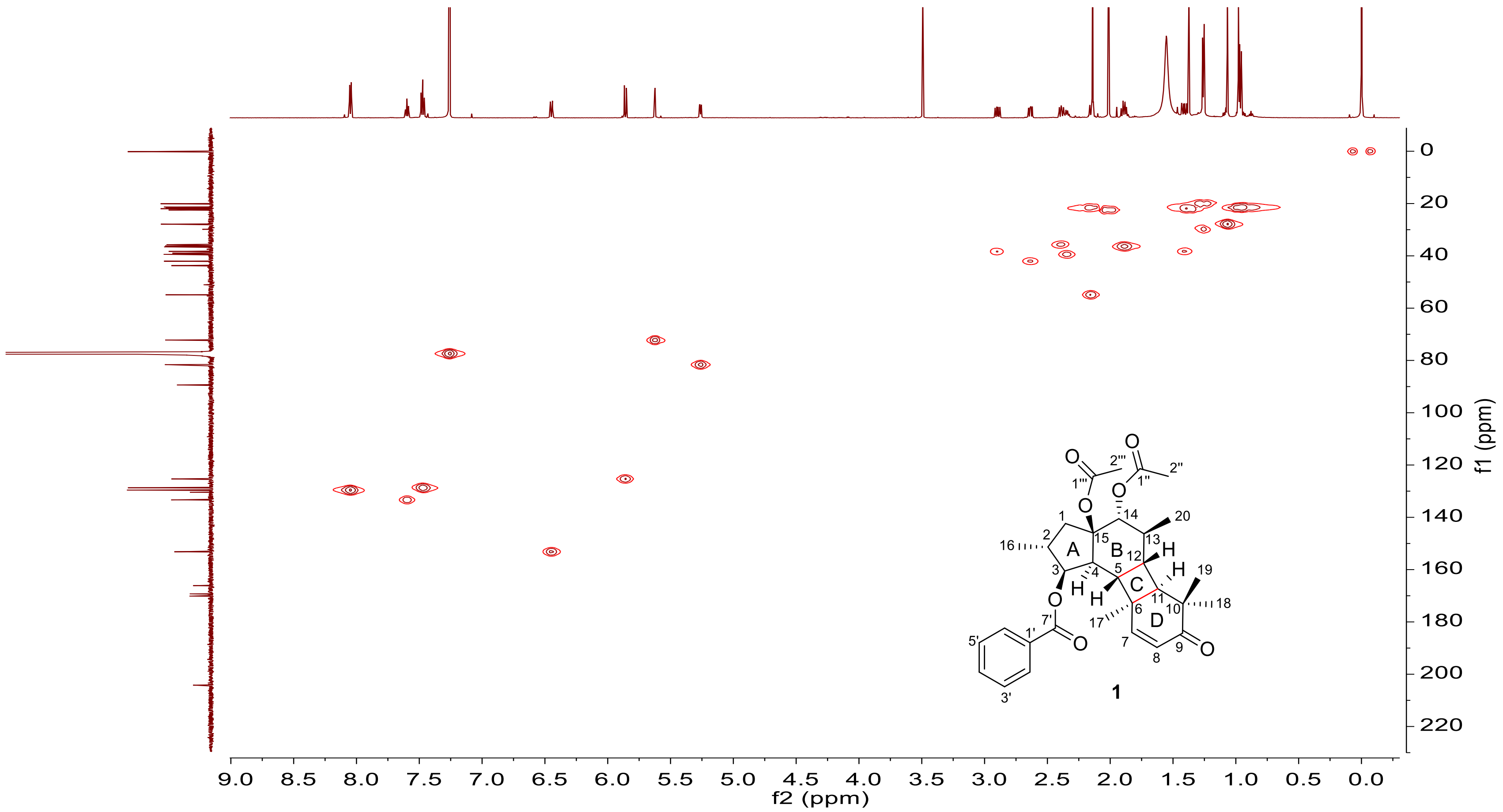

Figure S11. HSQC spectrum of heliosterpenoid A (**1**) in $CDCl_3$ (^{1}H: 600 MHz, ^{13}C: 150 MHz)

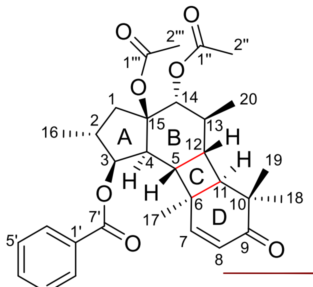

1

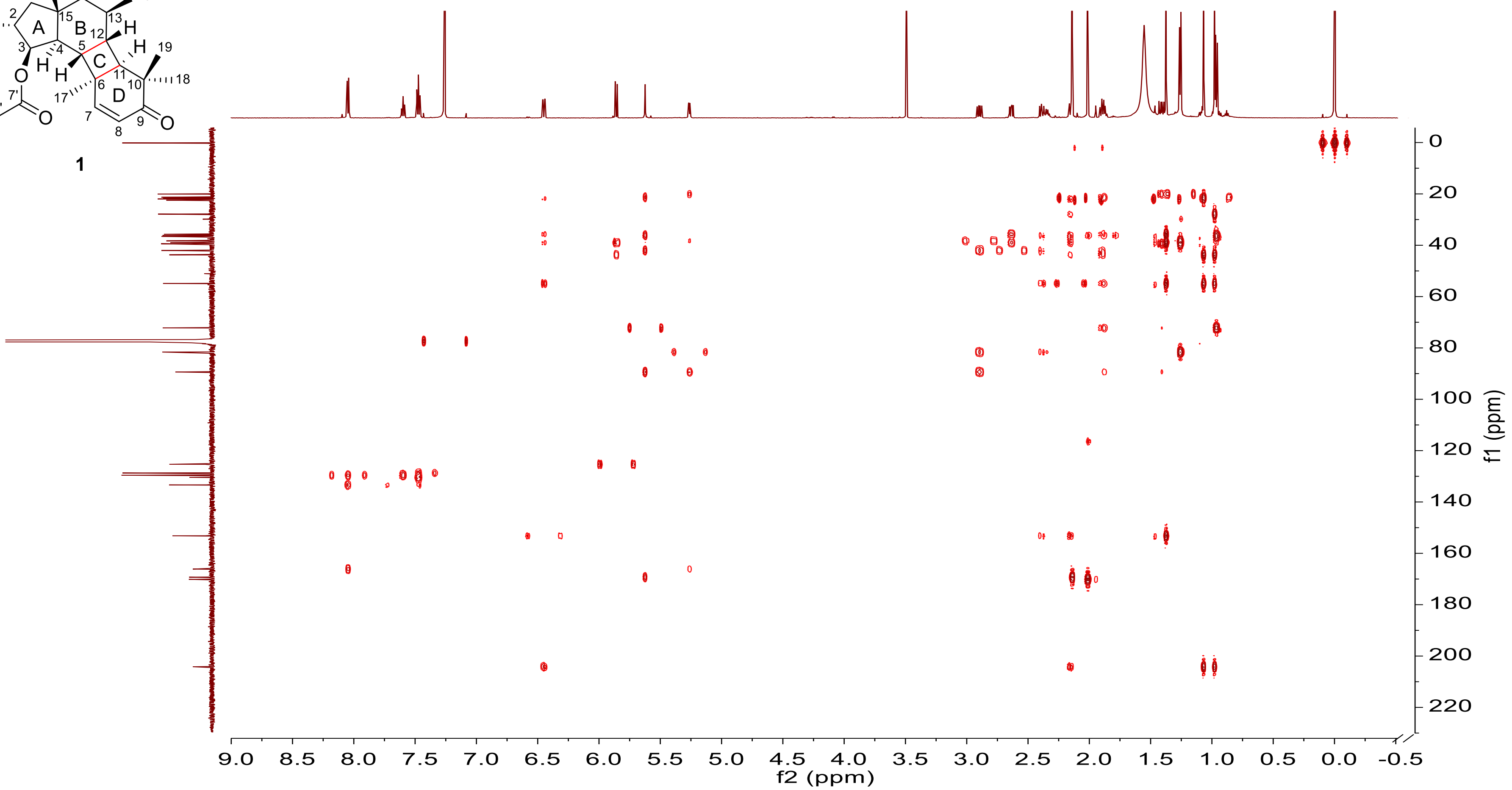

Figure S12. HMBC spectrum of heliosterpenoid A (**1**) in $CDCl_3$ (^1H: 600 MHz, ^{13}C: 150 MHz)

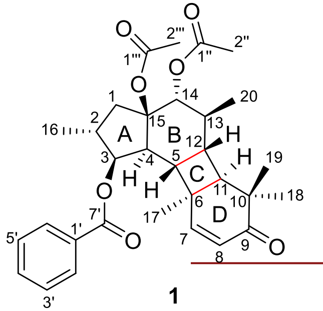

1

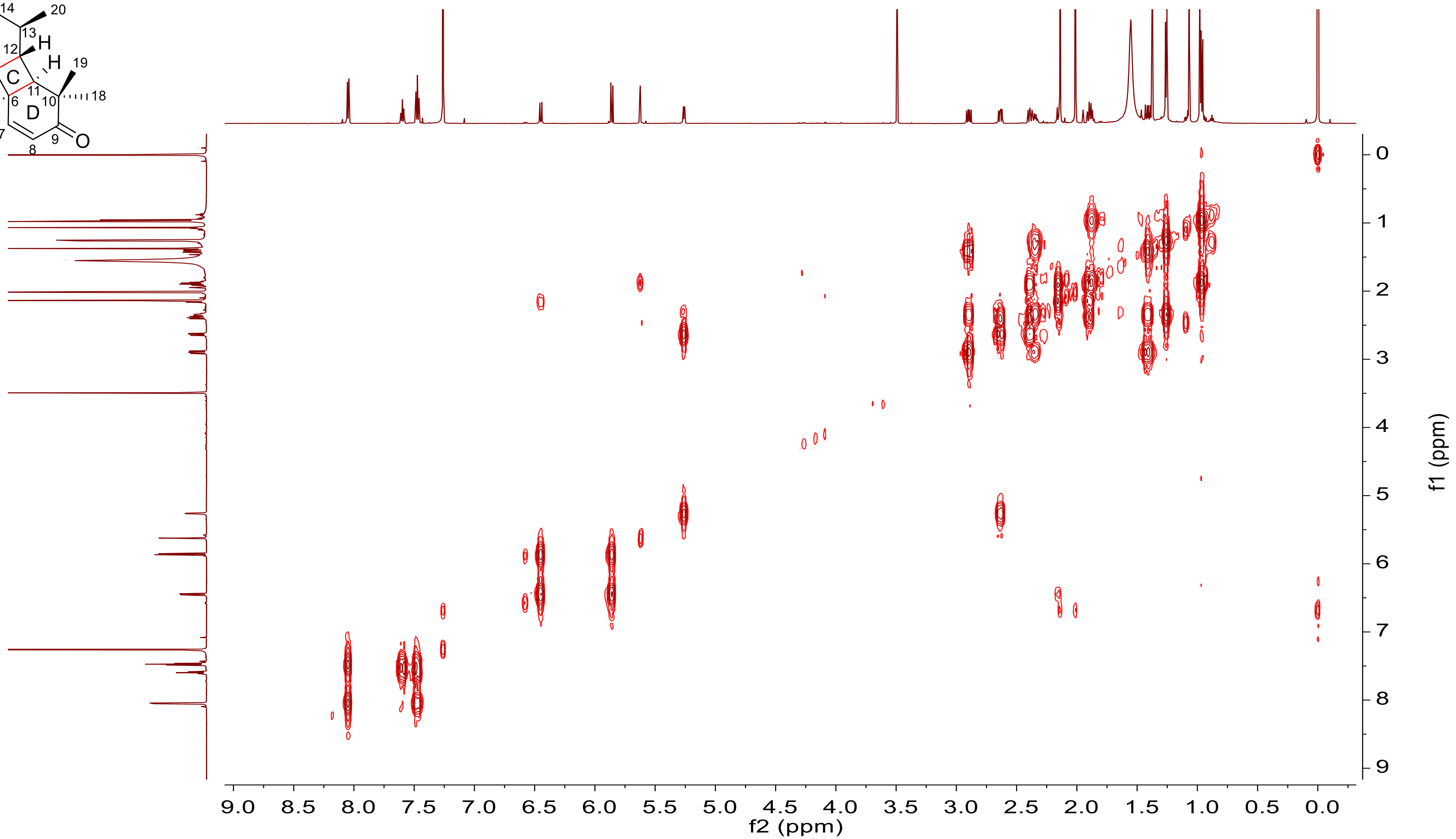

Figure S13. ^{1}H-^{1}H COSY spectrum of heliosterpenoid A (**1**) in $CDCl_3$ (600 MHz)

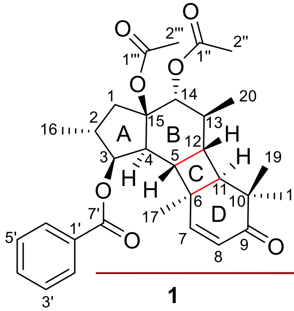

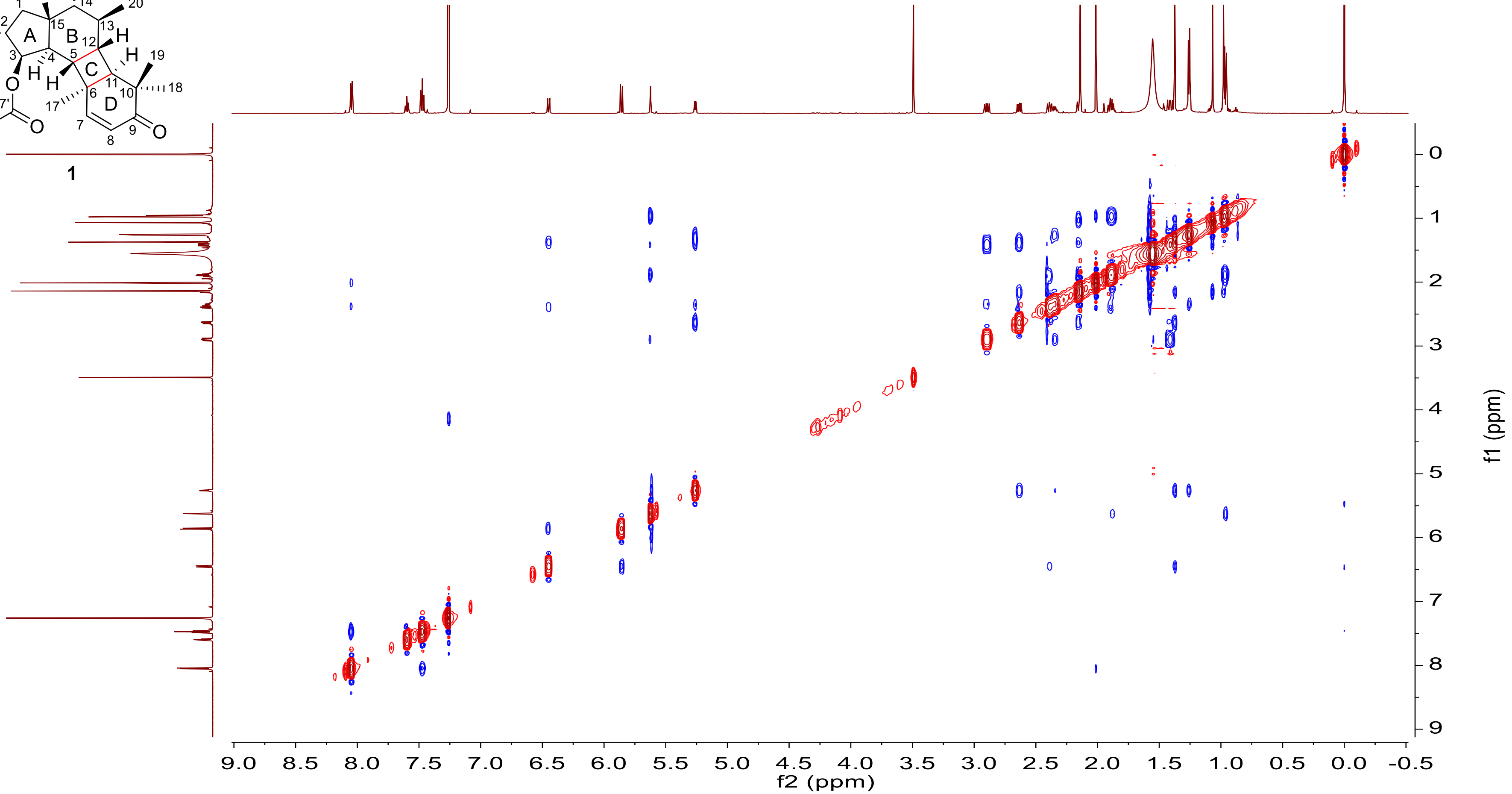

Figure S14. NOESY spectrum of heliosterpenoid A (**1**) in $CDCl_3$ (600 MHz)

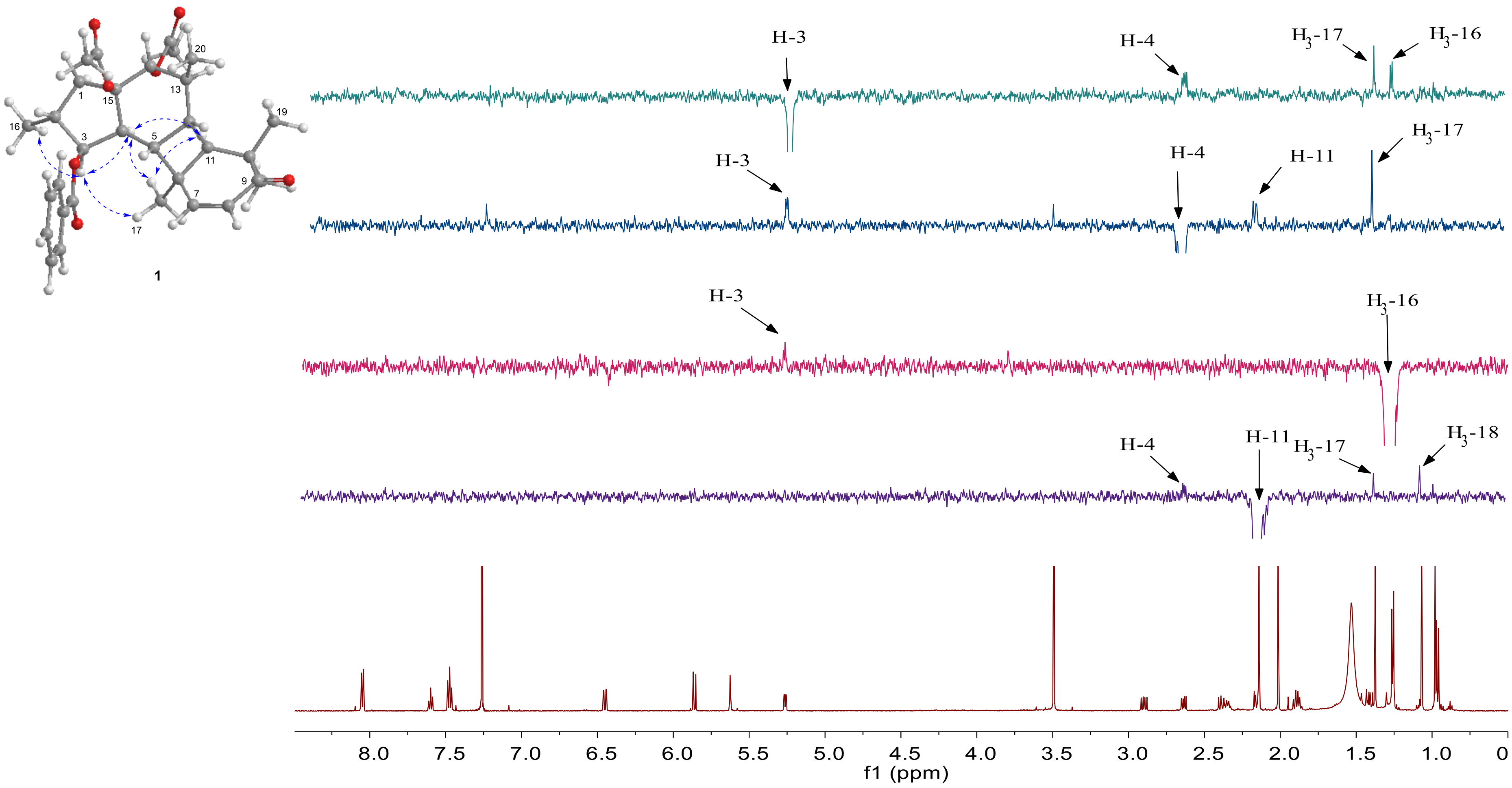

Figure S15. NOE difference spectrum 1 of heliosterpenoid A (**1**) in $CDCl_3$ (600 MHz)

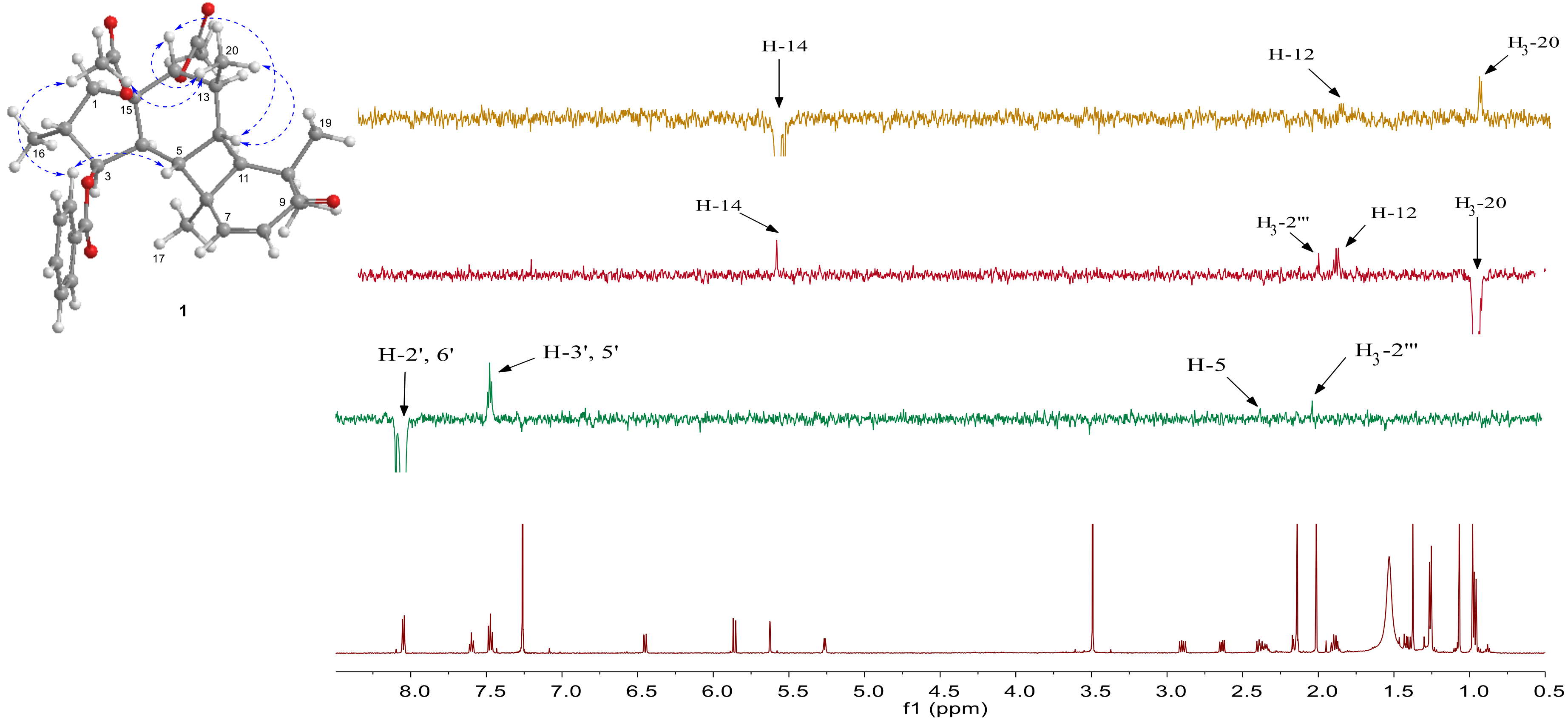

Figure S16. NOE difference spectrum 2 of heliosterpenoid A (**1**) in $CDCl_3$ (600 MHz)

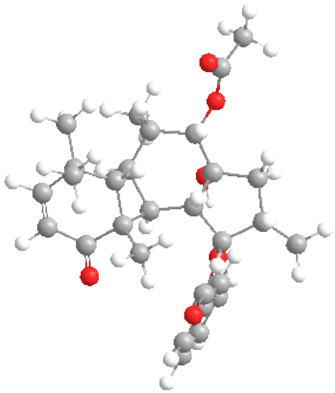

2AC1 (30.5%)

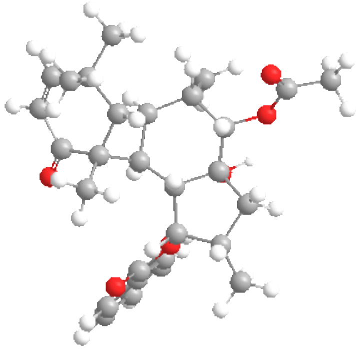

2AC2 (22.7%)

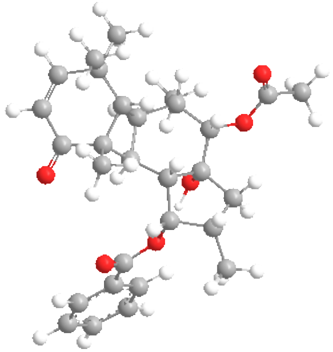

2AC3 (17.2%)

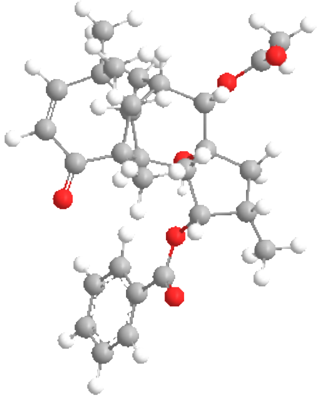

2AC4 (16.2%)

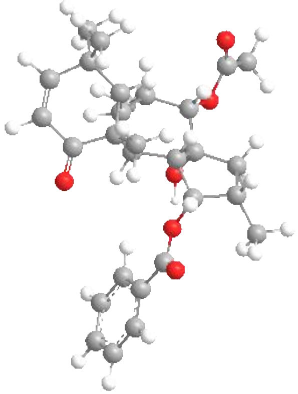

2AC5 (13.3%)

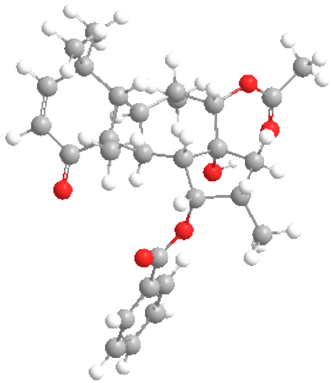

2AC6 (0.1%)

Figure S17. The optimized conformer of heliosterpenoid B (**2**)

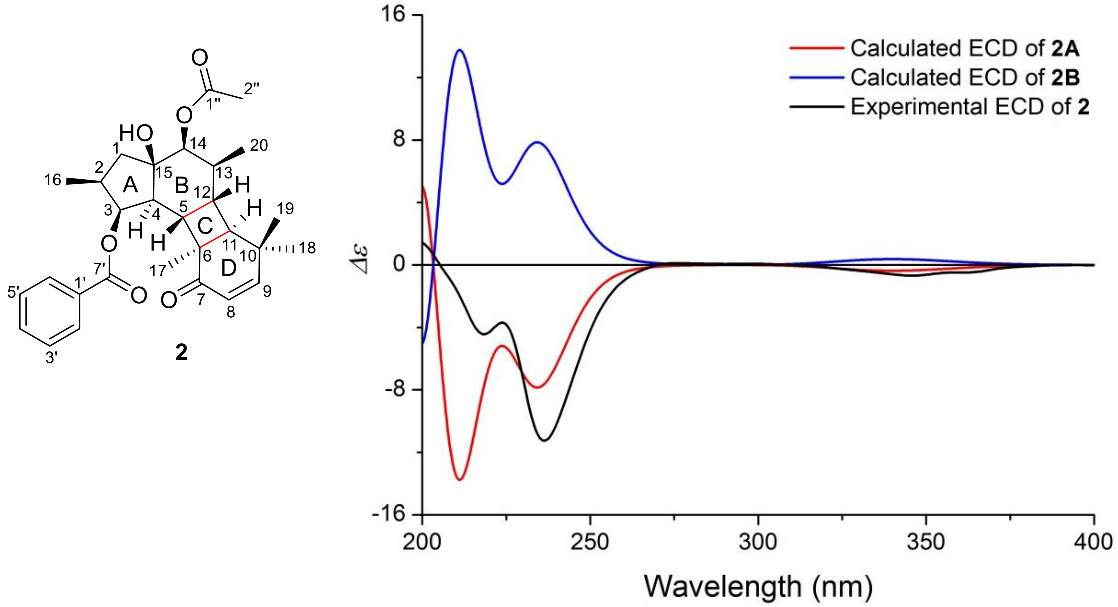

Figure S18. Experimental ECD spectrum of **2** and calculated ECD spectra of **2A** and **2B** in MeOH

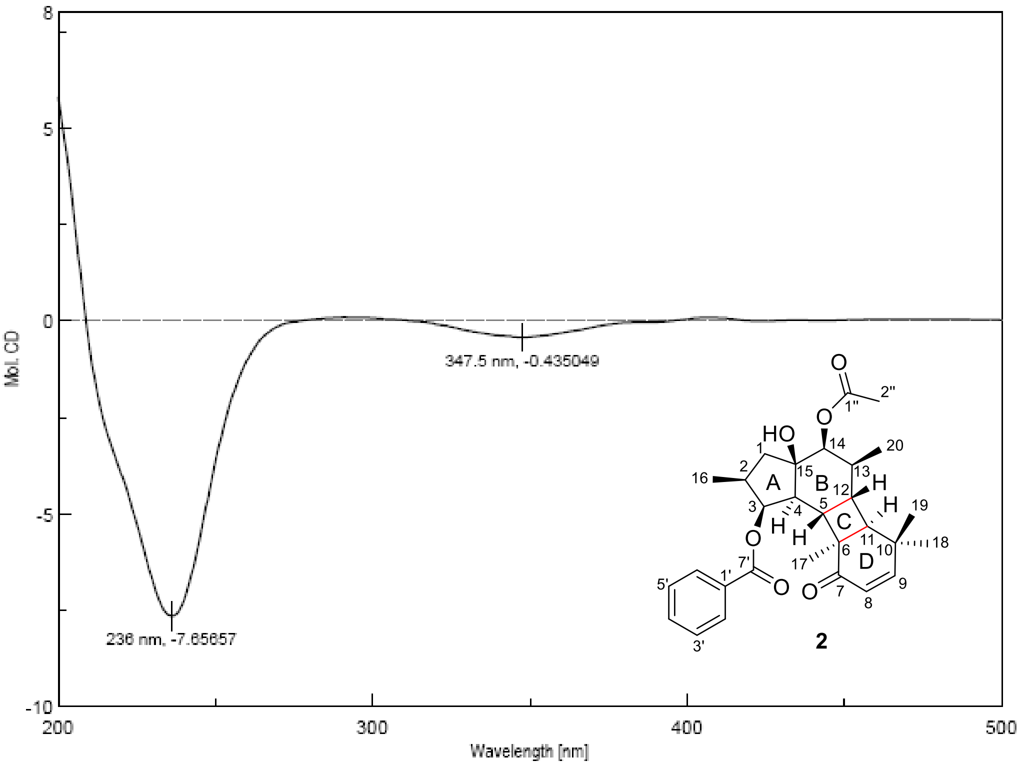

Figure S19. Experimental ECD spectrum of heliosterpenoid B (**2**)

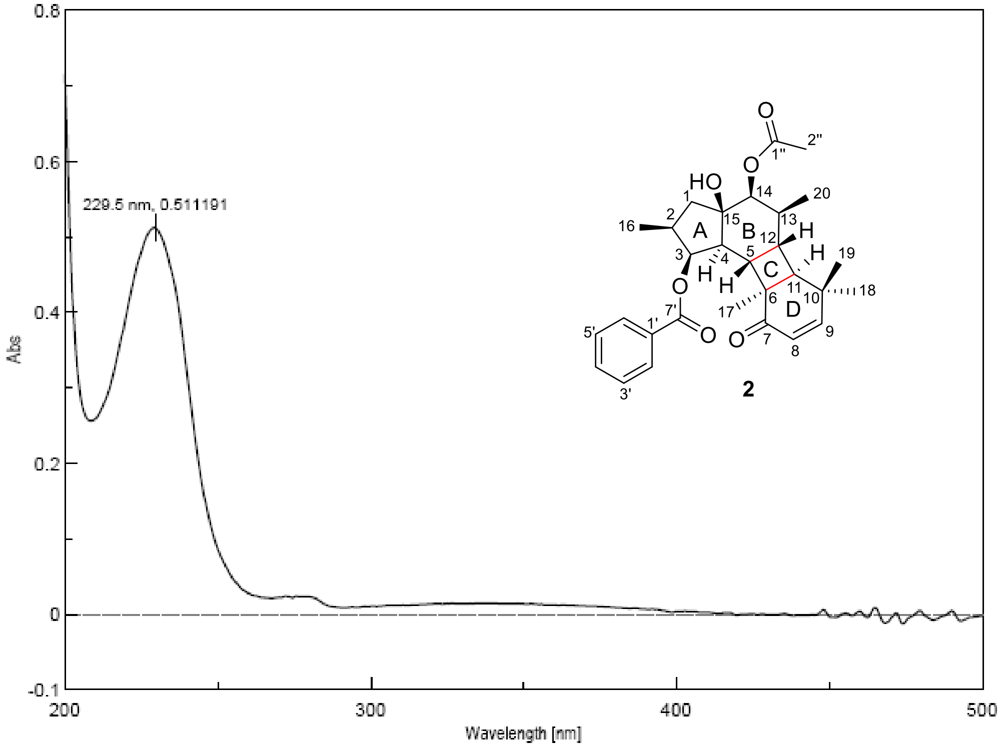

Figure S20. UV spectrum of heliosterpenoid B (**2**)

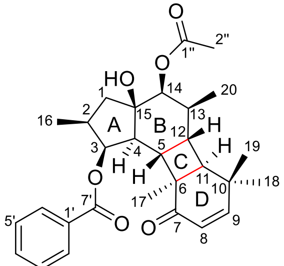

2

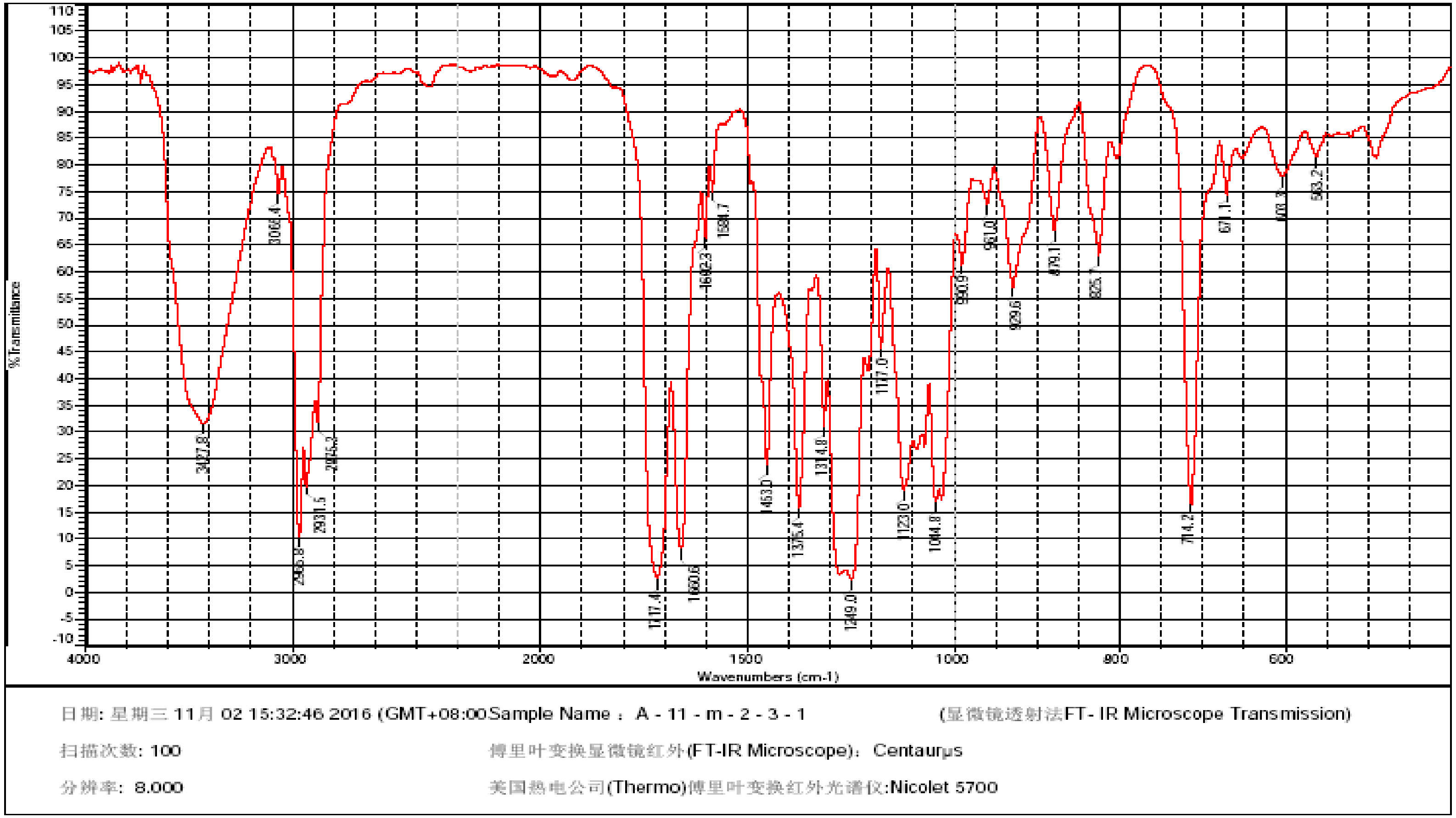

Figure S21. IR spectrum of heliosterpenoid B (**2**)

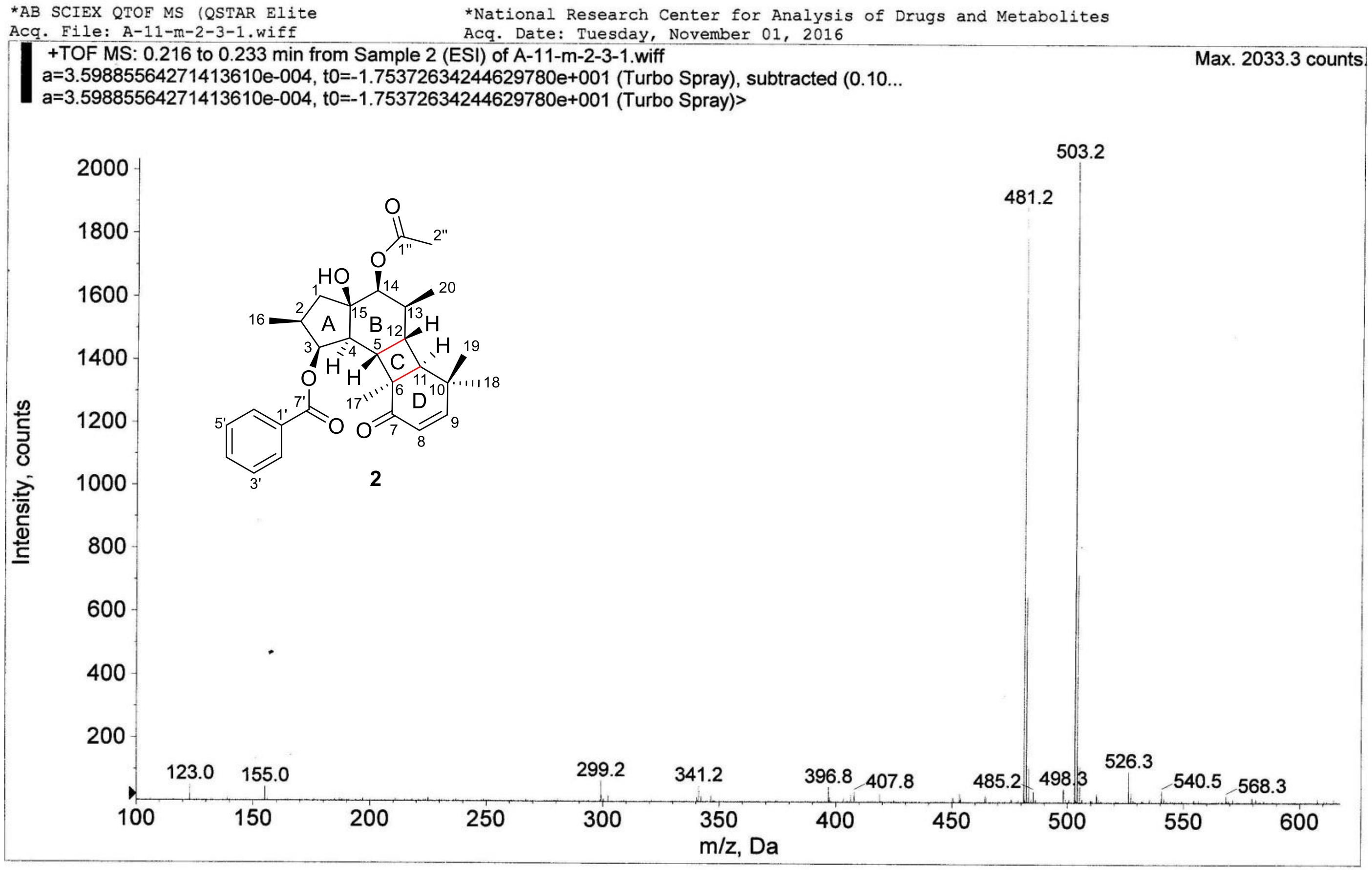

Figure S22. (+)-ESIMS data of heliosterpenoid B (**2**)

MS Formula Results: + Scan (7.830 min) Sub (2016092206.d)

m/z	Ion	Formula	Abundance
503.2423	(M+Na)+	C29 H36 Na O6	396529.2

	Best	Formula (M)	Ion Formula	Score	Cross Sco	Mass	Calc Mass	Calc m/z	Diff (ppm)	Abs Diff (ppm)	Mass Match	Abund Match	Spacing Match
+	✔	C29 H36 O6	C29 H36 Na O6	99.7		480.2531	480.2512	503.2404	-3.91	3.91	99.52	99.86	99.86
+		C26 H40 O6 S	C26 H40 Na O6 S	99.06		480.2531	480.2546	503.2438	3.1	3.1	99.7	97.56	99.57
+		C21 H40 N2 O8 S	C21 H40 N2 Na O8 S	97.98		480.2531	480.2505	503.2398	-5.29	5.29	99.13	94.79	99.5
+		C30 H40 O S2	C30 H40 Na O S2	97.56		480.2531	480.2521	503.2413	-2.11	2.11	99.86	92.24	99.34
+		C27 H44 O S3	C27 H44 Na O S3	95.04		480.2531	480.2554	503.2446	4.9	4.9	99.25	84.64	99.07
+		C22 H44 N2 O3 S3	C22 H44 N2 Na O3 S3	94.62		480.2531	480.2514	503.2406	-3.49	3.49	99.62	82.68	98.97

Figure S23. (+)-HRESIMS data of heliosterpenoid B (**2**)

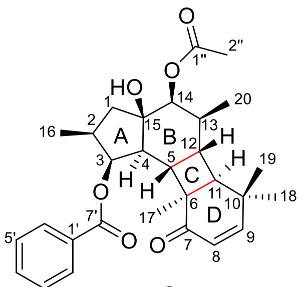

2

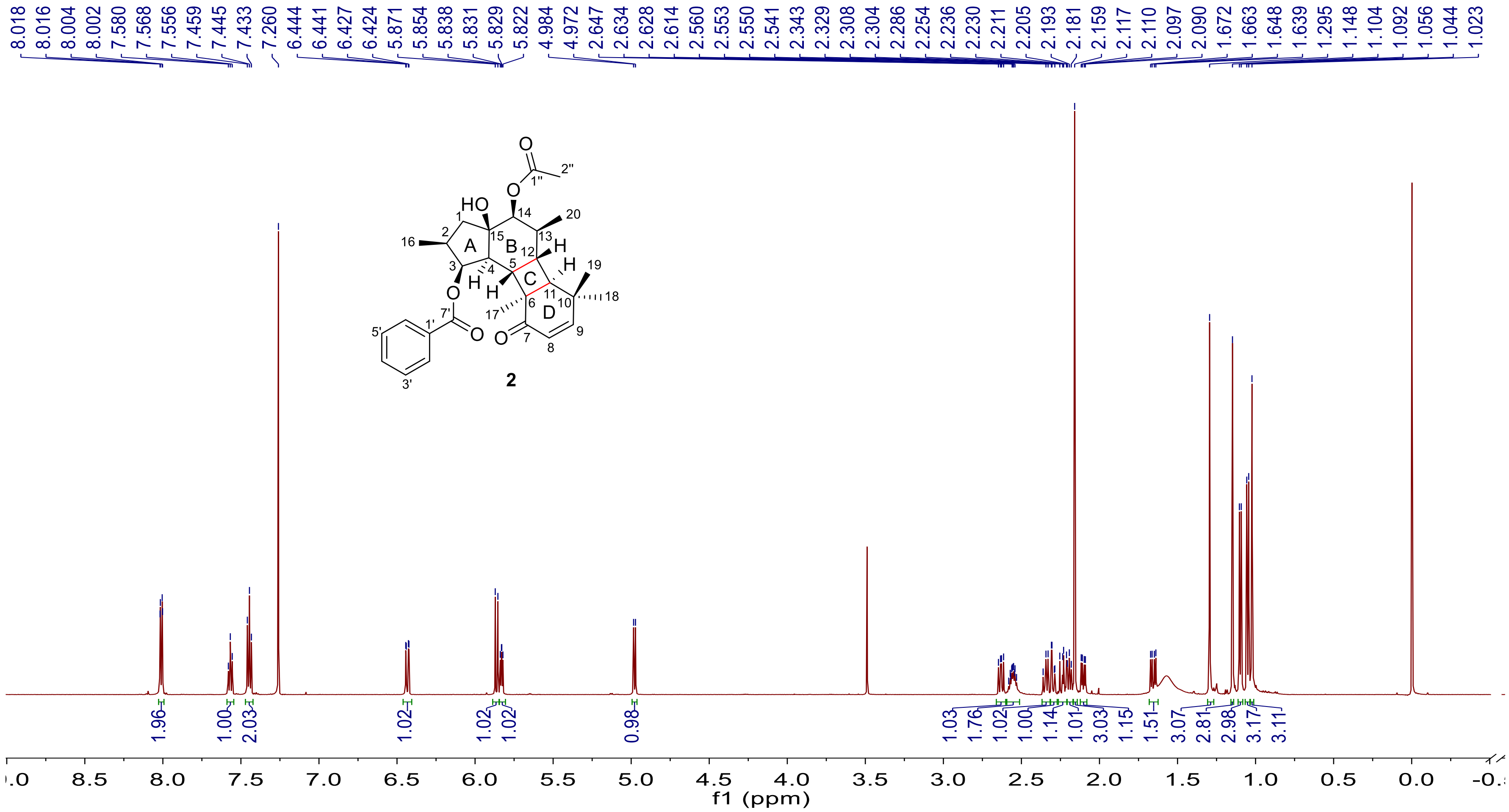

Figure S24. ^{1}H NMR spectrum of heliosterpenoid B (**2**) in $CDCl_3$ (600 MHz)

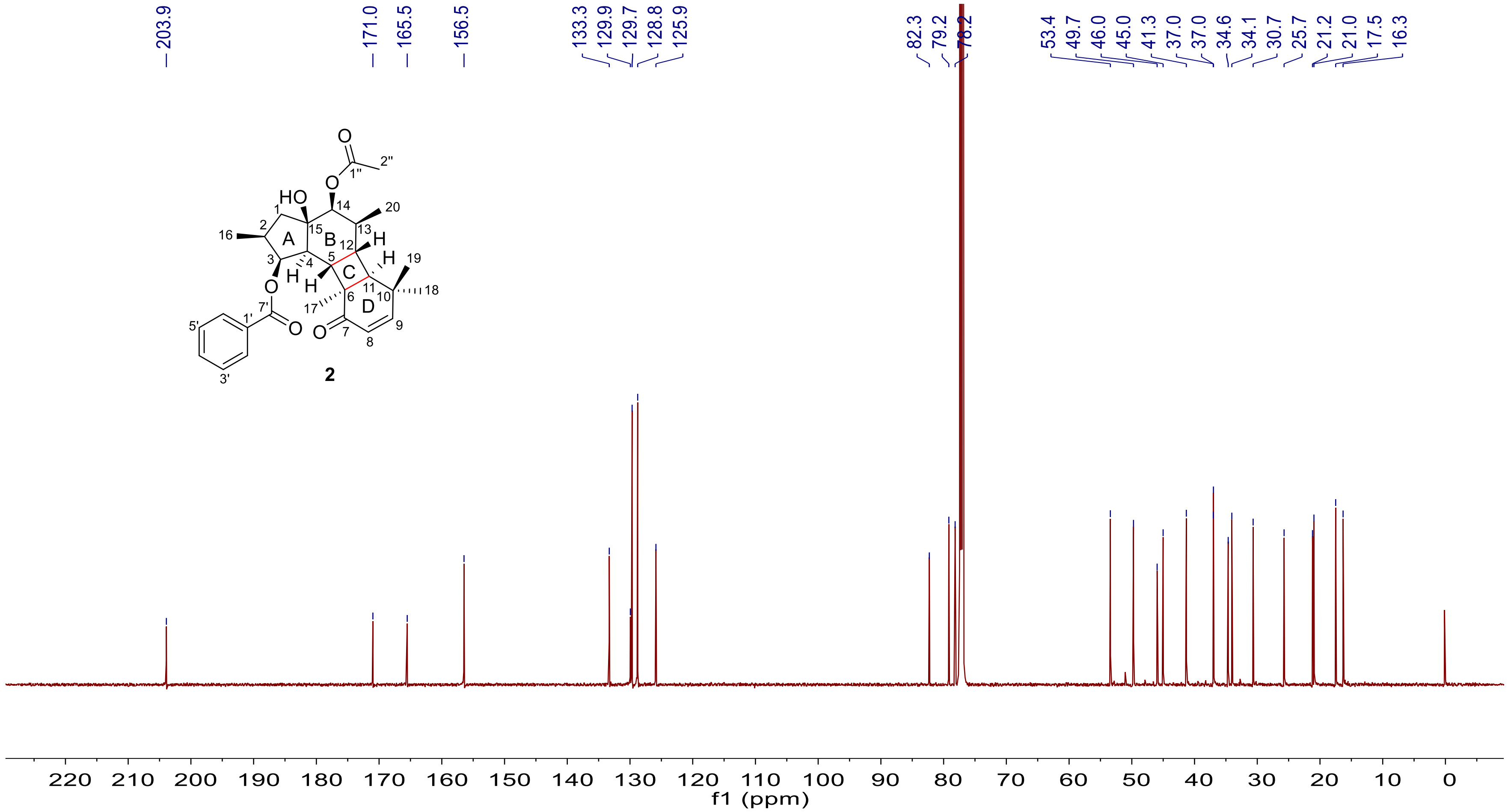

Figure S25. ^{13}C NMR spectrum of heliosterpenoid B (**2**) in $CDCl_3$ (150 MHz)

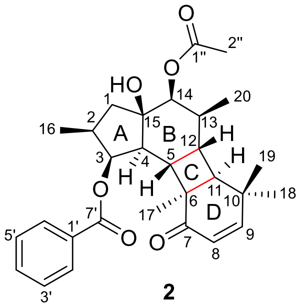

2

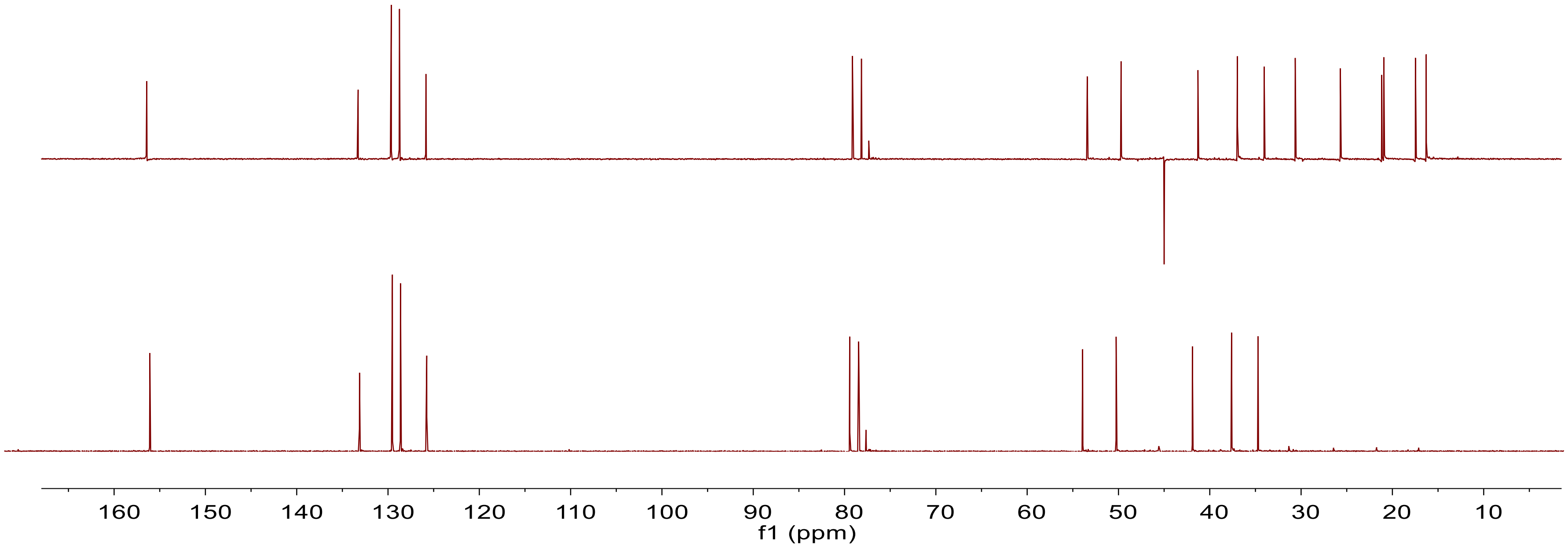

Figure S26. DEPT spectrum of heliosterpenoid B (**2**) in $CDCl_3$ (150 MHz)

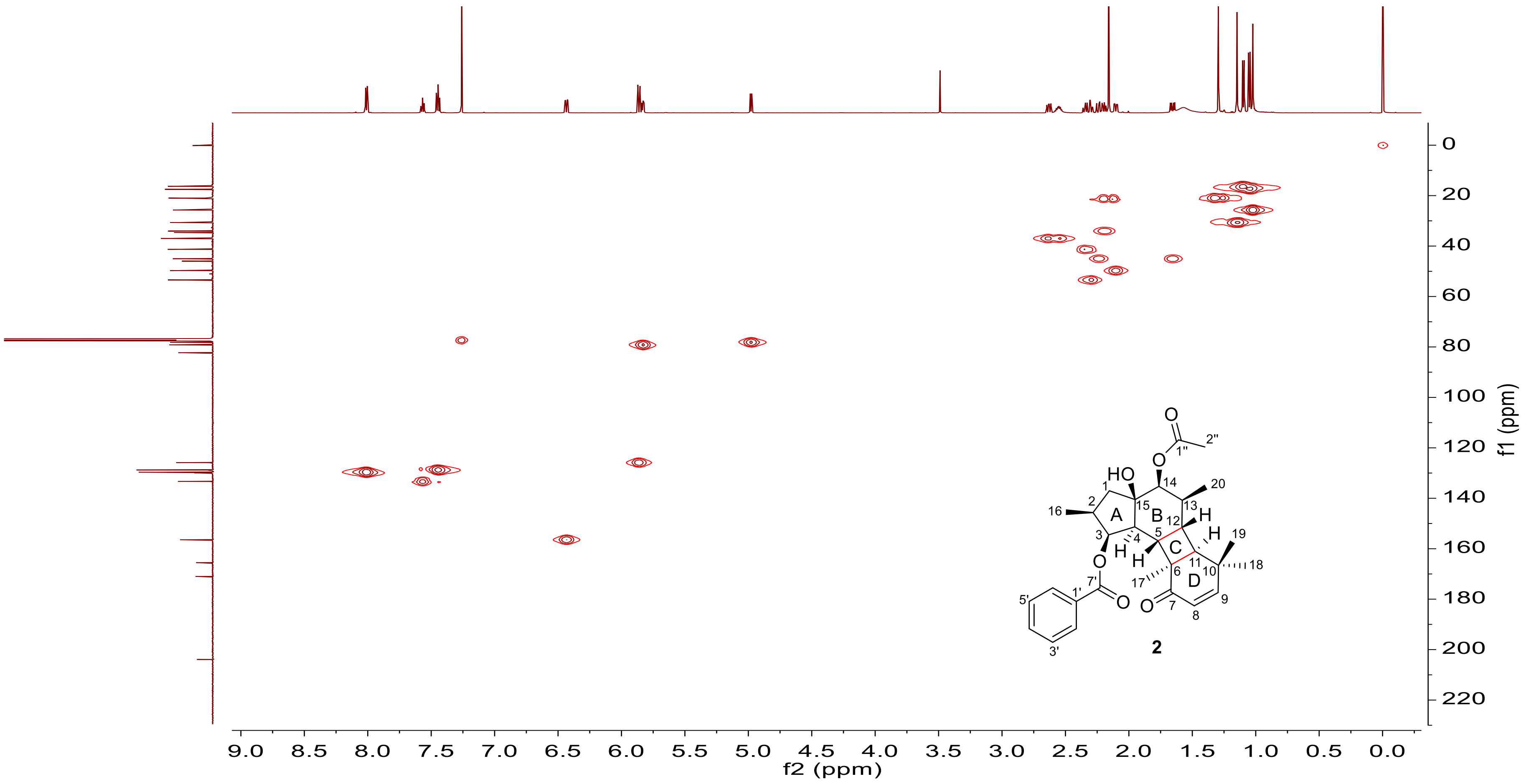

Figure S27. HSQC spectrum of heliosterpenoid B (**2**) in $CDCl_3$ (^{1}H: 600 MHz, ^{13}C: 150 MHz)

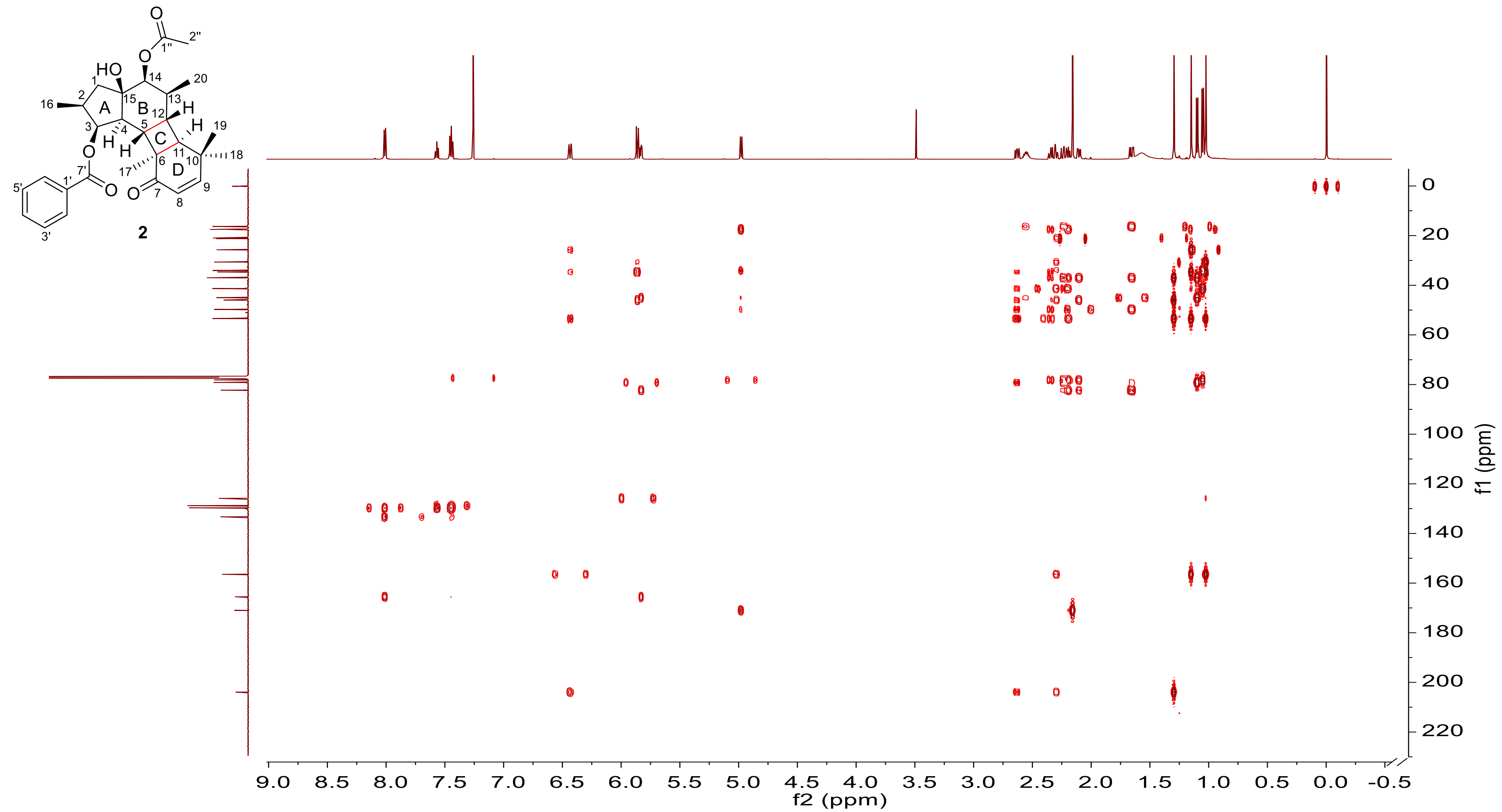

Figure S28. HMBC spectrum of heliosterpenoid B (**2**) in $CDCl_3$ (^1H: 600 MHz, ^{13}C: 150 MHz)

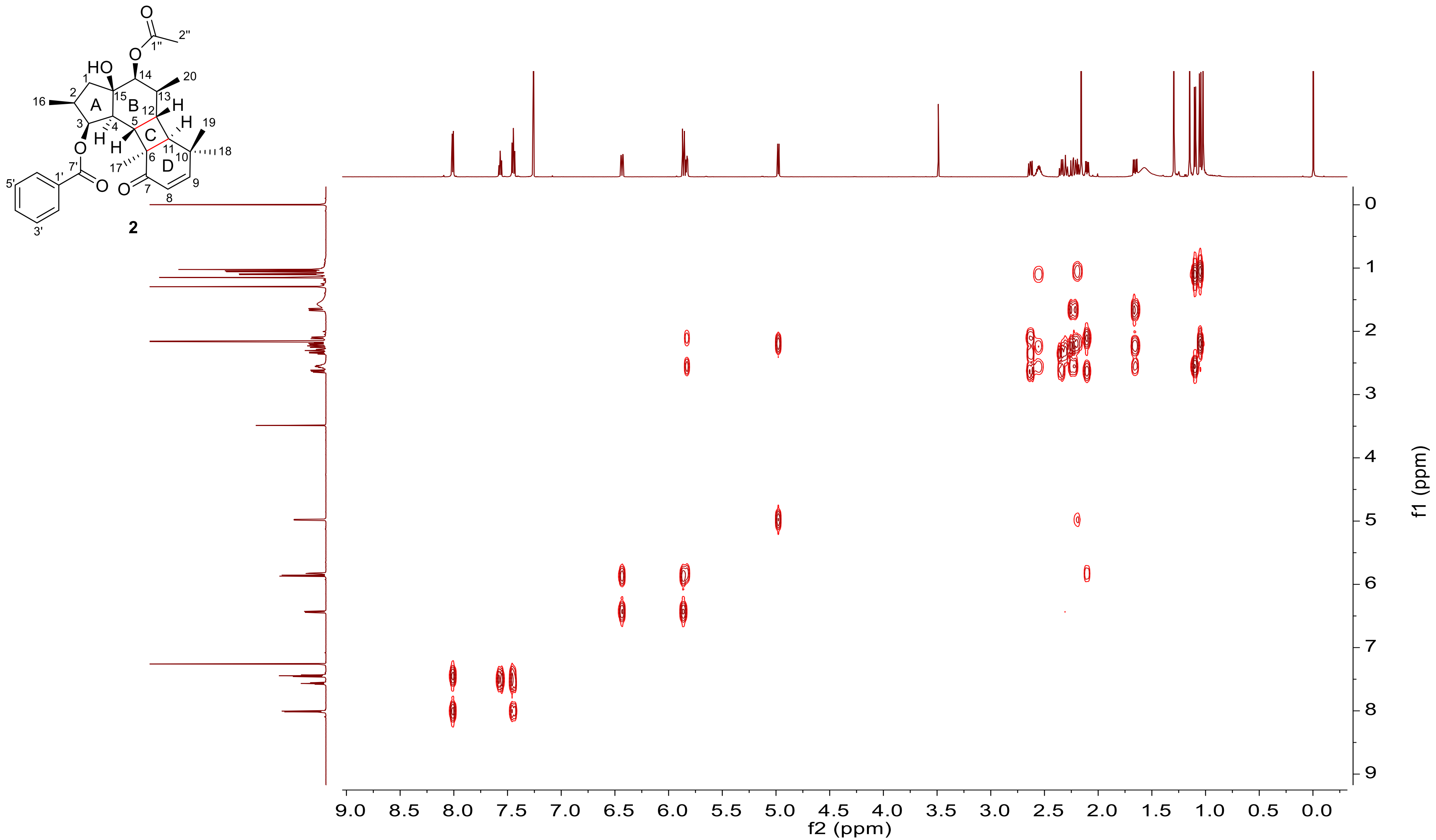

Figure S29. ^1H-^1H COSY spectrum of heliosterpenoid B (**2**) in $CDCl_3$ (600 MHz)

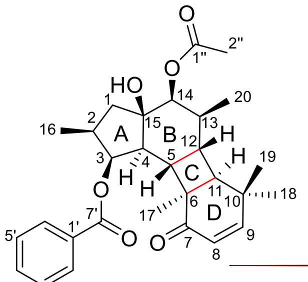

2

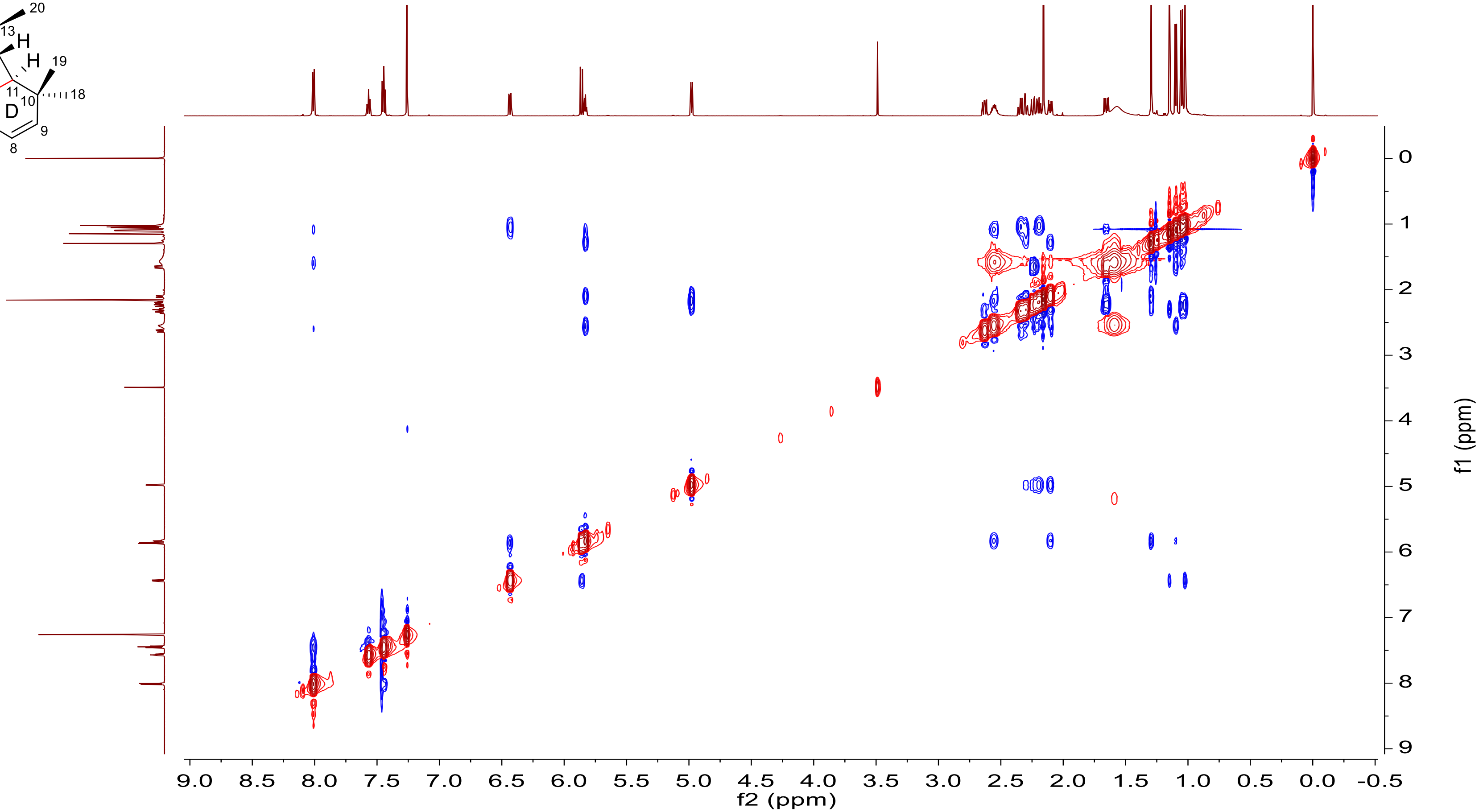

Figure S30. NOESY spectrum of heliosterpenoid B (**2**) in $CDCl_3$ (600 MHz)

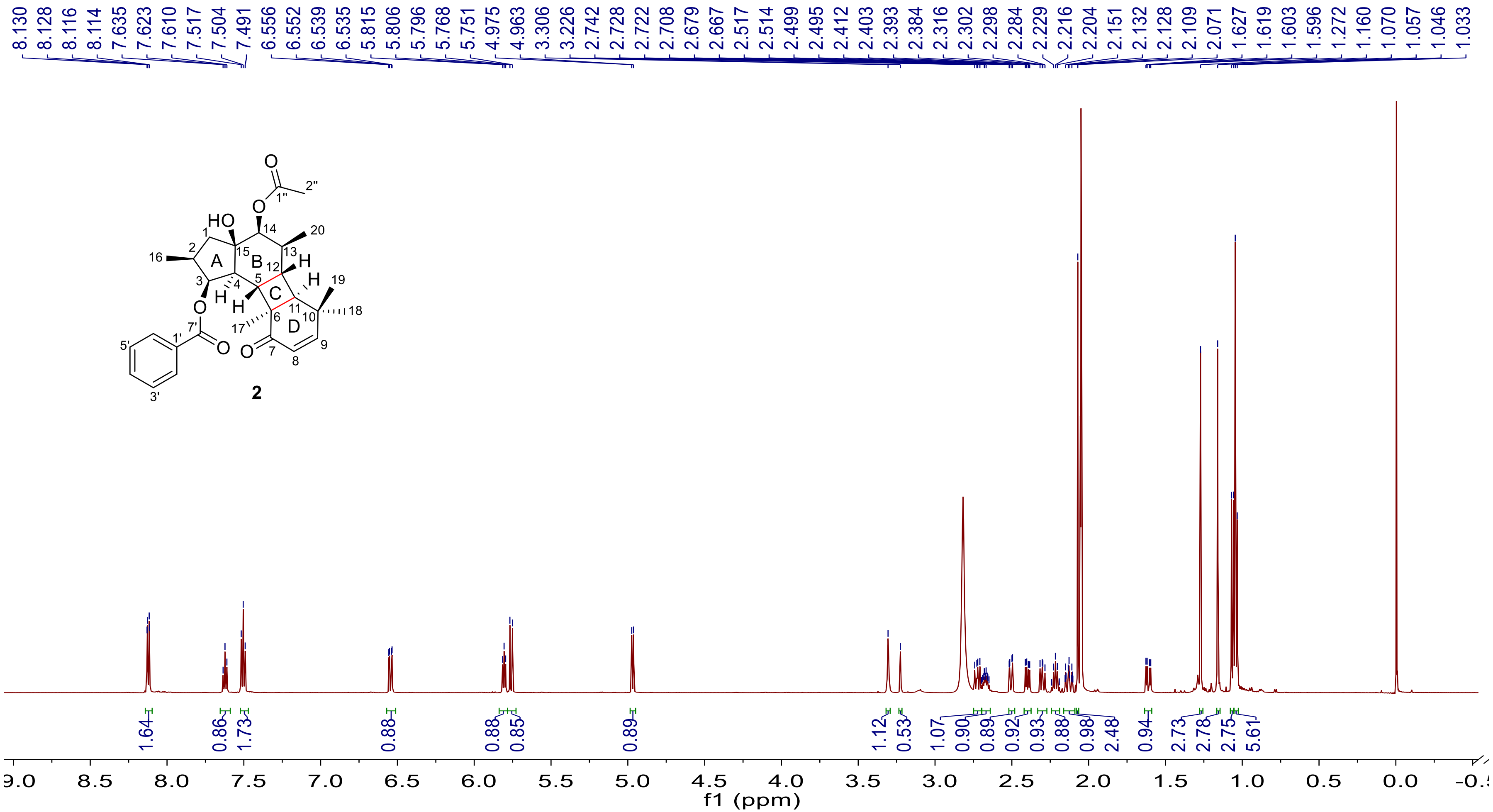

Figure S31. ^{1}H NMR spectrum of heliosterpenoid B (**2**) in acetone-d_6 (600 MHz)

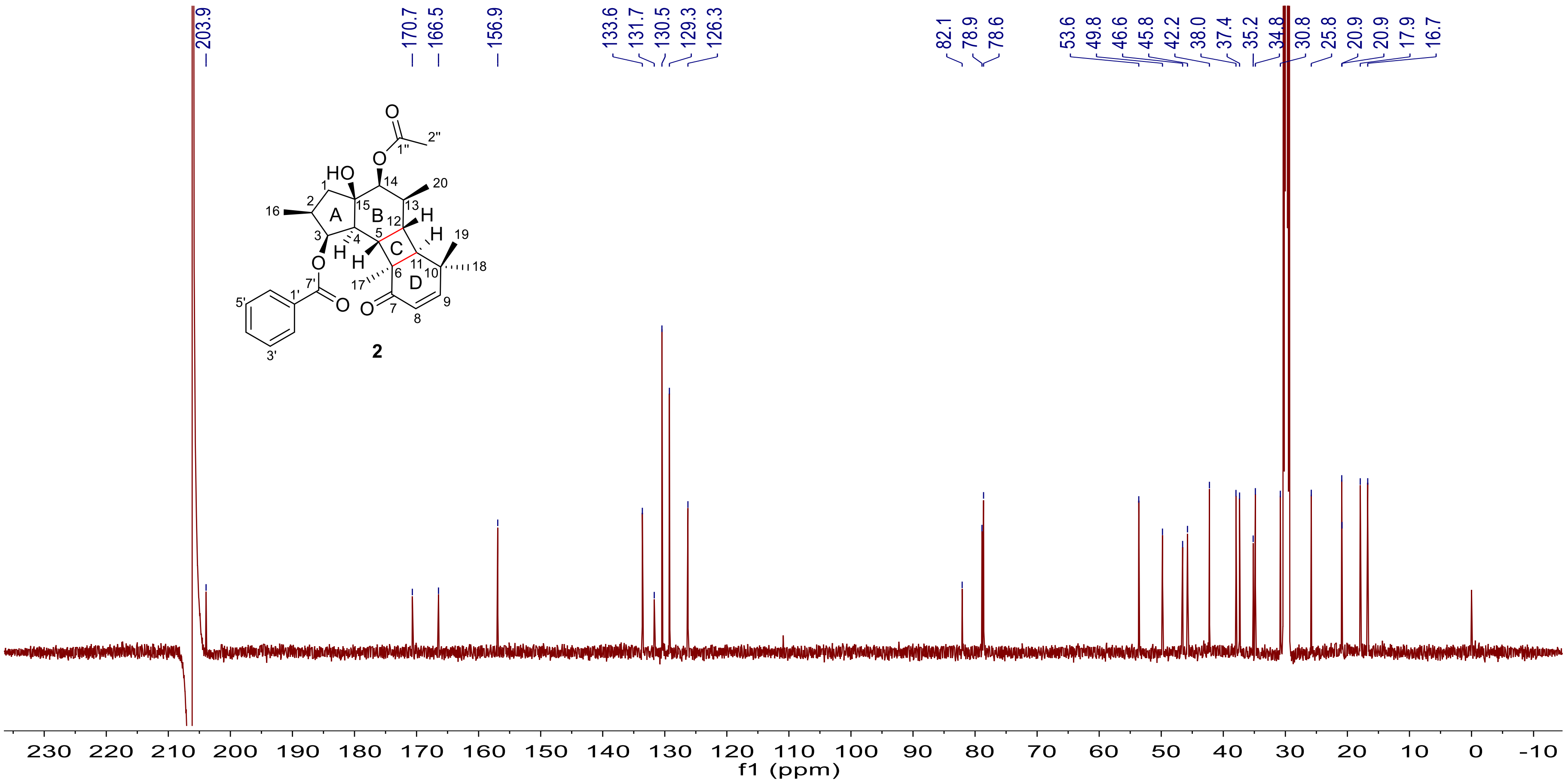

Figure S32. ^{13}C NMR spectrum of heliosterpenoid B (**2**) in acetone-d_6 (150 MHz)

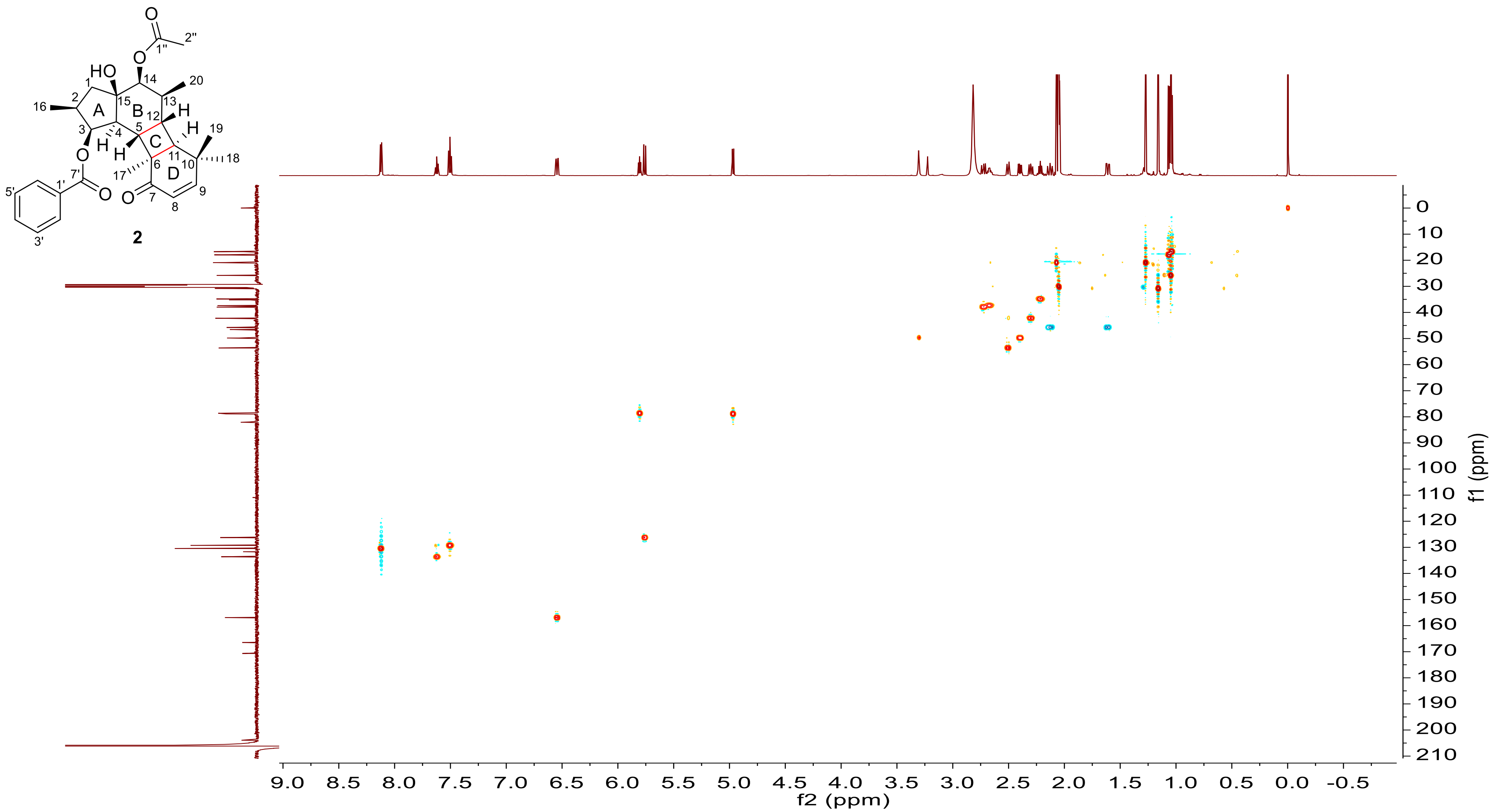

Figure S33. HSQC spectrum of heliosterpenoid B (**2**) in acetone-d_6 (^{1}H: 600 MHz, ^{13}C: 150 MHz)

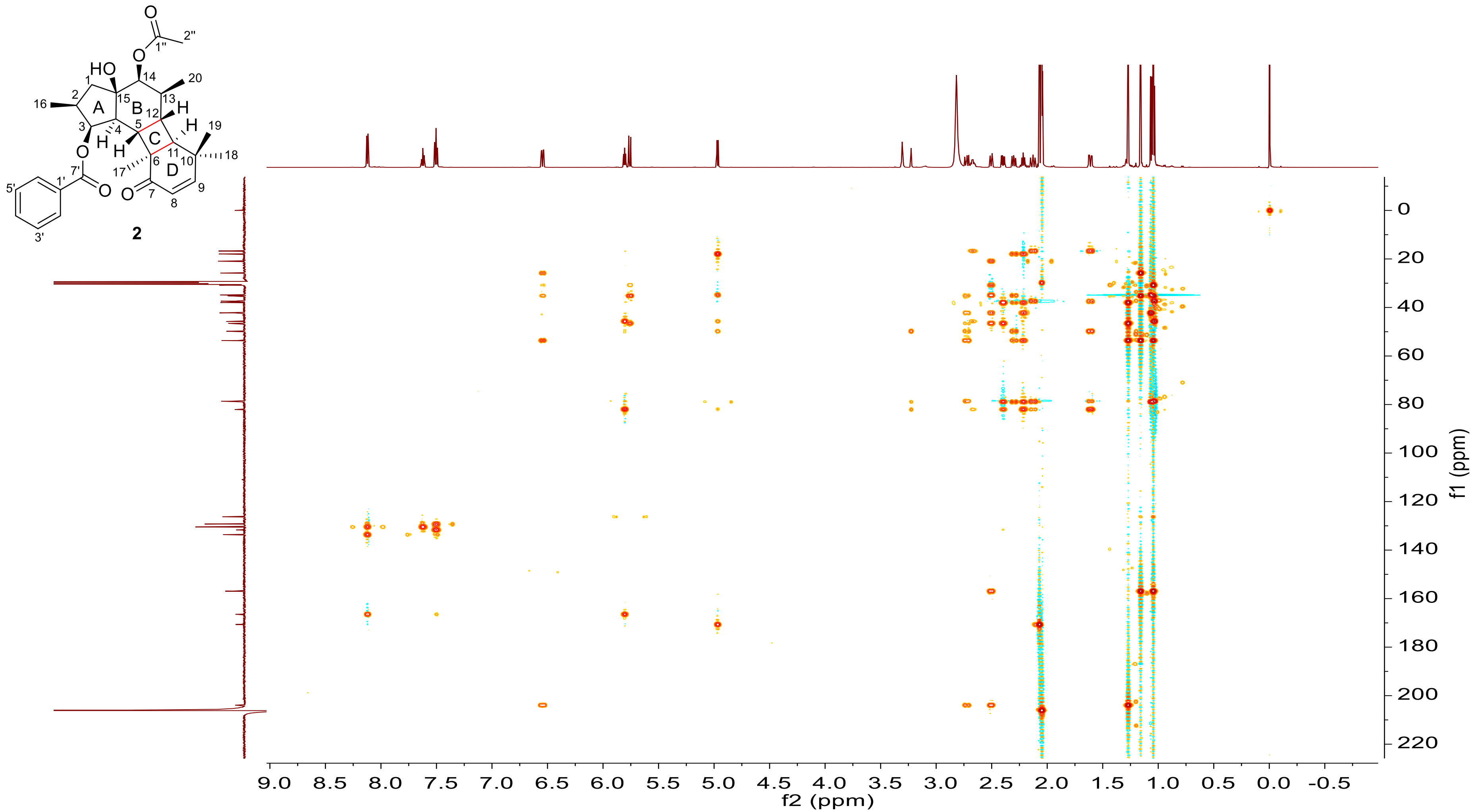

Figure S34. HMBC spectrum of heliosterpenoid B (**2**) in acetone-d_6 (^{1}H: 600 MHz, ^{13}C: 150 MHz)

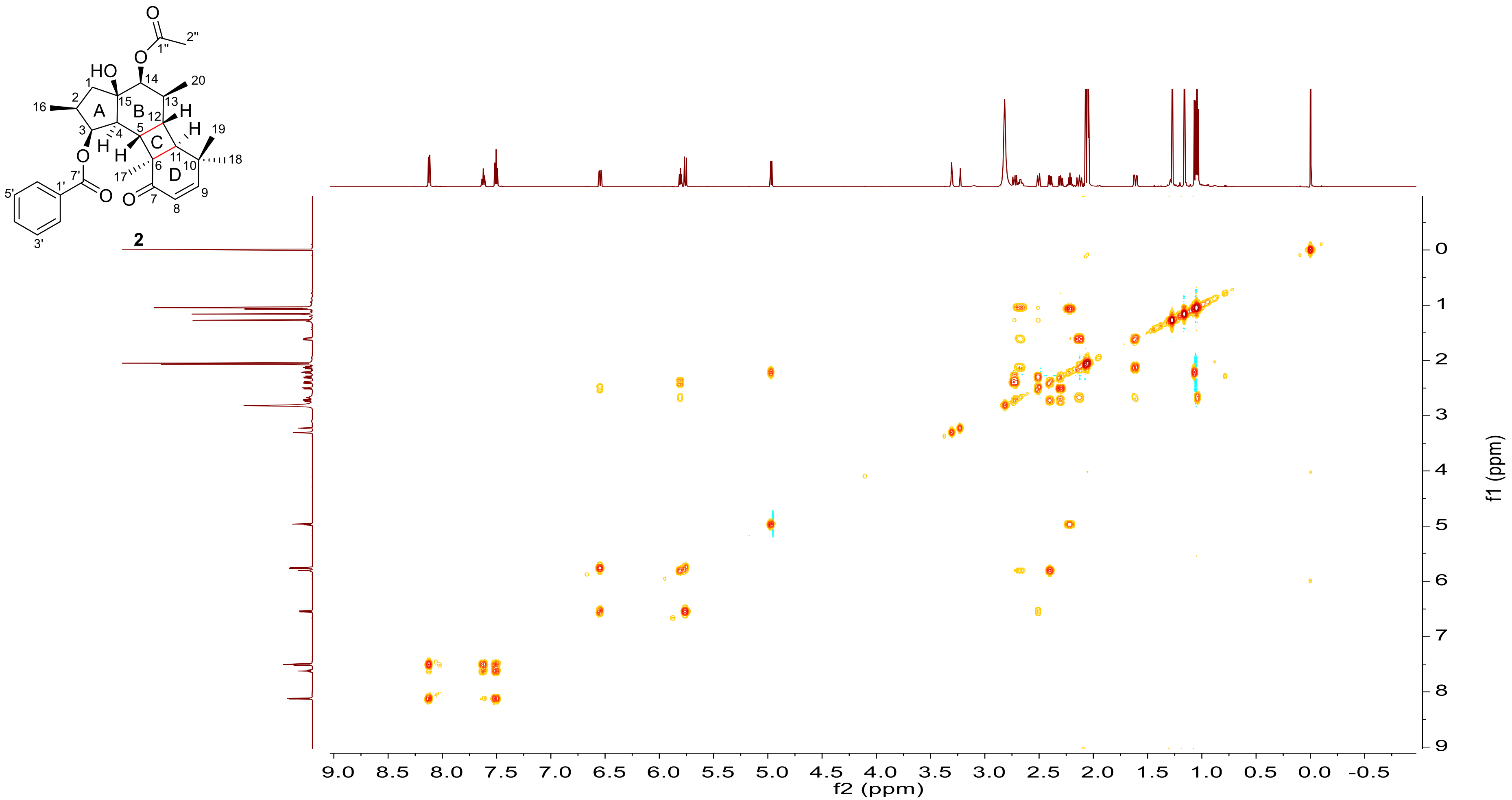

Figure S35. ^{1}H-^{1}H COSY spectrum of heliosterpenoid B (**2**) in acetone-d_6 (600 MHz)

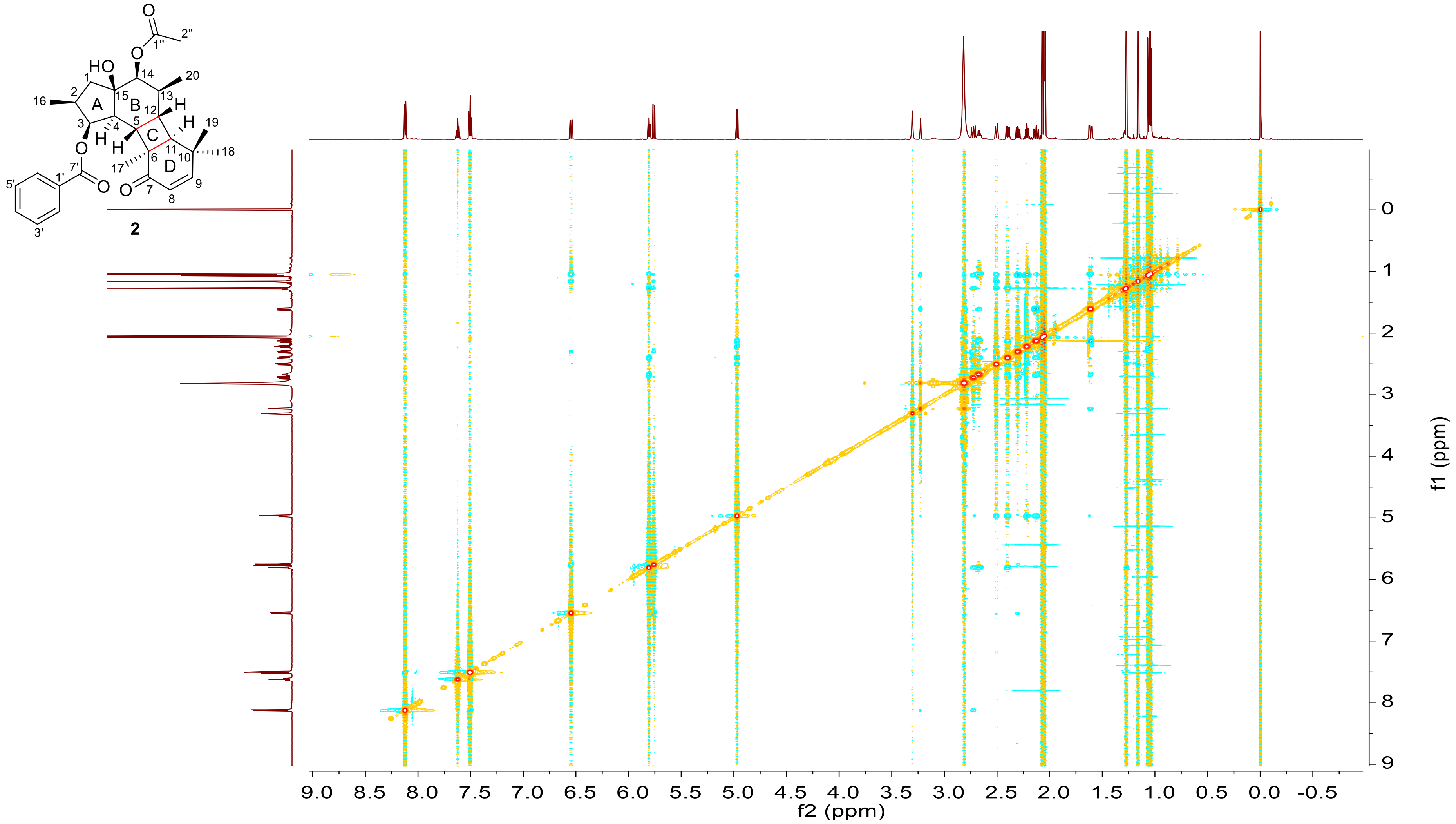

Figure S36. NOESY spectrum of heliosterpenoid B (**2**) in acetone-d_6 (600 MHz)

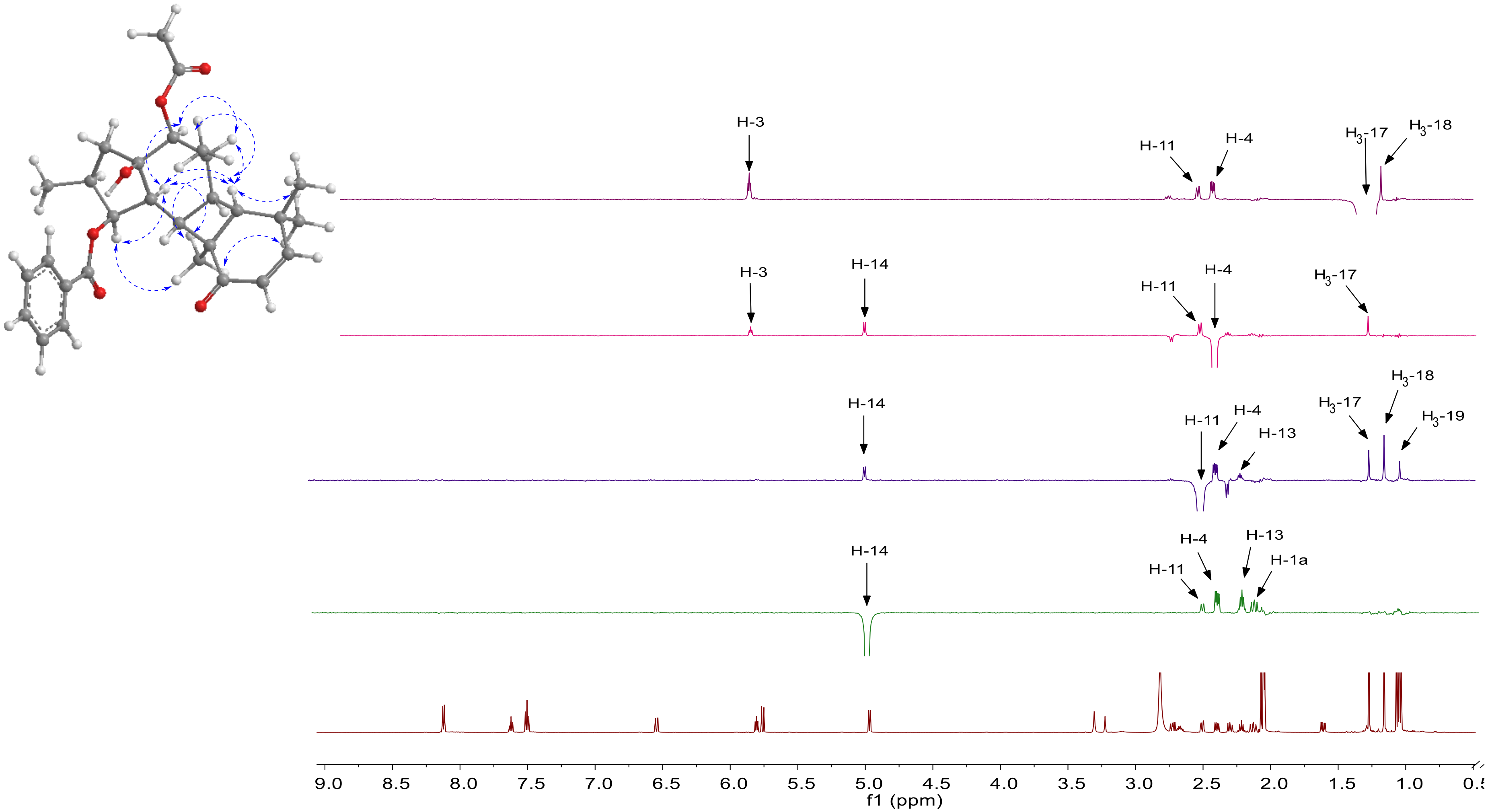

Figure S37. NOE difference spectrum 1 of heliosterpenoid B (**2**) in acetone-d_6 (600 MHz)

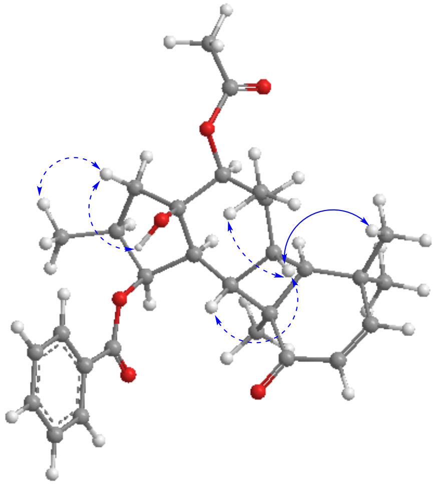

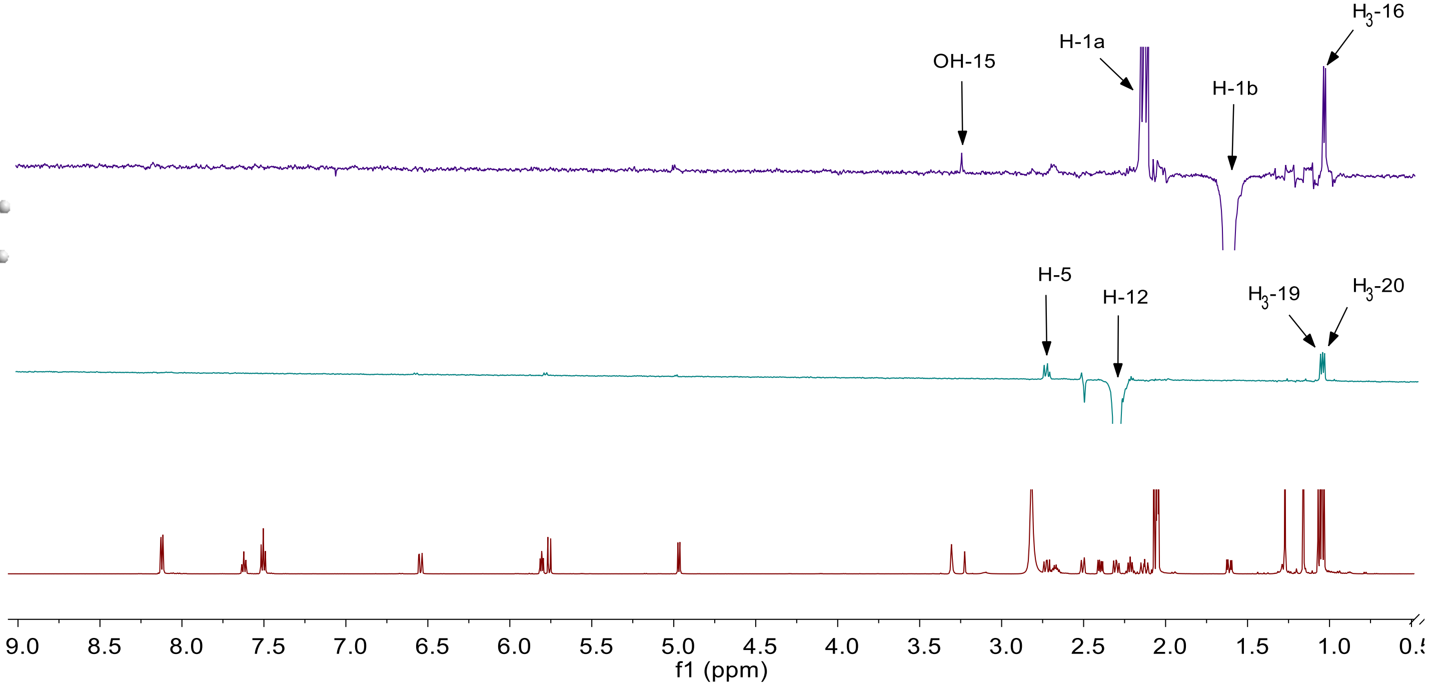

Figure S38. NOE difference spectrum 2 of heliosterpenoid B (**2**) in acetone-d_6 (600 MHz)

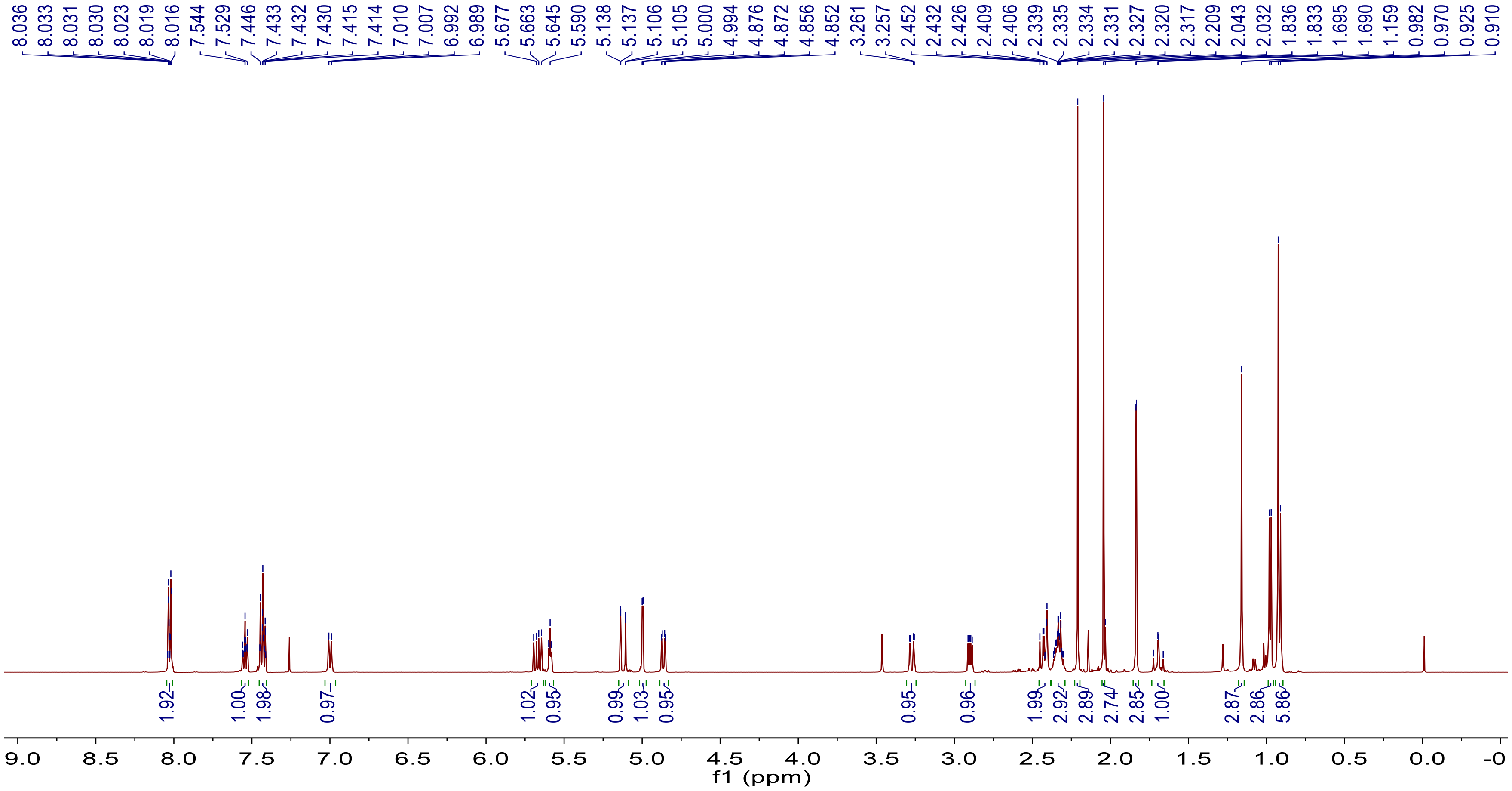

Figure S39. ^{1}H NMR spectrum of euphornin C in $CDCl_3$ (500 MHz)

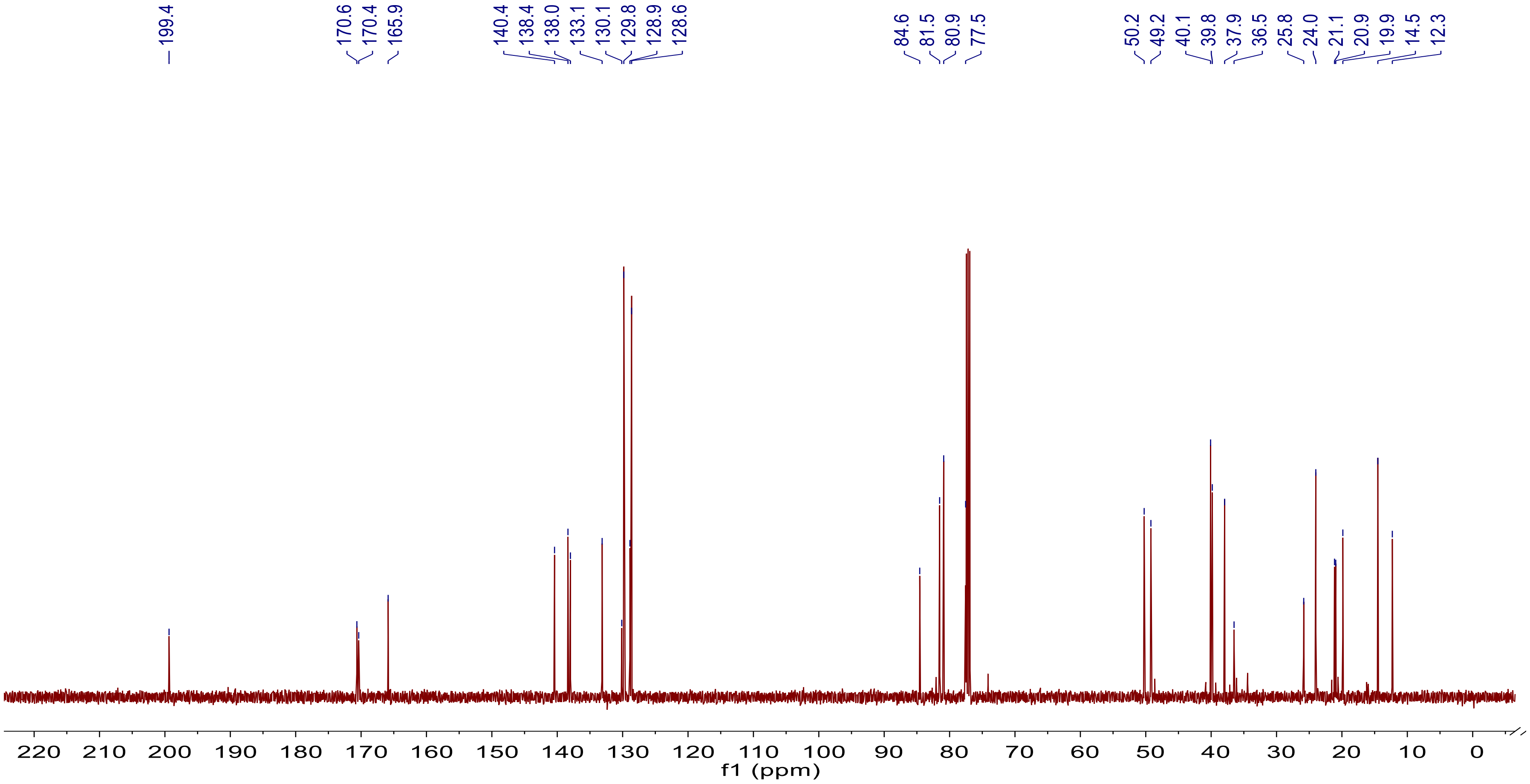

Figure S40. ^{13}C NMR spectrum of euphornin C in $CDCl_3$ (500 MHz)

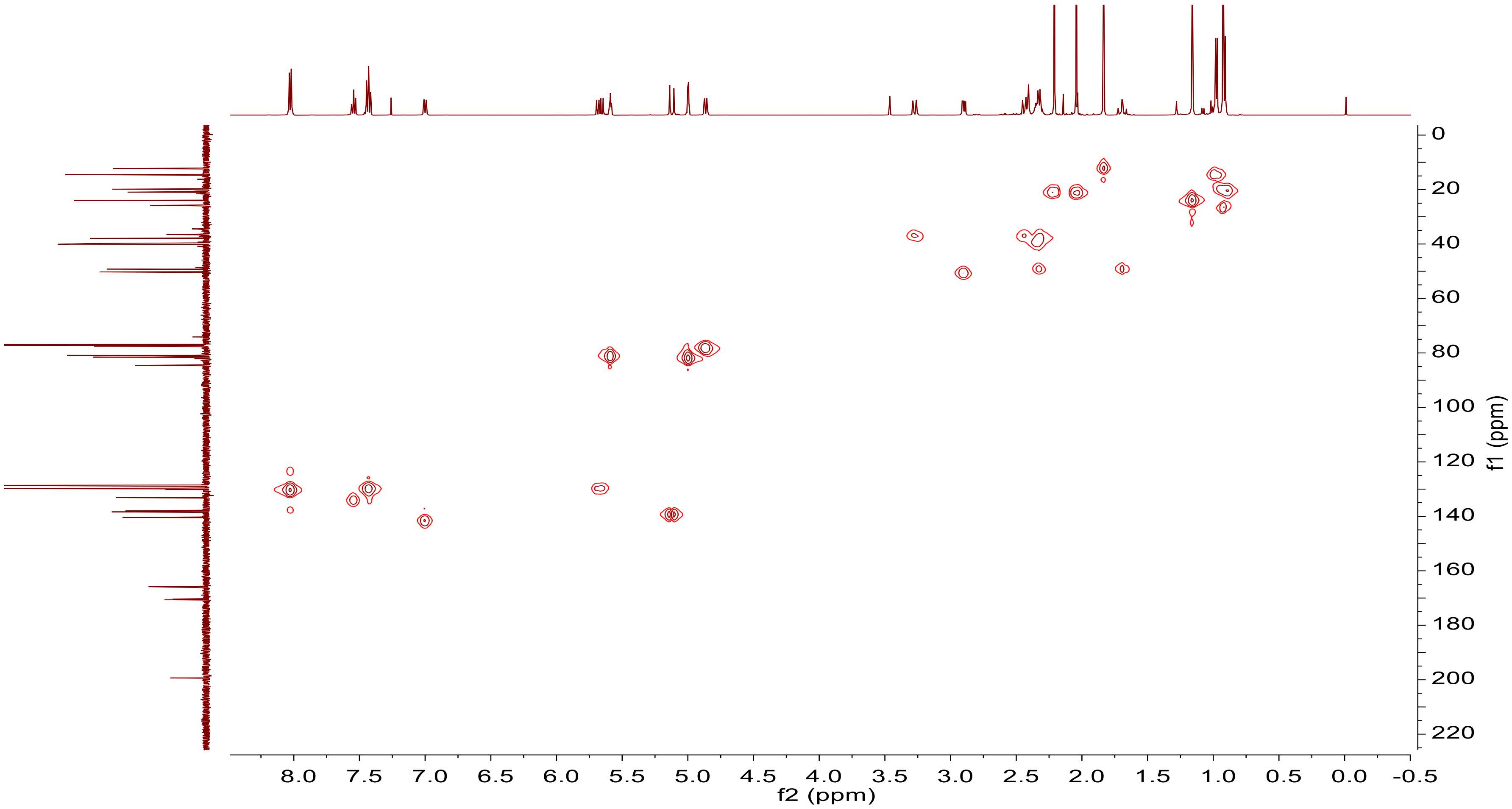

Figure S41. HSQC spectrum of euphornin C in $CDCl_3$ (500 MHz)

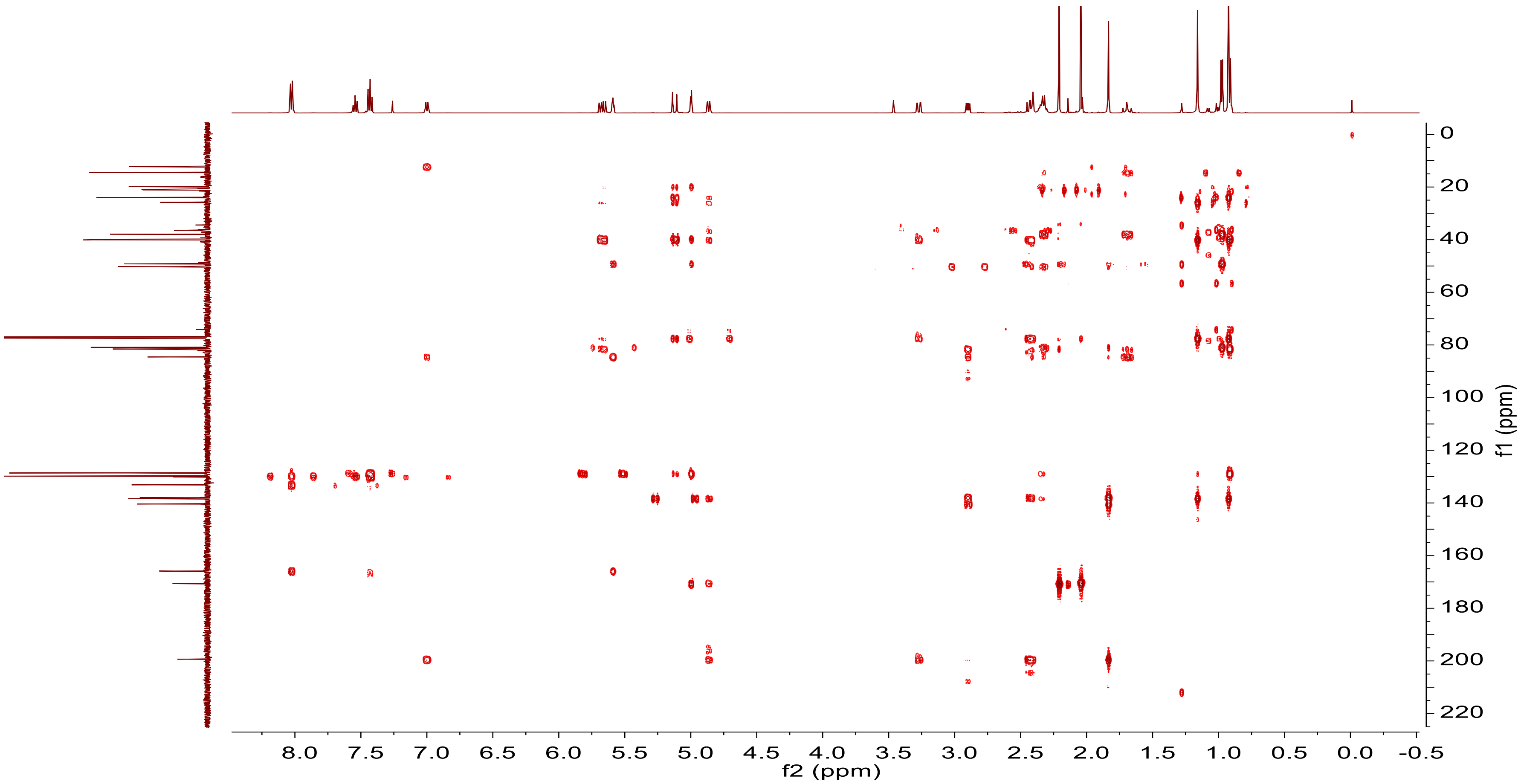

Figure S42. HMBC spectrum of euphornin C in $CDCl_3$ (500 MHz)

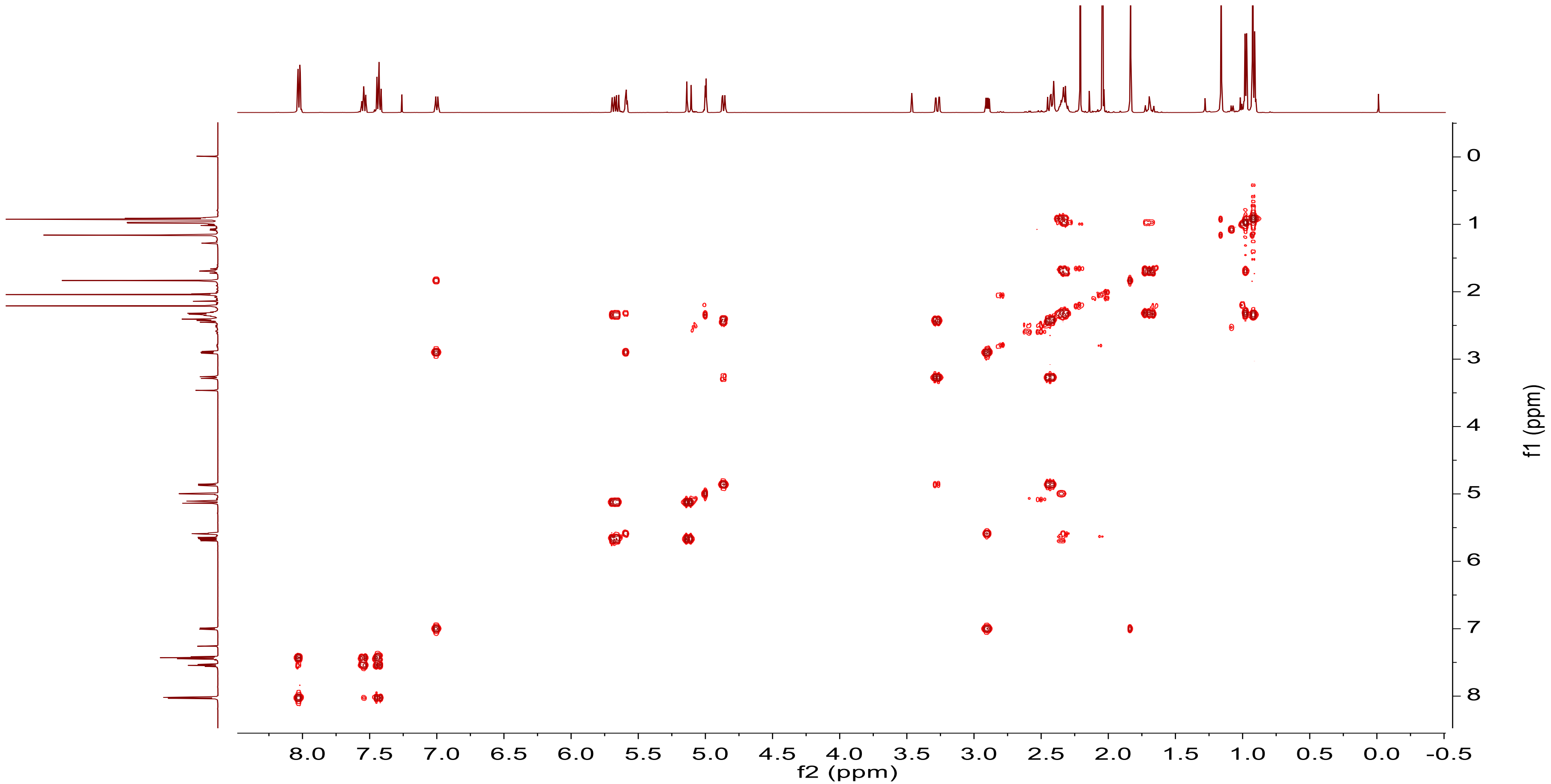

Figure S43. ^{1}H-^{1}H COSY spectrum of euphornin C in $CDCl_3$ (500 MHz)

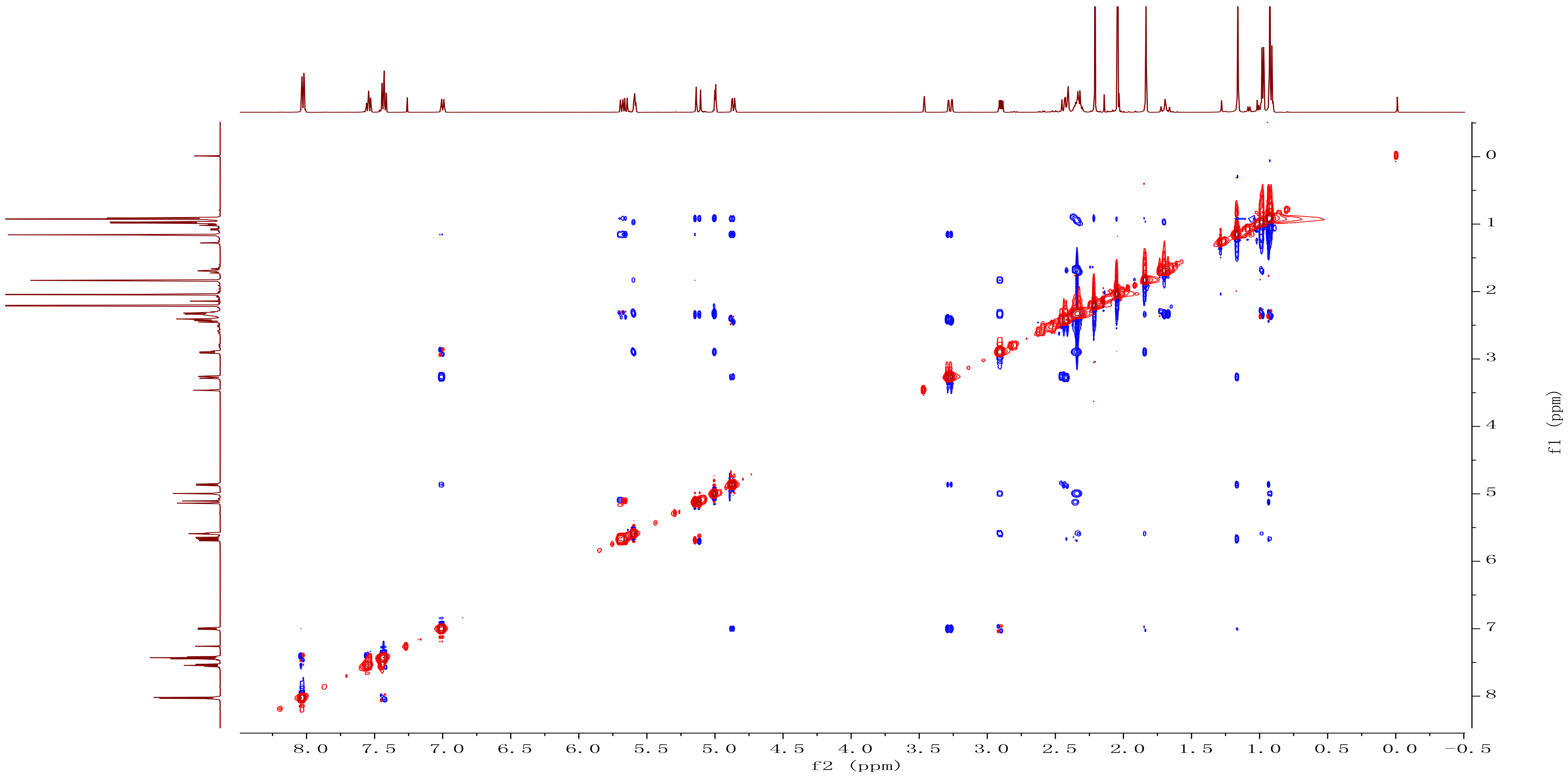

Figure S44. NOESY spectrum of euphornin C in $CDCl_3$ (500 MHz)

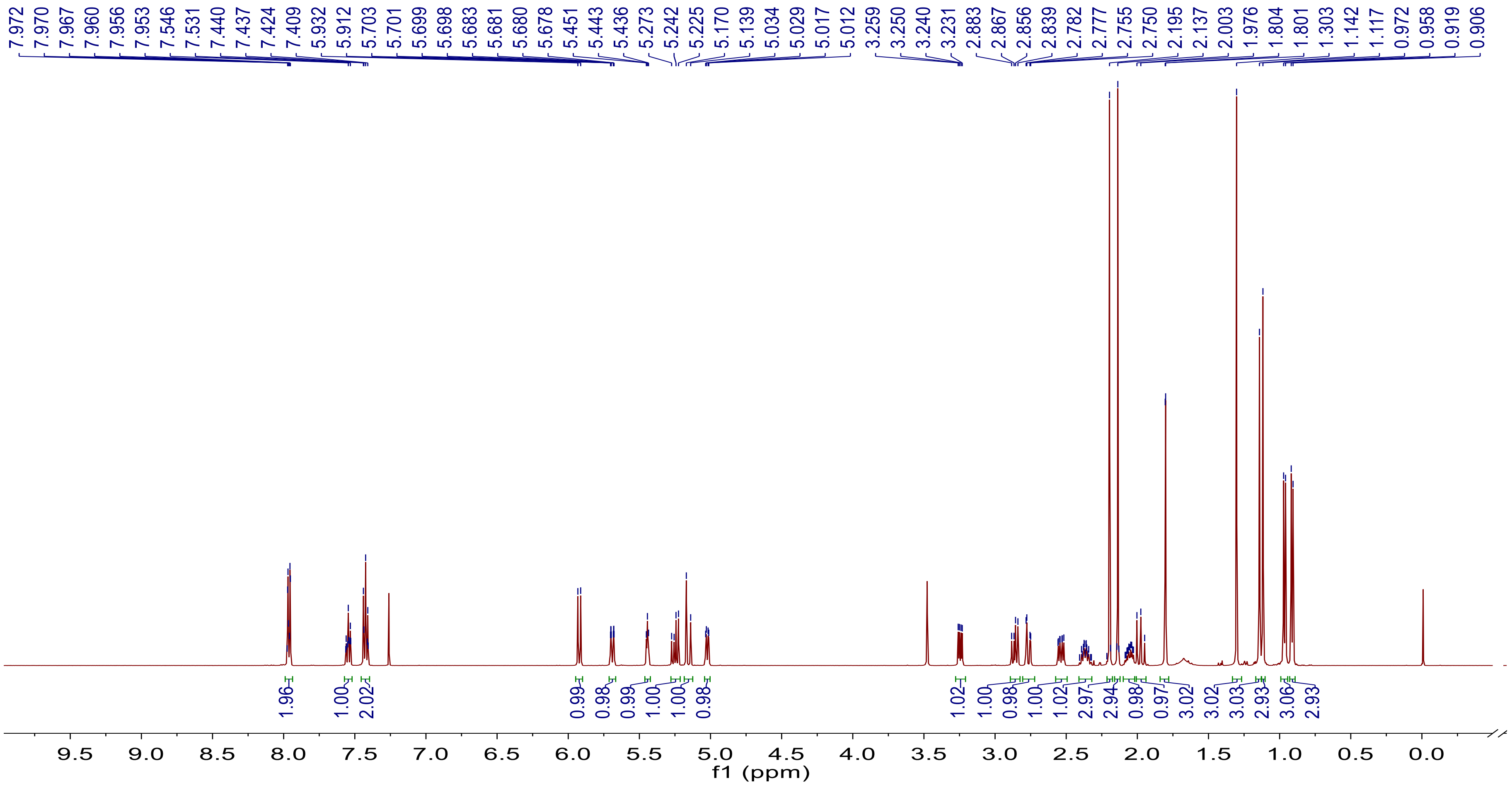

Figure S45. ^{1}H NMR spectrum of euphornin H in $CDCl_3$ (500 MHz)

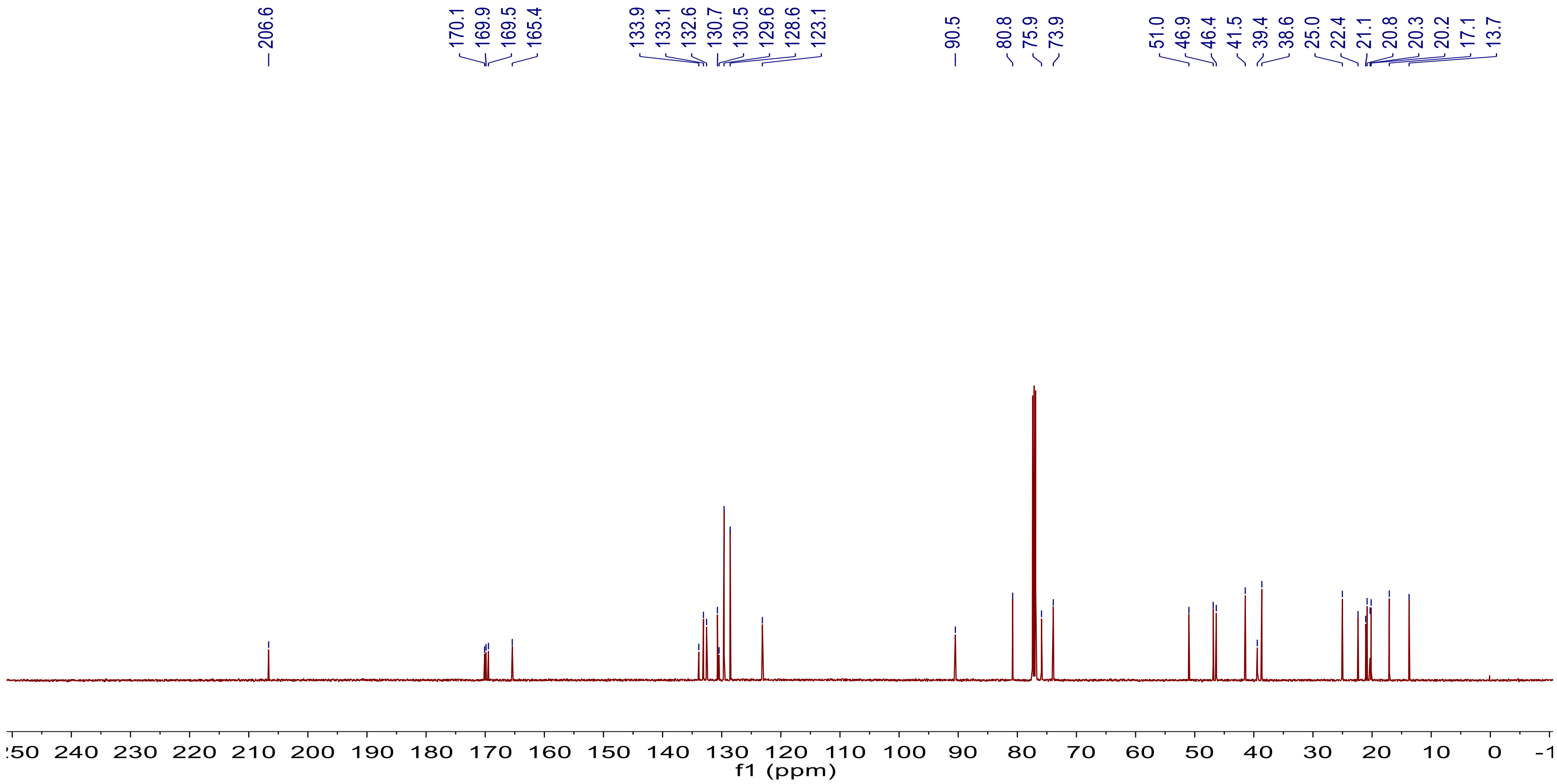

Figure S46. ^{13}C NMR spectrum of euphornin H in $CDCl_3$ (500 MHz)

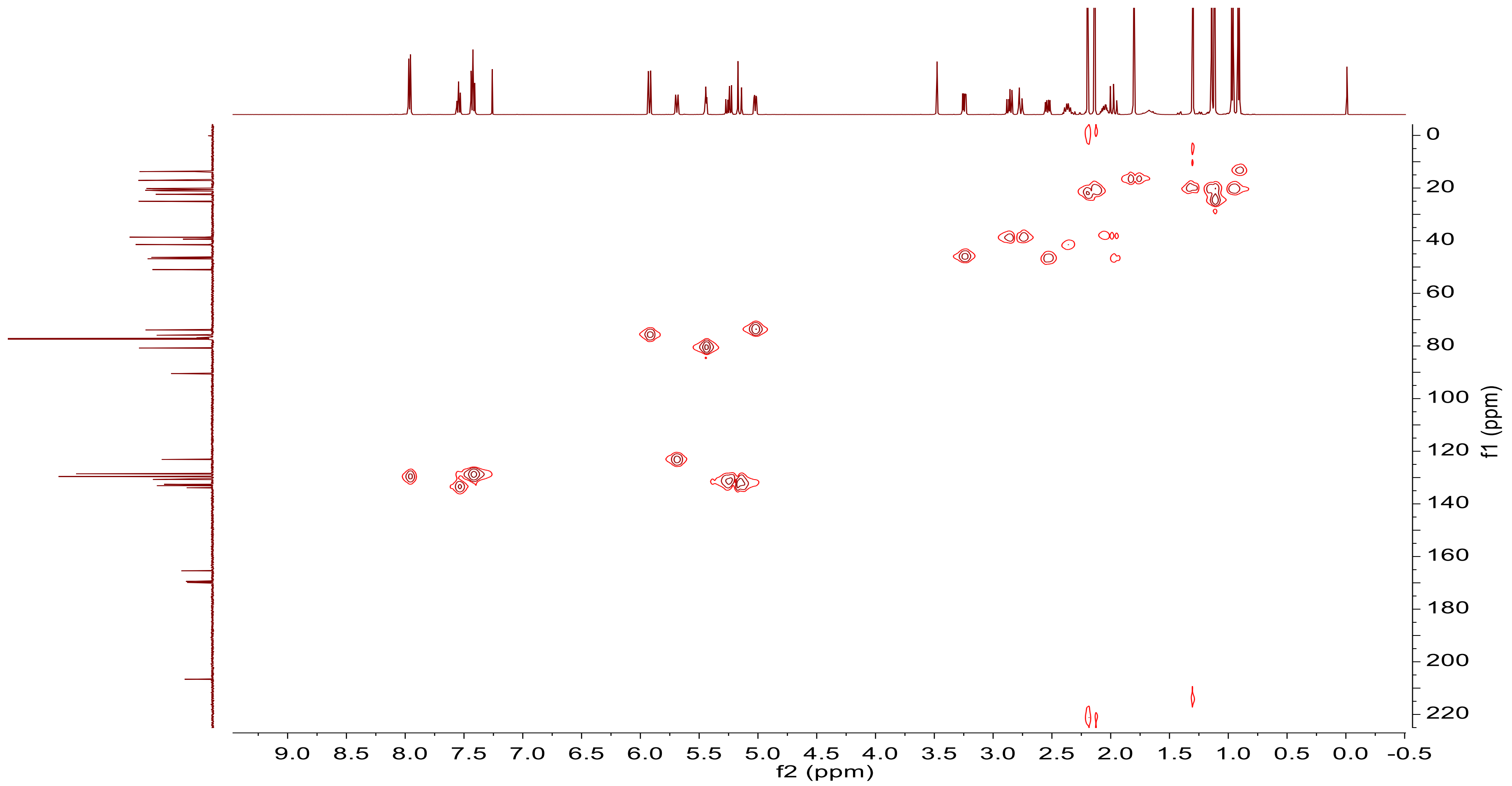

Figure S47. HSQC spectrum of euphornin H in $CDCl_3$ (500 MHz)

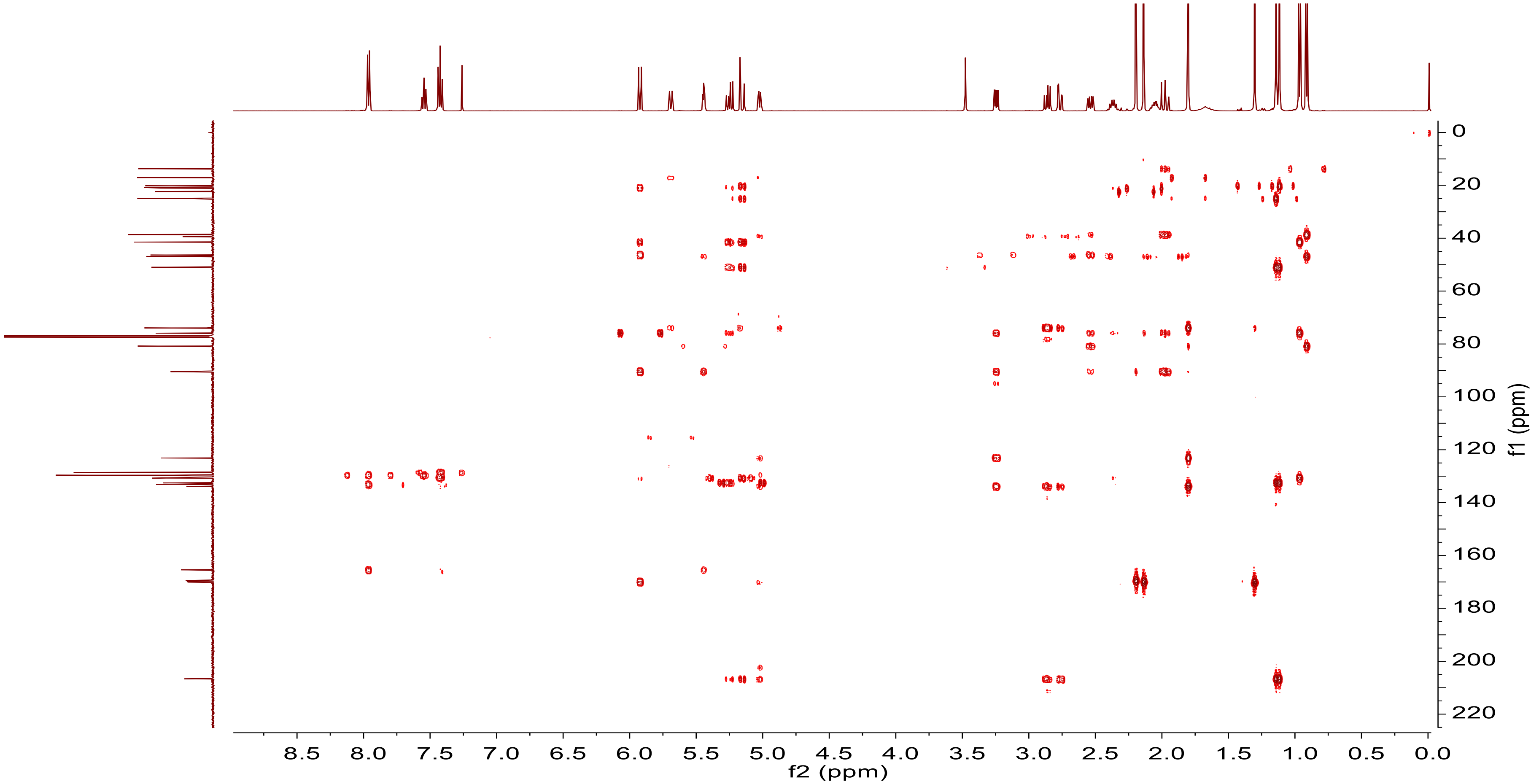

Figure S48. HMBC spectrum of euphornin H in $CDCl_3$ (500 MHz)

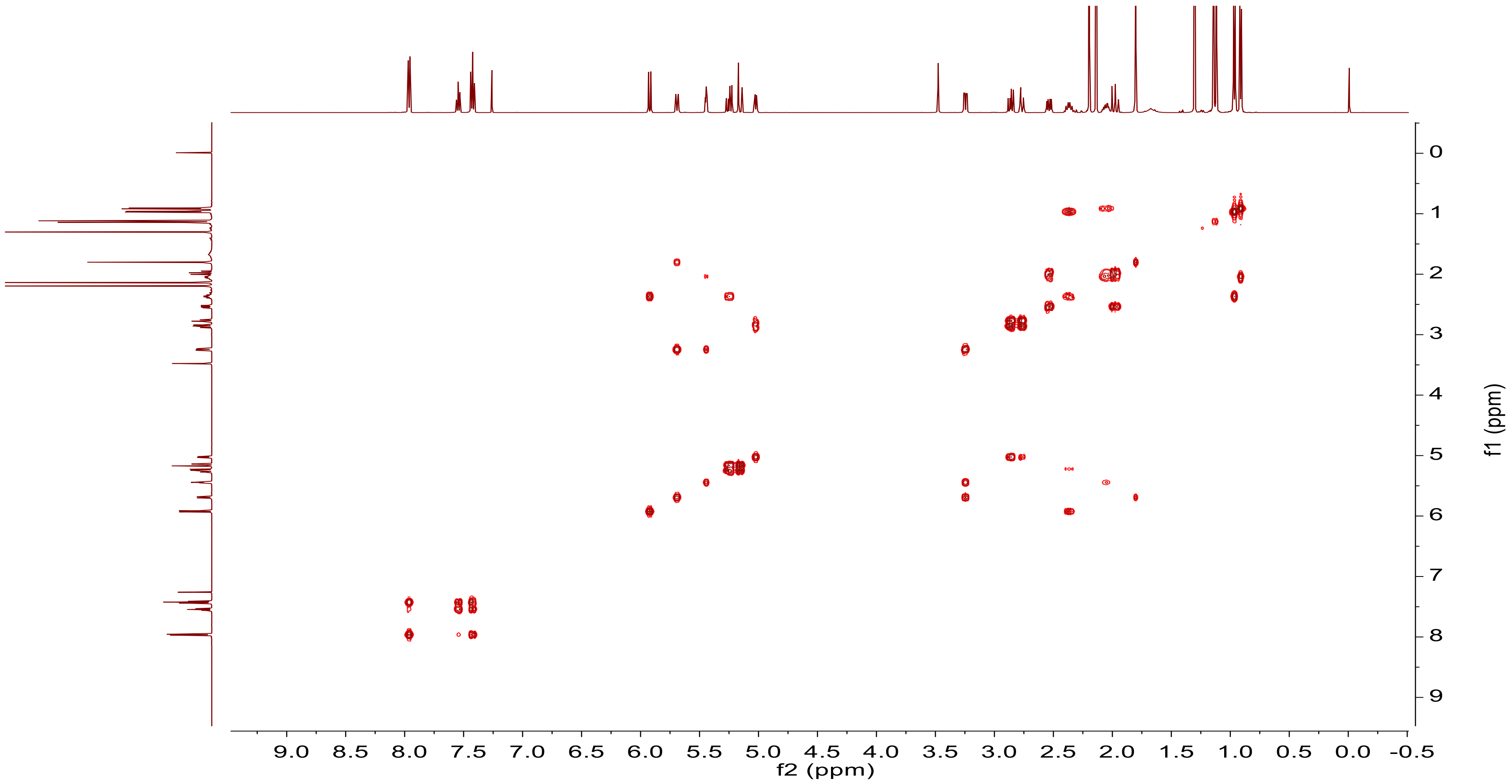

Figure S49. ^{1}H-^{1}H COSY spectrum of euphornin H in $CDCl_3$ (500 MHz)

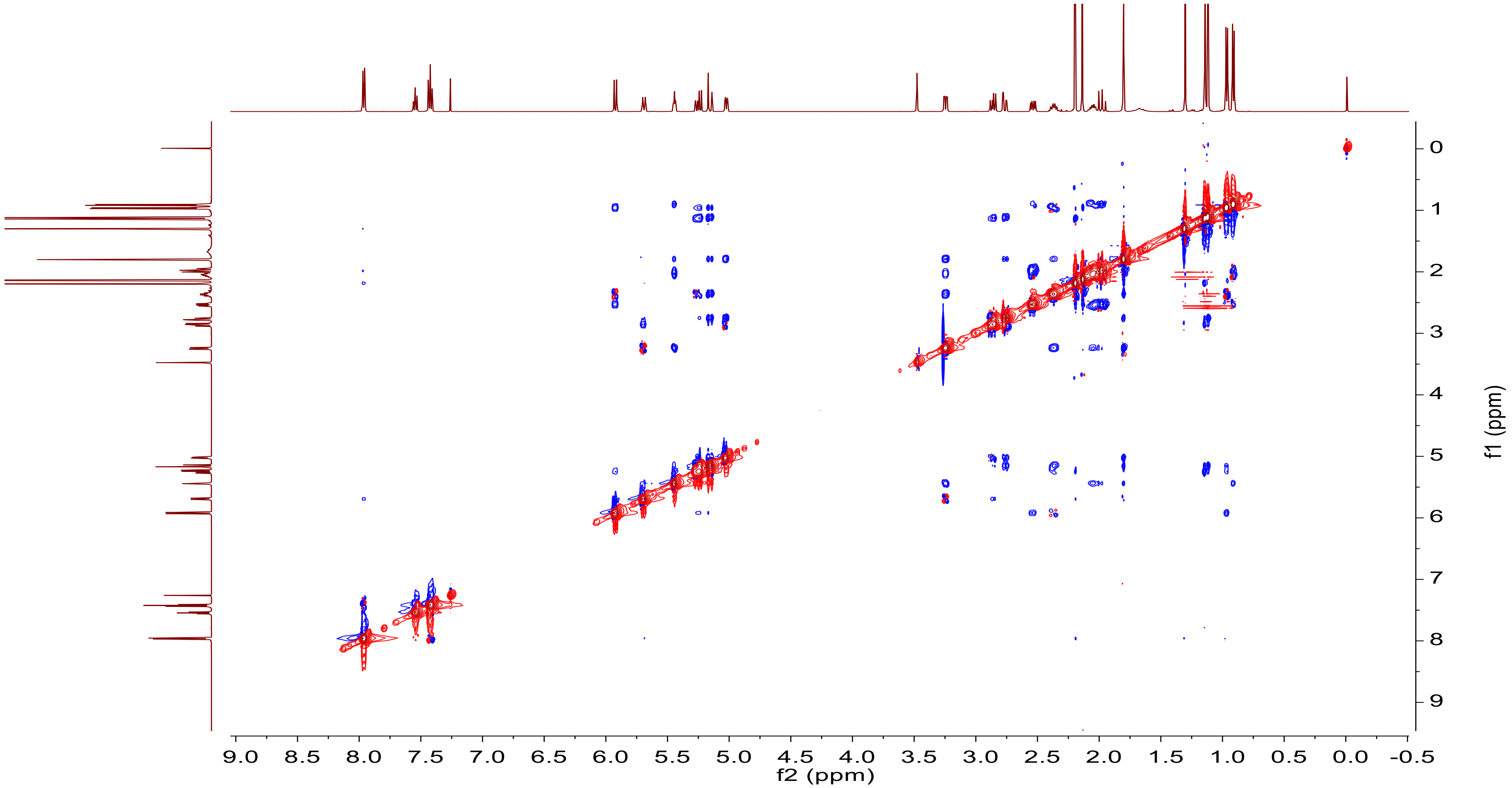

Figure S50. NOESY spectrum of euphornin H in $CDCl_3$ (500 MHz

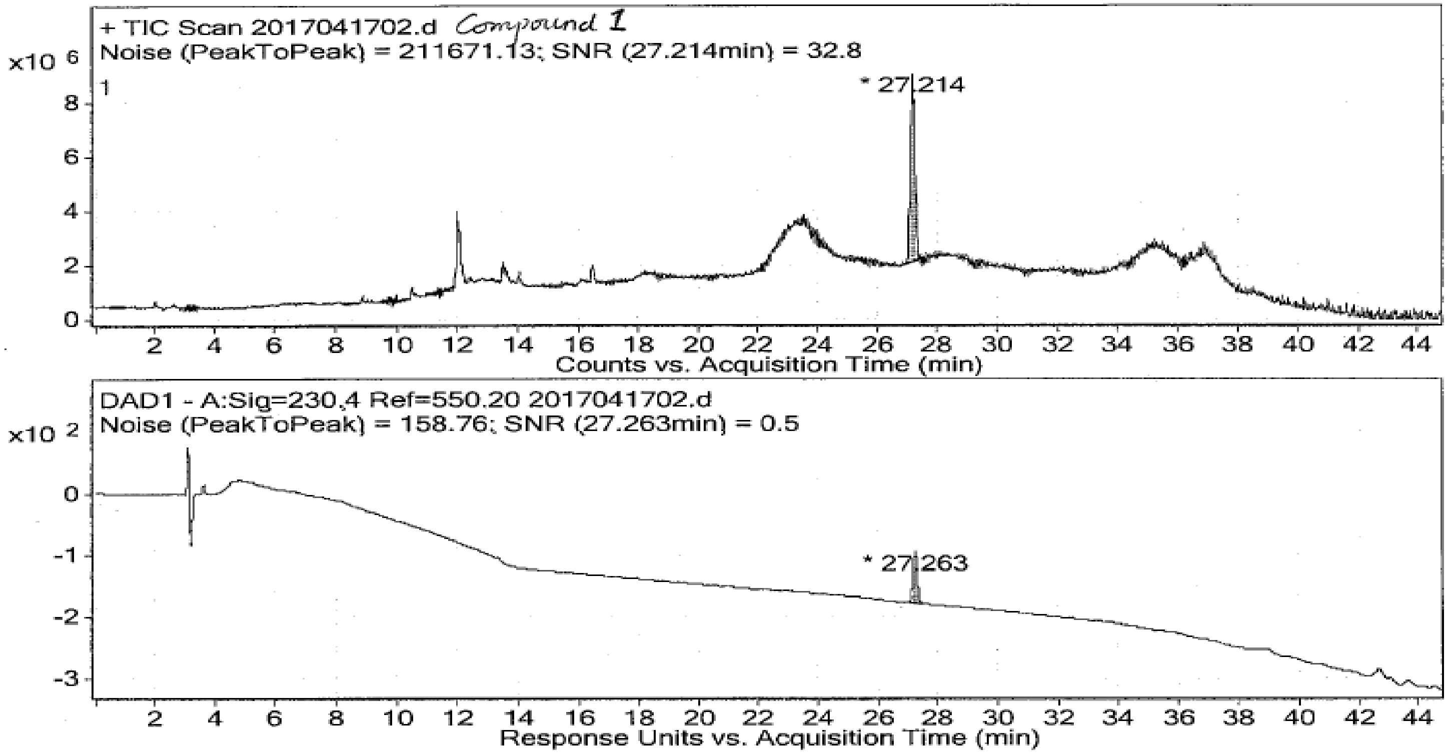

Figure S51. TIC and DAD spectra of compound **1** in LC-MS

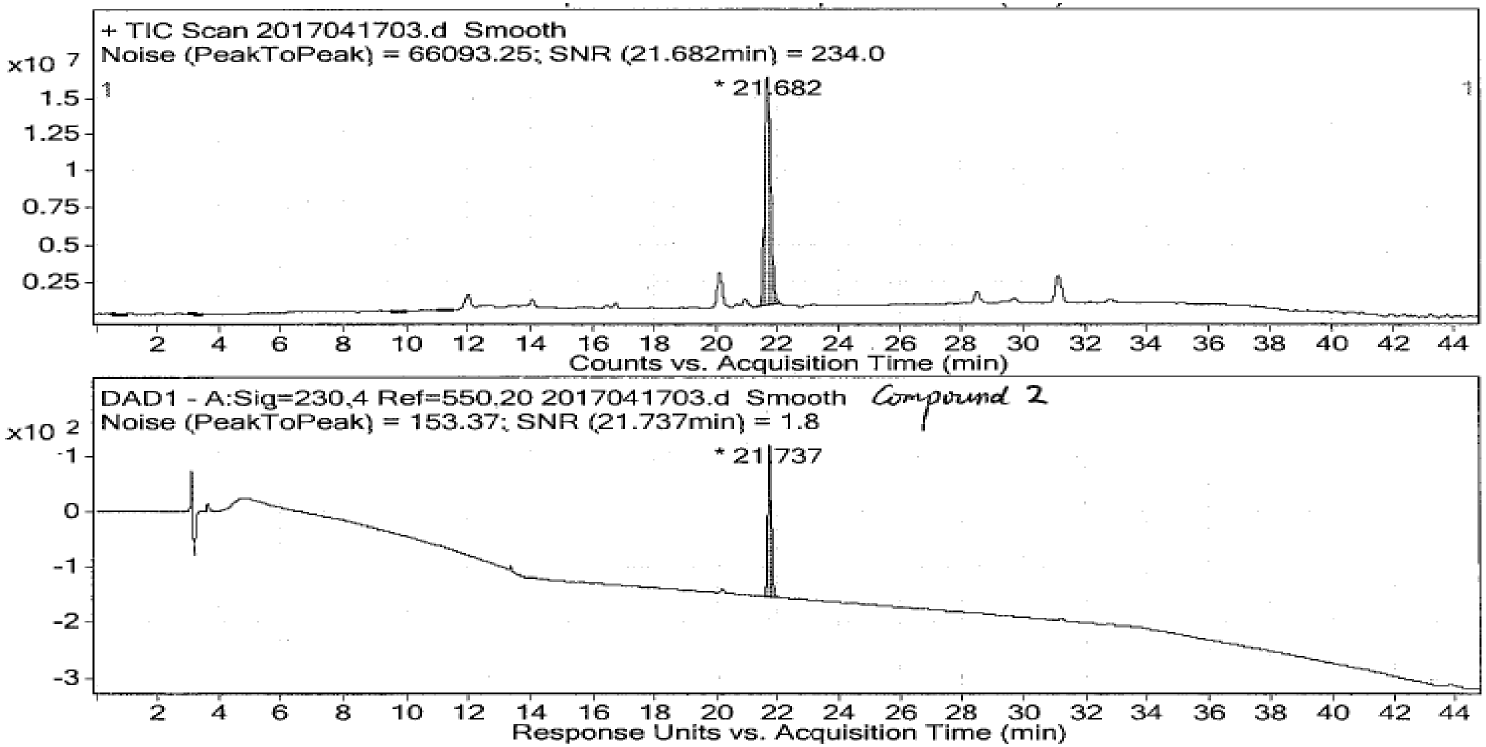

Figure S52. TIC and DAD spectra of compound **2** in LC-MS

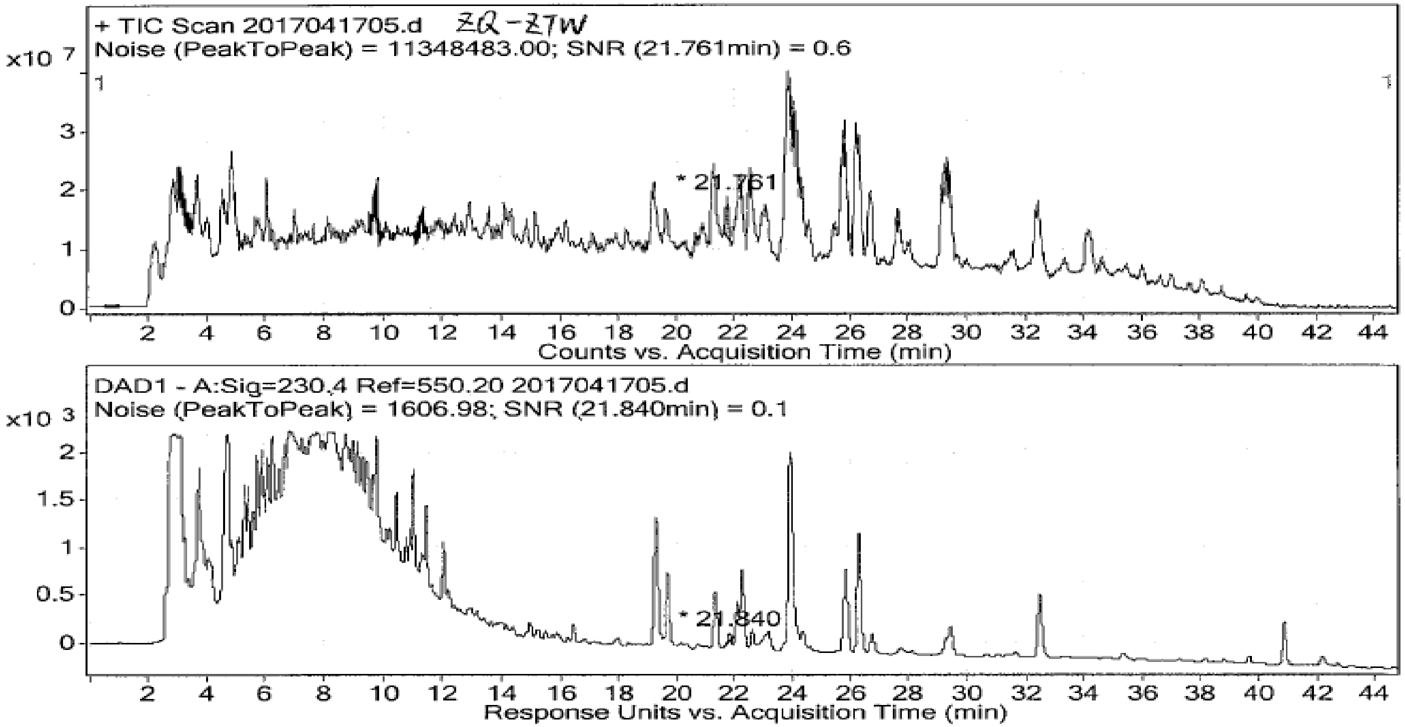

Figure S53. TIC and DAD spectra of the crude extract in LC-MS

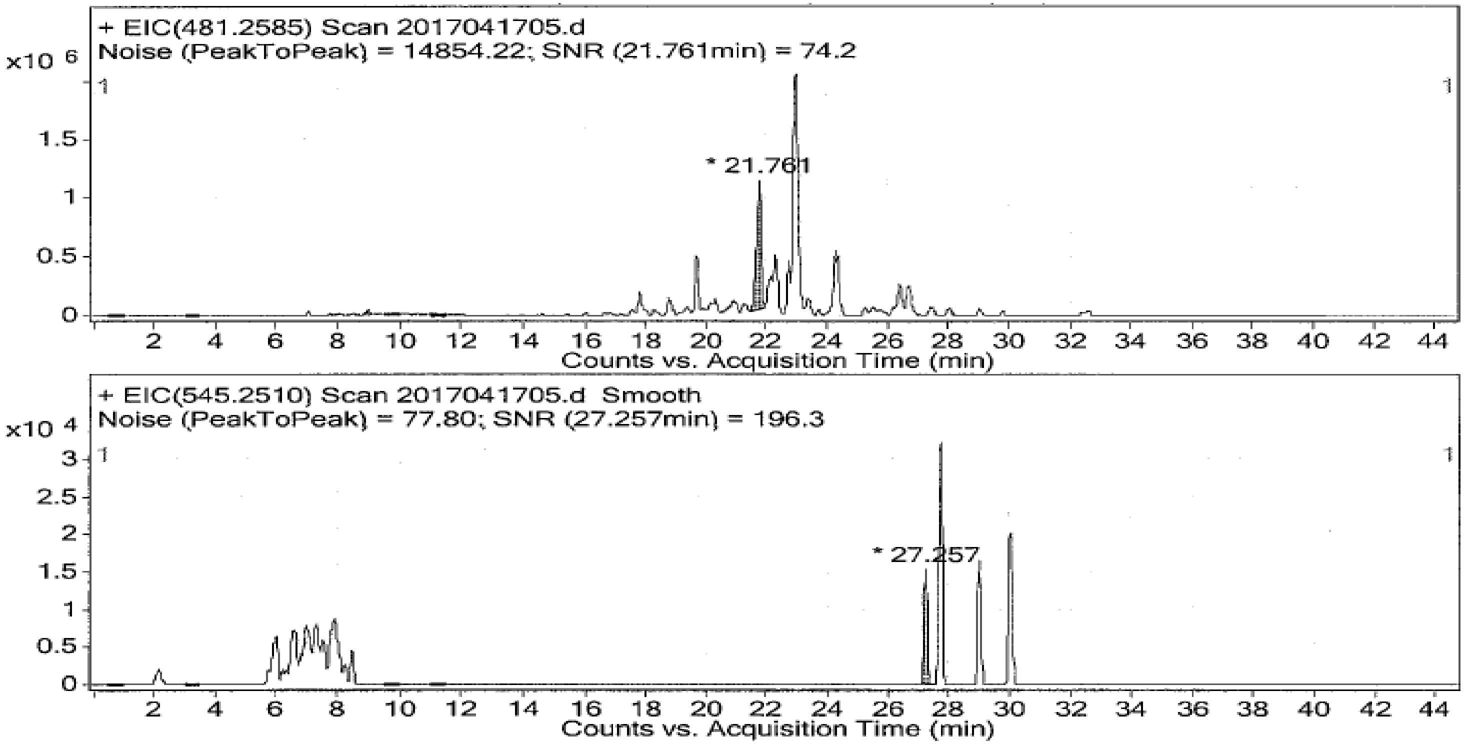

Figure S54. EIC spectra of compounds **1** and **2** in the crude extract by LC-MS

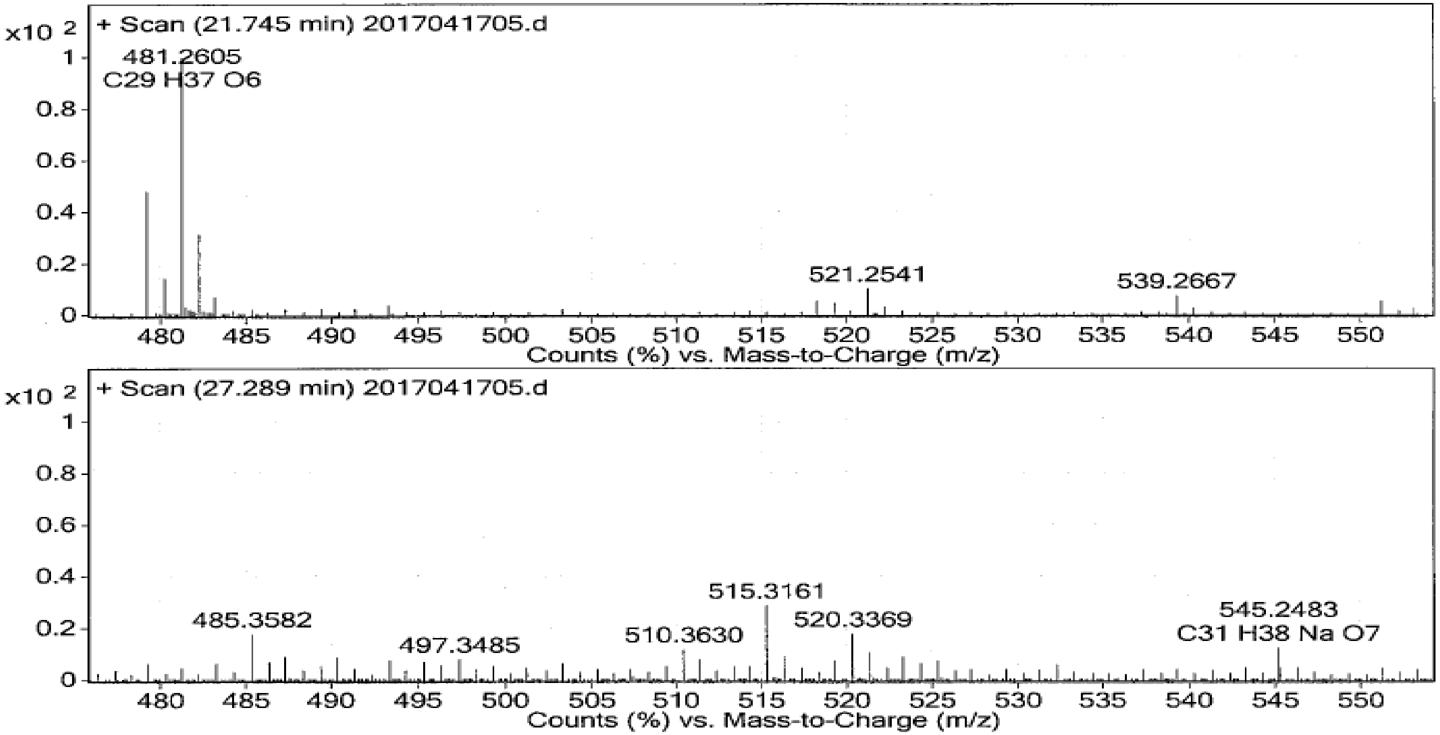

Figure S55. EIC spectra of compounds **1** (*m/z* 545.2483) and **2** (*m/z* 481.2605) in the crude extract by LC-MS
